# The Epiphyte *Bacillus* sp. G2112 Produces a Large Diversity of Nobilamide Peptides That Promote Biofilm Formation in Pseudomonads and *Mycobacterium aurum*

**DOI:** 10.3390/biom14101244

**Published:** 2024-10-01

**Authors:** Kenechukwu Iloabuchi, Dieter Spiteller

**Affiliations:** 1 Department of Chemical Ecology/Biological Chemistry, University of Konstanz, Universitätsstraße 10, 78457 Konstanz, Germany; kenechukwu.iloabuchi@uni-konstanz.de; 2 Department of Biochemistry, Faculty of Biological Sciences, University of Nigeria, Obukpa Road, Nsukka 410105, Nigeria

**Keywords:** antibiotics, *Lysinibacillus sphaericus*, mass spectrometry, *N*-acyldepsipeptide, secondary metabolites

## Abstract

*Bacillus* sp. G2112, an isolate from cucumber plants that inhibited plant pathogens, produces not only surfactins, iturins, and fengycins common to many *Bacillus* spp., but also a large variety of *N*-acyl-(depsi)peptides related to A-3302-B and nobilamides. Four known and fourteen previously unreported nobilamide peptides were characterized using high-resolution mass spectrometry, tandem mass spectrometry, and NMR. The stereochemistry of the amino acids of nobilamide peptides was determined using Marfey’s method. The diversity of nobilamide peptides from *Bacillus* sp. G2112 resulted from the incorporation of different acyl groups and amino acids in the sequence. The peptides occur in linear or cyclic form. In addition, a truncated *N*-acetylpentapeptide was produced. Agar diffusion assays with selected nobilamide peptides against plant pathogens and human pathogens revealed that A-3302-B and its *N*-acyl homologs, A-3302-A and nobilamide J, exhibited powerful antibiotic activity (at 5 µg/hole) against *Lysinibacillus sphaericus* that can cause severe sepsis and bacteremia in patients. Moreover, nobilamide peptides from *Bacillus* sp. G2112 strongly promoted biofilm formation in the Gram-positive *Mycobacterium aurum* and Gram-negative pseudomonads. Structurally diverse nobilamides from *Bacillus* sp. G2112, whether linear or cyclic, penta and heptapeptides, induced biofilm formation, suggesting that the common *N*-acetyl-D-Phe-D-Leu-L-Phe-D-allo-Thr-L-Val amino acid sequence motif is important for the biofilm-inducing activity.

## 1. Introduction

Secondary metabolites produced by microorganisms remain invaluable as either active principles or lead compounds for the development of drugs and crop protection solutions [1,2]. Moreover, over the last decades, our understanding of the roles of microbial secondary metabolites in their natural context has increased significantly. In nature, secondary metabolites serve many purposes, for example, defense, attack, nutrient acquisition, and intra- and interspecies communication [3,4,5,6]. As signals, secondary metabolites coordinate microbial community responses and orchestrate microbial differentiation, e.g., biofilm formation [7,8]. Understanding the biological functions of secondary metabolites can lead to identifying novel antibiotics and applications of natural products and their producing organisms. For example, secondary metabolites of plant-associated microorganisms can control plant pathogens and promote plant growth, hence such microorganisms are used in agriculture as biocontrol organisms [9,10,11,12].

Recently, we reported about an isolate from cucumber (*Cucumis sativus*) leaves, *Bacillus* sp. G2112, which strongly inhibited the cucumber pathogens *Fusarium equiseti* and *Erwinia tracheiphila* [13]. The secondary metabolite profile of *Bacillus* sp. G2112 obtained by LC-MS/MS revealed a remarkable diversity of non-ribosomal peptides. *Bacillus* spp. are well known to produce such peptides, in particular lipodepsipeptides. Lipodepsipeptides are produced by non-ribosomal peptide synthases and contain, in addition to *N*-terminal acyl moieties, amide and ester bonds [14]. More than 1300 depsipeptides have been identified. Many depsipeptides contain, in addition to proteinogenic L-amino acids, D-amino acids and rare amino acids [15,16]. Some depsipeptides are important as antibiotics, e.g., daptomycin [17,18], and as anticancer agents, e.g., romidepsin [19]. Moreover, lipodepsipeptides are crucial principles used by biocontrol organisms to protect crop plants from pathogens, e.g., the viscosin family [2,20]. In addition, depsipeptides constitute important antimicrobials in mutualistic symbioses of microorganisms and their host organisms, for example, sponges or insects [5,21,22,23].

The best-studied lipodepsipeptides from *Bacillus* spp. are surfactins. Surfactins comprise biosurfactants with rather weak antibiotic properties. However, surfactins were recognized to mediate physiological changes in *B. subtilis* and interactions with other microorganisms. Surfactins induce their own biofilm formation and could trap antagonistic compounds released by competitors [7,24,25].

**Figure 1:**
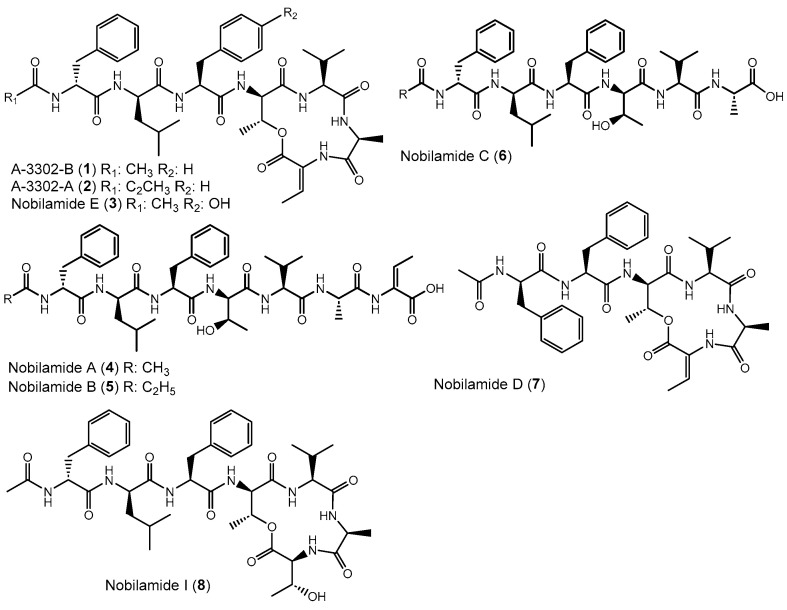
Examples of nobilamide-type secondary metabolites: A-3302-B (**1**) and A-3302-B (**2**), nobilamide E (**3**), as well as the linear *N*-acylheptapeptides, nobilamide A (**4**), nobilamide B (**5**), the truncated *N*-acylhexapeptide nobilamide C (**6**), and the truncated *N*-acylhexadepsipeptide nobilamide D (**7**), as well as nobilamide I (**8**).

Apart from intensively studied and widespread lipodepsipeptides, some *Bacillus* spp. like *Bacillus* sp. TL-119 produce lipodepsipeptides such as the uncommon *N*-acetylated heptadepsipeptide A-3302-B (**1**, also known as TL-119, Figure 1) which inhibit Gram-positive bacteria [26,27]. A-3302-B (**1**) strongly inhibited the growth of methicillin-resistant *Staphylococcus aureus* [28] and suppressed herpes simplex virus type-2 infection [29]. Eight other related peptides, named nobilamides A–H (for examples see Figure 1) were later identified from *Streptomyces* sp. and were found to antagonize capsaicin-induced calcium uptake in human and mouse brains [30]. Nobilamide I (**8**) from *Saccharomonospora* sp. suppressed cancer cell motility and expression of the matrix metalloproteinase-2 and -9 involved in epithelial–mesenchymal transition in lung, gastric, and colorectal cancers [31]. These studies highlight the interesting medicinal potential of nobilamide-type *N*-acyl(depsi)peptides. 

Here, we describe the identification of fourteen novel nobilamide peptides in addition to four previously reported ones from *Bacillus* sp. G2112. We evaluated their antibiotic potential against plant and human pathogens and their impact on biofilm formation. Potential ecological functions of nobilamide peptides for *Bacillus* sp. G2112 and consequences for biocontrol are discussed.

## 2. Materials and Methods

Chemicals: HPLC-grade methanol and acetonitrile were purchased from VWR (Darmstadt, Germany). Ethyl acetate and acetone were distilled from a technical-grade supply from Carl Roth (Karlsruhe, Germany). Media ingredients were from Carl Roth (Karlsruhe, Germany). Marfey’s reagent (1-fluoro-2-4-dinitrophenyl-5-L-alanine amide, FDAA) was from TCI (Tokyo, Japan). Glycine and D-/L-amino acids, including allo-threonine, were from Sigma-Aldrich (Taufkirchen, Germany) and TCI (Tokyo, Japan). Crystal violet (C.I. 42555) was from Carl Roth (Karlsruhe, Germany) and CD_3_OD was from Deutero GmbH (Kastellaun, Germany).

Instruments: Semi-preparative HPLC separation of the samples was performed using an Agilent 1100 HPLC system (Waldbronn, Germany) with a Gilson 206 fraction collector. Separations were performed either on a Synergi polar RP column (250 × 4.6 mm, 4 µm, Phenomenex, Aschaffenburg, Germany) or a Nucleodur C8 Gravity column (250 × 4.6 mm, 5 µm, Macherey-Nagel, Düren, Germany). For liquid chromatography–mass spectrometry (LC-MS), a Waters Acquity ultra-high-performance liquid chromatography (UPLC) system (Waters GmbH, Eschborn, Germany) equipped with a Synergi polar RP column (250 × 2.1 mm, 4 µm) was used. The UPLC was equipped with a photodiode array (PDA) detector and connected to an LTQ mass spectrometer (Thermofisher, San Jose, CA, USA) fitted with a heated electrospray ionization source (HESI II) operated in positive ionization mode. High-resolution electrospray mass spectrometry (HR-ESI-MS) was performed using an LTQ Orbitrap XL mass spectrometer (Thermofisher, Bremen, Germany) with a heated electrospray ionization source (HESI II) operated in positive ionization mode and connected to a Dionex Ultimate 3000 UPLC system fitted with Nucleodur C8 Gravity column (250 × 2 mm, 5 µm, Macherey-Nagel, Düren, Germany). Mass spectra were acquired after fresh calibration of the instrument at a resolution setting of 100,000 using the lock mass function.

Nuclear magnetic resonance (NMR) spectra were acquired using a Bruker Avance III 600 spectrometer (^1^H-NMR 600 MHz, ^13^C-NMR 151 MHz) or a Bruker Avance Neo 800 MHz (^1^H-NMR 800 MHz, ^13^C-NMR 201 MHz) equipped with a TCI-H/C/N triple resonance cryoprobe with Z gradient (Bruker, Rheinstetten, Germany). The NMR spectrometer was calibrated to residual CD_3_OD signals (^13^C 49.00, ^1^H 3.31 ppm) [32].

Microorganisms: *Bacillus subtilis* DSM10, *Bacillus pumilus* DSM27, *Bacillus thuringiensis* DSM 2046, *Bacillus amyloliquefaciens* DSM7, *Bacillus megaterium* DSM 1321, *Erwinia tracheiphila* DSM21139, *Lysinibacillus sphaericus* DSM1867, and *Mycobacterium aurum* DSM 43,999 were obtained from the German Collection of Microorganisms and Cell Cultures GmbH (Braunschweig, Germany). *Fusarium equiseti* FSU5459 was from the collection of the Friedrich Schiller University Jena. *Fusarium graminearum* was from Dr. Stefan Kunz from Bioprotect, Konstanz. Clinical isolates of *Staphylococcus aureus*, *Enterococcus faecalis*, *Klebsiella pneumoniae*, *Streptococcus pyogenes*, and *Vibrio cholerae* were generously provided by Prof. Dr. Christof Hauck, University of Konstanz. *Pseudomonas syringae* pv. *glycinea* 1a/96 was from Dr. Beate Völksch, Friedrich Schiller University Jena. *Bacillus* sp. G2112 and *Pseudomonas* sp. G124 were isolated from cucumber (*Cucumis sativus*) leaves from the island of Reichenau near Konstanz, Germany [13]. *Bacillus* sp. K13B and *Bacillus* sp. K29B were isolated from apple leaves in Konstanz, Germany.

Cultivation conditions: Microorganisms were cultivated on agar plates at 28 °C or in liquid medium in baffled Erlenmeyer flasks at 28 °C and 120 rpm (Infors, Multitron II orbital shaker, Einsbach, Germany). Either King’s B medium (KB, 20 g peptone, 10 g glycerol, 1.5 g K_2_HPO_4_, 1.5 g MgSO_4_ 7H_2_O, per L H_2_O, pH 7.0; for solid medium 20 g agar) [33], tryptic-soy broth (TSB, 30 g CASO bouillon, 15 g agar, per L ddH_2_O, pH 7.3) [34] or lysogeny agar (LB, 10 g tryptone, 5 g yeast extract, 5 g NaCl, 15 g agar, per L ddH_2_O, pH 7.0) [35] were used to cultivate the microorganisms. 

Screening for secondary metabolites of *Bacillus* sp. G2112: In order to study the secondary metabolites of *Bacillus* sp. G2112, the organism was cultivated in 5 mL KB liquid medium in three sterile test tubes for 14 d at 28 °C and 120 rpm. The centrifuged spent culture supernatant (15,000× *g*, 2 min, Eppendorf, Hamburg) was either directly analyzed or adjusted to pH 4 and extracted with ethyl acetate before LC-MS using an LTQ ion trap mass spectrometer. The data-dependent scan was employed for MS/MS fragment acquisition. The spectrum files were directly uploaded to the Global Natural Product Social Molecular Network website (GNPS; http://gnps.ucsd.edu, accessed on 7 December 2023) [36] and a molecular network was created using the default online workflow (https://ccms-ucsd.github.io/GNPSDocumentation accessed on 7 December 2023) with minor modifications as follows: precursor ion mass tolerance was set to 1.5 Da and MS/MS fragment ion tolerance to 0.05 Da. Edges of the network were filtered to have a cosine score above 0.6 and more than 4 matched peaks and edges between two nodes were retained only if each of the nodes appeared in each other’s respective top 10 most similar nodes. All matches kept between network spectra and library spectra were required to have a score above 0.7 and at least 4 matched peaks [36].

Time-course of nobilamide production by *Bacillus* sp. G2112: In order to determine when nobilamide peptides are produced, *Bacillus* sp. G2112 was cultivated in liquid KB medium. A 2 mL overnight (20 h) pre-culture of *Bacillus* sp. G2112 was diluted to OD_600_ 0.037 in fresh KB medium (100 mL) and dispensed as 10 mL cultures in three 25 mL flasks. The flasks were incubated at 28 °C and 120 rpm. Samples (200 µL) were withdrawn at 2 h, 1 d, 2 d, 3 d, and 4 d for OD_600_ measurement and for LC-MS analyses. For OD_600_ measurements, a 1:10 dilution was used for the first 24 h and a 1:50 dilution was analyzed afterwards. For LC-MS, the samples were centrifuged at 15,000× *g* for 2 min in a bench-top centrifuge, and 10 µL supernatant was injected into the LC-MS. LC-MS LTQ method: Synergi polar RP column (250 × 2.1 mm, 4 µm, Phenomenex, Aschaffenburg, Germany) using the following program: solvent A: water 0.1% AcOH, solvent B: MeCN 0.1% AcOH, flow rate: 0.22 mL/min, HPLC program: 2% B 1 min, gradient 2–100% B 22 min and 100% B 3 min. It was screened for the major nobilamide peptides, namely A-3302-B (1), A-3302-A (**2**) and nobilamide M (**12**), nobilamide L (**11**), nobilamide I (**8**), nobilamide O (**14**) and nobilamide P (**15**), nobilamide A (**4**), nobilamide S (**18**), and nobilamide W (**22**).

In addition, the timing of the formation of A-3302-B (1, [M + H]^+^ *m*/*z* 804.4) was compared to the production of the other three major peptide metabolite families produced by *Bacillus* sp. G2112, namely, surfactin C ([M + H]^+^ *m*/*z* 1036.7), iturin A ([M + H]^+^ *m*/*z* 1057.6), and fengycin A ([M + H]^+^ *m*/*z* 1463.8).

Production of nobilamides by different *Bacillus* strains: To determine whether the nobilamide peptides are commonly produced by *Bacillus* spp., culture supernatants (15,000× *g*, 2 min) from several bacilli incubated under the same conditions (see above) were analyzed by LC-MS. The sampled bacilli included *Bacillus subtilis*, *B. pumilus*, *B. thuringiensis*, *B. amyloliquefaciens*, *B. megaterium,* and two other bacilli isolated from apple leaves, *Bacillus* sp. K13B and *Bacillus* sp. K29B. Each *Bacillus* strain was cultivated in 5 mL KB medium in sterile test tubes for up to 14 d at 28 °C and 120 rpm in triplicate. The culture supernatants were harvested by centrifugation and directly analyzed by LC-MS.

Purification of nobilamide depsipeptides: A 2.5 L volume of KB spent medium of *Bacillus* sp. G2112 incubated at 28 °C and 120 rpm was harvested after 11 d by centrifugation. The pH was adjusted to 2.5 and left standing at room temperature for 4 h to precipitate some of the nobilamides. The spent medium supernatant was then centrifuged and both precipitate and supernatant were separately extracted with ethyl acetate. The ethyl acetate extracts were dried over anhydrous Na_2_SO_4_ and evaporated using a rotary evaporator. The dried extract of the supernatant (1 g) was purified on a column packed with Polygoprep 60-50 C18 resin (13.5 cm × 3.5 cm i.d., Macherey-Nagel, Düren, Germany) using sequential elution with 100 mL of 20%, 40%, 60%, 80%, and 90% aqueous MeOH. Final elution was performed with 500 mL MeOH and 20 mL fractions were collected per test tube to give fractions F1–F45. The fractions were freeze-dried and analyzed by LC-MS. Contiguous fractions containing similar nobilamide peptides were combined into three fractions (S1, S2, S3) and purified by semi-preparative HPLC using an Agilent 1100 HPLC system fitted with a Gilson fraction collector by iterative injection and collection of the fractions (ca. 3 injections per mg sample). S1 (F17–F23) was purified using a Synergi polar RP column (250 × 4.6 mm, 4 µm, Phenomenex, Aschaffenburg, Germany). HPLC conditions: solvent A: water 0.1% acetic acid and solvent B: MeCN 0.1% acetic acid, flow rate: 0.85 mL/min, HPLC program: 37% B 1 min, gradient 37–43% B 14 min, and 43–100% B 8 min. Fractions were collected from 6 min at 0.33 min/tube and lyophilized. S2 (F24–F26) was separated with a Nucleodur C8 Gravity column (250 × 4.6 mm, 5 µm, Macherey-Nagel, Düren, Germany), HPLC conditions: solvent A: water 0.1% acetic acid and solvent B: MeCN 0.1% acetic acid, flow rate: 0.8 mL/min, HPLC program: 38% B 1 min, 38–70% B 18 min and 70–100% B 4 min. Fractions were collected from 2 min at 0.5 min/tube and lyophilized. S3 (F38–F45) was separated using a Synergi polar RP column (250 × 4.6 mm, 4 µm, Phenomenex, Aschaffenburg, Germany). HPLC conditions: solvent A: water 0.1% acetic acid and solvent B: MeCN 0.1% acetic acid, flow rate: 0.8 mL/min, HPLC program: 45% B 1 min, 45–100% B 17 min. Fractions were collected from 0 min at 0.5 min/tube and freeze-dried. The concentrated ethyl acetate extract of the pH 2.5 precipitate (1.1 g) was dissolved in methanol and the methanol-soluble portion was directly separated with the same conditions used to separate fraction S1. Based on LC-MS, HPLC fractions H34–H38 from the precipitate were combined and resolved by a further HPLC separation using a Nucleodur C8 Gravity column (250 × 4.6 mm, 5 µm, Macherey-Nagel, Düren, Germany), HPLC conditions: solvent A: water 0.1% acetic acid and solvent B: MeOH 0.1% acetic acid, flow rate: 0.8 mL/min, HPLC program: 80% B 12 min, gradient from 80–100% B 10 min and isocratic 100% B 3 min. Fractions were collected from 2 min at 0.5 min/tube and freeze-dried.

Elucidation of structures of nobilamide peptides by HR-ESI-MS, MS/MS, and NMR: Purified compounds were separated on an analytical Nucleodur C8 Gravity column (250 × 4.6 mm, 5 µm, Macherey-Nagel, Düren, Germany) using a Dionex Ultimate 3000 UHPLC system. LC-HR-ESI-MS method: HPLC conditions were as follows: solvent A: water 0.1% AcOH, solvent B: MeCN 0.1% AcOH, flow rate: 0.2 mL/min, HPLC program: 30% B 1 min, gradient elution 30–100% B for 29 min and 100% B for 10 min. The HPLC was coupled to the LTQ Orbitrap XL mass spectrometer. HR-ESI-MS and MS/MS spectra were acquired. Mass spectra were analyzed using the Xcalibur Qualbrowser (Thermofisher Scientific). For selected purified compounds, NMR spectra (^1^H-NMR, ^13^C-NMR, ^1^H,^1^H-COSY, HSQC, and HMBC) were recorded (see Supplementary Materials).

Analysis of the stereochemistry of amino acids of nobilamide peptides by Marfey’s method [37,38]: Dried purified nobilamide peptides (0.2–0.5 mg) were re-suspended in 300 µL of 6N HCl in 1.5 mL Teflon-capped glass vials. The vials were incubated at 99 °C with shaking at 400 rpm for 14 h. The solvent was evaporated by rotary evaporation and the residue was treated with 200 µL of 1% Marfey’s reagent in acetone and 40 µL of 1 M sodium bicarbonate. The reaction mixture was incubated at 50 °C with shaking (400 rpm) for 2 h after which it was neutralized with 20 µL of 2 N HCl and analyzed by LC-MS. The HPLC conditions were as follows: Accucore RPMS column (150 × 2.1 mm, 2.6 µm, Thermo Scientific, Dreieich, Germany), solvent A: 10 mM ammonium acetate (pH 5.6), solvent B: MeCN; flow rate 250 µL/min. HPLC program: 17% B for 3 min, 17–40% B for 16 min, 40–98% B for 3 min, and 98% B for 1 min. A standard mix of D- and L-amino acids (5 µM) was derivatized similarly to the samples for comparison.

Agar diffusion assays with nobilamides: Selected nobilamides A-3302-B (**1**), nobilamide A (**4**), nobilamide I (**8**), nobilamides O (**14**) and nobilamide P (**15**) mix, nobilamide S (**18**), nobilamide U (**20**) and nobilamide V (**21**) mix, and nobilamide W (22) were initially tested for their potential to inhibit the growth of phytopathogens (*F. equiseti*, *F. graminearum*, *E. tracheiphila*, *P. syringae* pv. *glycinea*) and non-phytopathogenic strains (*Pseudomonas* sp. G124, *B. subtilis, B. amyloliquefaciens, B. megaterium,* and *B. thuringensis* and *L. sphaericus*). Testing was also carried out against selected Gram-positive human pathogens, namely, *S. aureus*, *S. pyogenes*, *E. faecalis*, *K. pneumonia*, *V. cholera*, and *M. aurum*. Overnight liquid cultures (50 µL) or suspensions and mycelia plugs from agar plates were spread on appropriate agar media. *F. equiseti*, *F. graminearum*, *E. tracheiphila*, *P. syringae* pv. *glycinea, Pseudomonas* sp. G124, *B. subtilis, B. amyloliquefaciens, B. megaterium, B. thuringensis, L. sphaericus,* and *M. aurum* were assayed on King’s B agar. *S. aureus*, *S. pyogenes,* and *E. faecalis* were assayed on TSB agar, and *K. pneumonia* and *V. cholerae* were assayed on LB agar. Holes (0.7 cm diameter) were punched into the agar with the flame-sterilized thick end of glass Pasteur pipettes. 

The nobilamide peptides were tested by applying 35 µL of 200 µg/mL (7 µg/hole), 50 µL of 1 mg/mL (50 µg/hole), and 50 µL of 2 mg/mL (100 µg/hole) methanolic test solutions into the holes. The agar plates were incubated at 28 °C (KB agar plates) or 37 °C (TSB and LB agar plates) for 1 to 4 d depending on the test organism. Methanol served as a control and two to three replicates were performed depending on the amount of test samples available. The plates were analyzed for inhibition zones and photos were taken for documentation of positive results. Further agar diffusion assays were carried out using the four major compounds (**1**, **4**, **8**, and **18**), which represented four important structural differences compared to A-3302-B (**1**). A 50 µL volume of each test solution of concentrations 0.001 mg/mL, 0.01 mg/mL, 0.1 mg/mL, 0.2 mg/mL, 0.4 mg/mL, 1 mg/mL, and 2 mg/mL (prepared by successive dilutions) corresponding to 0.05 µg, 0.5 µg, 5 µg, 50 µg, and 100 µg of the compounds per hole, respectively, were tested against *L. sphaericus*. Minimum and maximum inhibitory amounts of compound **1** against *L. sphaericus* were determined by testing 50 µL of test solutions corresponding to a range of 0.5 µg–20 µg per hole. Lastly, 5 µg/hole of compounds **2** and **9** (0.1 mg/mL test solutions) were tested in order to determine whether they share the same activity as **1**. All assays were carried out in triplicate and methanol served as control.

Effect of nobilamides on biofilm formation: In order to investigate whether the nobilamides from *Bacillus* sp. G2112 influence biofilm formation, peptide A-3302-B (**1**), nobilamide A (**4**), nobilamide I (**8**), nobilamide S (**18**), and nobilamide W (**22**) were tested against *Pseudomonas* sp. G124, *P. syringae* pv. *glycinea*, *B. amyloliquefaciens*, *L. sphaericus*, *M. aurum,* and *Bacillus* sp. G2112 using the method of Merritt et al. [39]. The five nobilamides were chosen to represent their structural variations, including heptapeptides [A-3302-B (**1**), nobilamide A (**4**), nobilamide I (**8**), and nobilamide S (**18**)] and truncated nobilamide W (**22**). Also, nobilamide lactones [A-3302-B (**1**) and nobilamide I (**8**)] and linear peptides [nobilamide A (**4**), nobilamide S (**18**), and nobilamide W (**22**)], as well as threonine-containing [nobilamide I (**8**) and nobilamide S (**18**)] and *Z*-dehydrobutyrine-containing nobilamides [A-3302-B (**1**) and nobilamide A (**4**)] were covered by this selection. The test strains were grown in KB medium at 28 °C for 1–2 d and diluted 1:100 in the same medium. An 80 µL volume of this diluted culture was pipetted into wells of 96-microtiter well plates and 20 µL of selected test compounds prepared as suspensions in KB medium (1 mg/mL) was added. Each treatment was performed in triplicate and each organism was tested in a separate microtiter plate. The plates were incubated at 28 °C in a stationary incubator for 2 d. To quantify biofilm production, planktonic bacteria were briskly removed from the microtiter wells, and the wells were washed twice by immersion in water. The plates were allowed to air-dry for 10 min. The wells were stained with 125 µL of 0.1% crystal violet in ddH_2_O for 10 min at room temperature. The crystal violet solution was removed by inverting the plates and unbound stains were gently washed off twice by immersion in water. The plates were then air-dried and 200 µL of 30% aqueous acetic acid was added into each well to solubilize the bound staining. The contents of the wells were mixed by pipetting and 125 µL of the staining solution was transferred into wells of fresh microtiter plates. Absorbance at 590 nm was recorded. 

Statistical analysis: Data analysis was performed using Microsoft Excel (Professional Plus 2016, version 2311). Means and standard deviations of all replicates of the same experiment were computed using the mean and sample standard deviation functions. Single-factor analysis of variance was carried out using all the replicate measurements, including medium control (*p* < 0.05). Once significant variation at *p* < 0.05 was established, a pairwise analysis between the treatments and the treatment control was made using the *t*-test: two-sample assuming equal variance method with *p* < 0.05. Finally, a Bonferroni correction was conducted to analyze for significance (*p* < 0.01).

## 3. Results

### 3.1. Production of Nobilamide-Type N-acylpeptides by Bacillus sp. G2112

**Figure 2:**
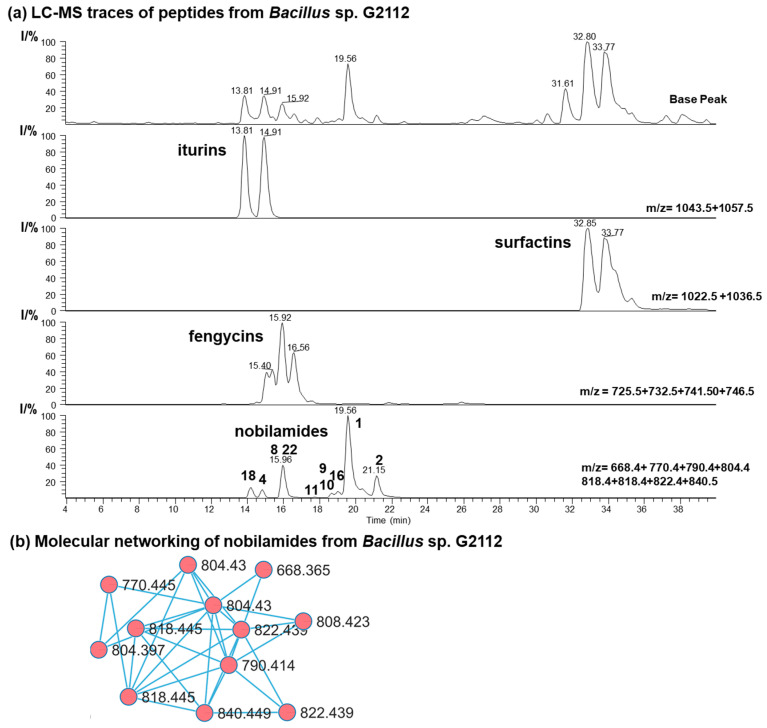
Secondary metabolites of *Bacillus* sp. G2112. (**a**) HPLC base peak chromatogram and combined ion trace chromatograms (HPLC method: LC-HR-ESI-MS) of some secondary metabolites in spent King’s B medium supernatant of *Bacillus* sp. G2112 after 6 d of growth. Major nobilamides are indicated in the combined ion trace chromatogram by their number. (**b**) Molecular networking analysis of nobilamide peptides in the ethyl acetate extract (pH 2.5) of spent medium of *Bacillus* sp. G2112.

Secondary metabolites from the cucumber epiphyte, *Bacillus* sp. G2112, were investigated because *Bacillus* sp. G2112 exhibited promising activity against plant pathogens [13]. *Bacillus* sp. G2112 was cultivated in King’s B medium and the spent medium supernatant was analyzed by LC-MS/MS. *Bacillus* sp. G2112 produced a large variety of peptides, including surfactin C12–C15 (*m*/*z* 994.6–1036.7), iturin A C13–C16 (*m*/*z* 1029.5–1071.6), fengycin A C14–C19 (*m*/*z* 1435.8–1505.9) [40,41], as well as many nobilamides with *m*/*z* between 668.4 and 846.4 (Figure 2 and Figure S1). High-resolution ESI mass spectrometry, tandem mass spectrometry, and molecular networking analysis [36] were used to identify the peptides from *Bacillus* sp. G2112. We then focused on identifying the rarely occurring nobilamide peptides because *Bacillus* sp. G2112 appeared to produce many of these. The new nobilamide peptides produced by *Bacillus* sp. G2112 were named nobilamide J to nobilamide W in continuation of the naming convention initiated by Lin et al. [30] and followed by Le et al. [31]. 

**Figure 3:**
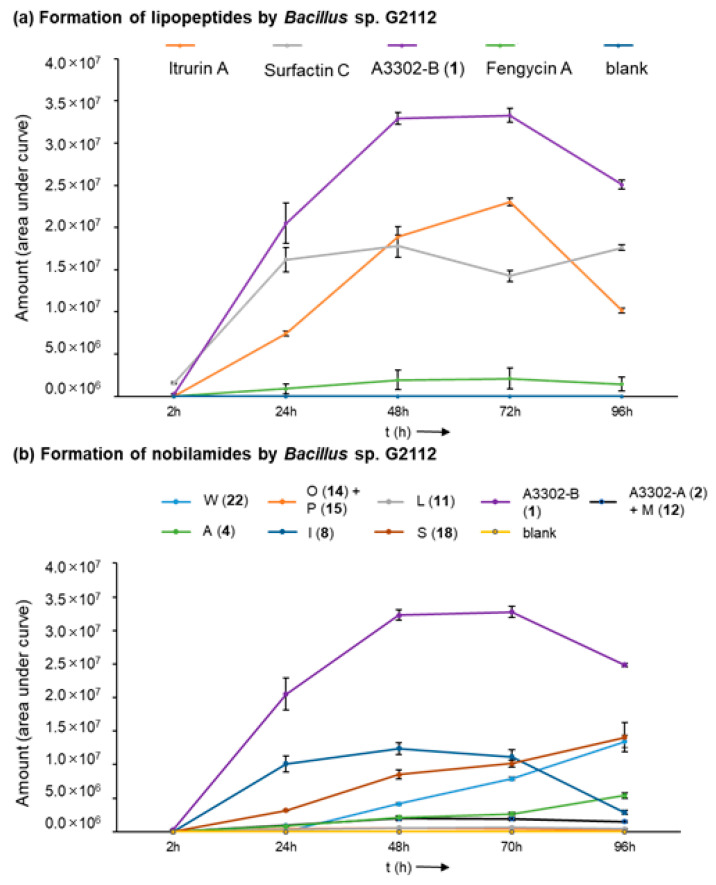
Time course of the formation of lipopeptides by *Bacillus* sp. G2112 over 4 d. (**a**) Formation of iturin A, surfactin C, fengycin A, and A3302-B (**1**). (**b**) Time course of the generation of selected nobilamides (indicated by letter and number in legend above graph).

*Bacillus* sp. G2112 reaches its stationary growth phase after 3 d (Figure S2). The time course of the formation of the peptides by *Bacillus* sp. G2112 revealed that peak production of predominant surfactins (surfactin C15, [M + H]^+^ 1036.7), fengycins (fengycin A C16, [M + H]^+^ 1463.7), and nobilamides (A-3302-B (**1**), [M + H]^+^ 804.4) is attained within 2 d, while iturin production (iturin A C15, [M + H]^+^ 1057.6) reached its maximum at day 3 (Figure 3a and Figure S3). Surfactins and nobilamide A-3302-B (**1**) were already present in samples analyzed at 2 h, while iturins and fengycins were only detected from 24 h onwards.

Production of the major nobilamides, namely A-3302-B (**1**), nobilamide A-3302-A (**2**) and nobilamide M (**12**), nobilamide L (**11**), nobilamide I (**8**), nobilamide O (**14**) and nobilamide P (**15**), nobilamide A (**4**), nobilamide S (**18**), and nobilamide W (**22**) by *Bacillus* sp. G2112 was studied (Figure 3b and Figure S4). The major *N*-acetyl-heptadepsipeptide A-3302-B (**1**) was detected earliest at 2 h of growth. The other nobilamides were produced after 1 d, except the pentapeptide nobilamide W (**22**), which was detected on day 2 (Figure 3b and Figure S4). Interestingly, the nobilamide lactones [A-3302-B (**1**), A-3302-A (**2**) and nobilamide M (**12**), nobilamide L (**11**), nobilamide I (**8**), nobilamide O (**14**) and nobilamide P (**15**)] peaked between d 2 and d 3 after which their relative amounts began to decline, while the linear nobilamides [nobilamide A (**4**), nobilamide S (**18**), and nobilamide W (**22**)] continued to increase throughout the 4 days of sampling (Figure 3b and Figure S4), indicating that the latter may constitute hydrolysis and breakdown products of the nobilamide lactones. 

In order to determine how widespread nobilamide production is among *Bacillus* species, we screened eight *Bacillus* strains for nobilamide production using LC-MS. However, only *Bacillus* sp. G2112 produced nobilamide peptides as shown by the combined ion trace chromatogram comparison of A-3302-B (**1**) and nobilamide W (**22**) in Figure S5.

### 3.2. Elucidation of the Structures of Nobilamide Peptides from Bacillus sp. G2112

The nobilamide peptides were extracted from the culture supernatant of *Bacillus* sp. G2112 after 11 d of growth in King’s B medium. Adjustment of the pH of the culture supernatant to 2.5 led to the precipitation of some nobilamides. The precipitate was extracted with ethyl acetate and the mixture was separated by HPLC using a Phenomenex Synergi polar RP column yielding A-3302-B (**1**), A-3302-A (**2**), nobilamide J (**9**), nobilamide K (**10**), nobilamide L (**11**) and nobilamide M (**12**). The rest of the nobilamide peptides were more abundant in the supernatant that was extracted with ethyl acetate. The nobilamide peptides eluted with >60% MeOH from the RP18 column and the nobilamide-containing fractions were combined in three samples (S1–S3) and further separated by HPLC. Separation of fraction S1 (F17–F23) by polar RP HPLC yielded nobilamide A (**4**), nobilamide S (**18**), nobilamide T (**19**), nobilamide U (**20**), nobilamide V (**21**), and nobilamide W (**22**). Separation of S2 (F24–F26) by RP8 HPLC yielded nobilamide A (**4**), nobilamide T (**19**), and a mixture of nobilamide C (**6**), nobilamide I (**8**), nobilamide N (**13**), nobilamide R (**17**), nobilamide S (**18**), and nobilamide W (**22**). The nobilamide mixture was resolved by an additional RP8 HPLC separation using isocratic elution with 42% MeCN 0.1% acetic acid. Separation of S3 (F38–F45) yielded A-3302-B (**1**), nobilamide L (**11**), nobilamide O (**14**) and nobilamide P (**15**), nobilamide Q (**16**), nobilamide R (**17**), and nobilamide S (**18**). However, the nobilamide pairs **14** and **15**, and **20** and **21** could not be resolved. 

From a 2.5 L culture of *Bacillus* sp. G2112 in KB medium, 7 mg A3302-B (**1**), 4 mg nobilamide A (**4**), 2.2 mg nobilamide I (**8**), 0.8 mg nobilamide J (**9**), 1 mg nobilamide L (**11**), 4.8 mg nobilamide S (**18**), and 3.8 mg nobilamide W (**22**) were obtained. The other nobilamides were produced in minor amounts.

**Figure 4:**
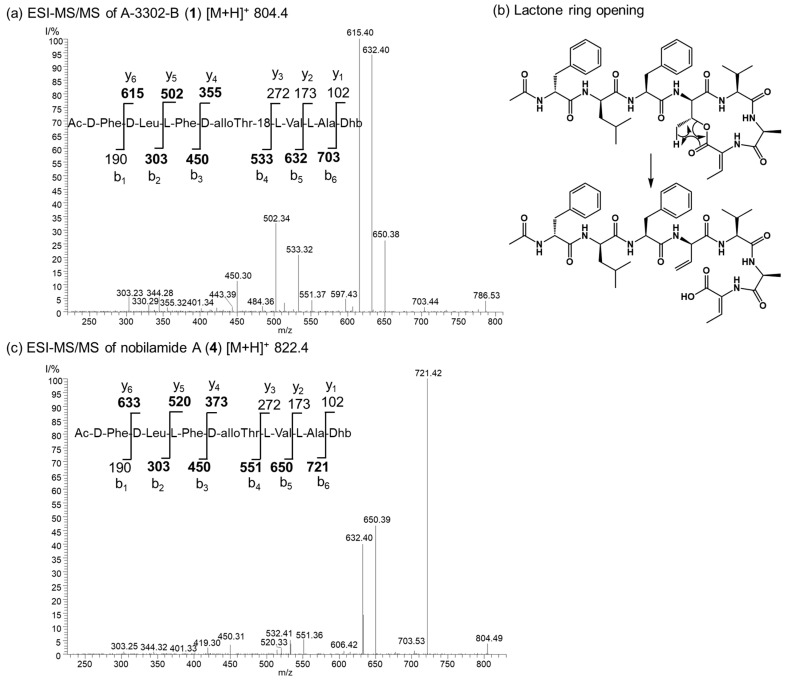
Comparison of the ESI MS/MS spectra of the lactone A-3302-B (**1**) and the corresponding linear nobilamide A (**4**). (**a**) MS/MS of the quasimolecular ion *m*/*z* 804.4 of A-3302-B (**1**). The structure of **1** and the y and b ions fragments of **1** after initial ring opening of the ester yielding allo-Thr-18 are shown. Observed fragments are highlighted in bold. Dhb: *Z*-α,β-dehydrobutyrine. (**b**) The generation of the allo-Thr-18 is depicted. Rearrangement and ester opening result in a double bond in the threonine at position four of the peptide sequence. This fragmentation reaction of the [M + H]^+^ quasimolecular ion allows distinguishing between cyclic and linear nobilamides by their MS/MS fragmentation pattern in combination with the results from the Marfey analysis of the individual amino acids (see SI). (**c**) MS/MS of the quasimolecular ion *m*/*z* 822.4 of nobilamide A (**4**). The structure of **4** and the y and b ions fragments of **4** are shown. Observed fragments are highlighted in bold. Dhb: *Z*-α,β-dehydrobutyrine.

The nobilamide peptides produced by *Bacillus* sp. G2112 were identified based on their molecular composition deduced by high-resolution mass spectrometry of the quasimolecular ions, their MS/MS fragmentation patterns and, when possible, NMR analyses. The stereochemistry of the amino acids of the nobilamide peptides was determined after acid hydrolysis using Marfey’s reagent [37,38]. Most nobilamides are *N*-acylheptapeptides with the general sequence *N*-acyl-D-phenylalanine-D-leucine-L-phenylalanine-D-allo-threonine-L-valine-L-alanine-(*Z*-dehydrobutyrine/L-threonine) [26,30]. Nobilamide peptides may either be linear or occur as lactones with the internal ester bond formed between the C-terminal α,β-dehydrobutyrine/threonine and D-allo-threonine at position four in the peptide sequence. Nobilamides can be identified by their characteristic MS/MS fragment ions. In combination with the Marfey analysis of the individual amino acids, it is straightforward to distinguish between the nobilamide lactones and the linear nobilamides because the [M + H]^+^ quasimolecular ion of the nobilamide lactones undergoes lactone ring opening converting the fourth amino acid allo-threonine into allo-threonine-18 resulting in -18 (H_2_O) of the respective peptide fragments (Figure 4). 

### 3.3. Nobilamides with Varying Acyl Chains

**Figure 5:**
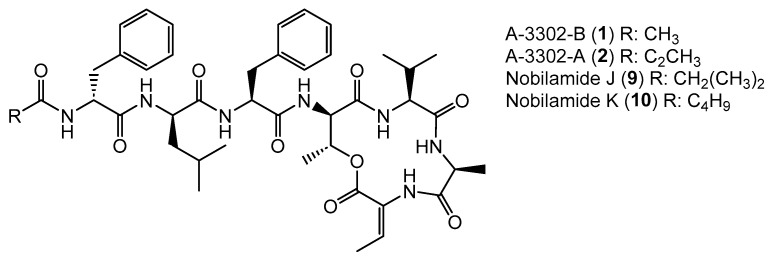
Nobilamides **1**, **2**, **9**, and **10** vary in their acyl moieties.

*Bacillus* sp. G2112 produces the *N*-acylheptadepsipeptides **1**, **2**, **9**, and **10** that vary in their acyl moieties: **1** bears an *N*-acetyl moiety, **2** contains a *N*-propionyl residue at the *N*-terminal phenylalanine; **1** and **2** were identified as A-3302-B and A-3302-A, respectively (Figure 3 and Figures S6–S17). Their spectroscopic data matched the published data [26]. Nobilamide J (**9**) and nobilamide K (**10**) are closely related to **1** but differ by 28 and 42 amu in the acyl moiety, respectively (Figure 5). The acyl residue of **9** was identified as an isobutanoyl moiety (Figures S18–S26) and that of **10** as the C_5_H_9_O acyl group (Figures S27–S29). MS/MS, NMR, and peptide hydrolysis followed by derivatization with Marfey’s reagent established the peptide sequence: **1**, **2**, **9**, and **10** have *Z*-dehydrobutyrine (*Z*-Dhb) at position seven and occur as lactones. The presence of the *Z*-Dhb residue was confirmed by the characteristic loss of 101 amu from the [M + H]^+^ ion (Figures S7, S16, S19 and S28). Moreover, in the ^1^H NMR, the characteristic β methine hydrogen of Z-Dhb at 6.85 ppm was present [37,41].

Generally, the occurrence of the nobilamides as lactones can be identified from their MS/MS fragmentation patterns. When the [M + H]^+^ quasimolecular ion undergoes lactone ring opening (Figure 4), the loss of H_2_O is reflected in pronounced b_4_ and b_3_ fragments, with the distance between the b_4_ to b_3_ fragments of 83 amu corresponding to an allo-Thr-H_2_O residue (Figures S7, S16, S19 and S28). In addition, the ^1^H-NMR signal of the β hydrogen of D-allo-Thr at position 4 of the lactone-containing peptide gets shifted to 4.26–4.67 ppm (compared to 3.89–3.97 for linear peptides, see NMRs of **1** and **9**, Figures S9, S11, S21 and S23) [30]. Nobilamide J (**9**) and nobilamide K (**10**) have not been previously reported.

### 3.4. Nobilamides Varying in the 6th and 7th Amino Acid Residue

**Figure 6:**
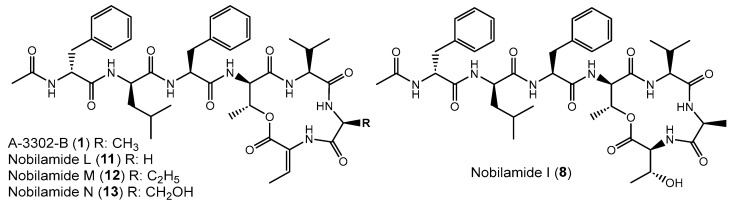
Nobilamides **8**, **11**–**13** vary in the 6th and 7th amino acids compared to A-3302-B (**1**).

Nobilamide L (**11**), nobilamide M (**12**), and nobilamide N (**13**) differ from A-3302-B (**1**) in the 6th amino acid residue (Figure 6). The presence of glycine instead of L-alanine in nobilamide L (**11**) was confirmed by MS/MS, NMR, and analysis of the amino acids of the peptide hydrolysis and derivatization with Marfey’s reagent (Figures S30–S38). In addition, similar to A-3302-B (**1**), the ^1^H-NMR of **11** exhibited a characteristic proton signal at 4.30 ppm for the β hydrogen of D-allo-threonine in position four (Figures S33 and S35). The β hydrogen methine shift at 6.69 ppm confirmed that **11** contained both the lactone ring and *Z*-Dhb at position seven [30].

Nobilamide M (**12**) was determined by MS/MS and Marfey’s analyses (Figures S39–S41) to contain homo-L-alanine instead of L-alanine in A-3302-B (**1**) because the residue in this position is 14 amu (methylene group) more than alanine. The stereochemistry of the homoalanine was not determined but tentatively assigned as L-isomer in analogy to the L-alanine that is normally found in this position. 

Likewise, nobilamide N (**13**) was determined by MS/MS and analysis of the amino acids using Marfey’s method after acidic peptide hydrolysis (Figures S42–S44) to contain L-serine instead of L-alanine in position six. Nobilamides L (**11**), nobilamide M (**12**), and nobilamide N (**13**) have not been previously reported.

Nobilamide I (**8**) was identified based on its MS/MS and NMR data and Marfey’s analysis of the amino acids (Figures S45–S53) to be the nobilamide I that was previously identified by Le et al. [31]. Nobilamide I (**8**) differs from A-3302-B (**1**) by having L-threonine instead of *Z*-Dhb at position seven of the amino acid sequence (Figure 6).

### 3.5. Variations in Positions of Leucine and Phenylalanine Residues in Nobilamides

**Figure 7:**
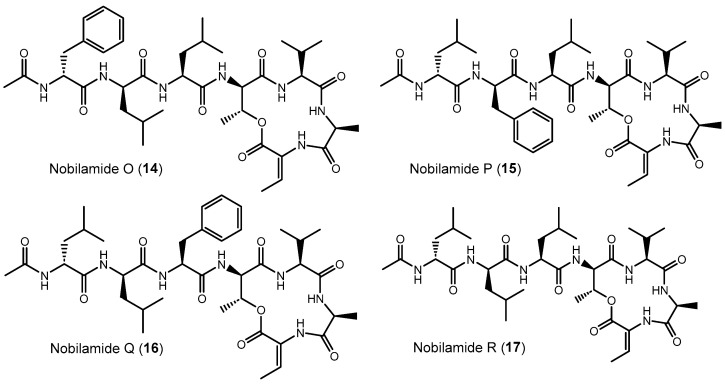
Exchange of phenylalanine against leucine and leucine against phenylalanine in nobilamides **14**–**17** compared to A-3302-B (**1**). The stereochemistry of the amino acids was deduced after peptide hydrolysis using Marfey’s reagent. The position of redundant D- and L-amino acids (phenylalanine and leucine) was assumed to follow the stereochemistry of the amino acid positions of A-3302-B (**1**).

Nobilamide O (**14**) (Figures S54–S56), nobilamide P (**15**) (Figures S54–S56), nobilamide Q (**16**) (Figures S57–S59), and nobilamide R (**17**) (Figures S60–S62) differed from A-3302-B (**1**) by the replacement of one or two phenylalanine residues at the 1st and 3rd positions in the *N*-acyl-heptapeptide sequence with leucine, as well as by the exchange of the positions of the remnant phenylalanine and leucine residues in the peptide sequence (Figure 7). All four compounds, which were produced in relatively low amounts, were established by their MS/MS fragmentation pattern to contain both Dhb at position seven ([M + H]^+^ − 101 amu) and a lactone ring (distance between b_4_–b_3_ ion of 83 amu corresponding to Thr-H_2_O) (Figures S55, S58 and S61).

The isomers nobilamide O (**14**) and nobilamide P (**15**) were obtained as a mixture of minor compounds that could not be fully resolved by HPLC (Figures S54 and S56). MS/MS analysis (Figure S55) indicated that nobilamide O (**14**) and nobilamide P (**15**) differed in their MS/MS fragmentation pattern of the [M + H]^+^ only by their y_6_ ions at *m*/*z* 581 for **14** and *m*/*z* 615 for **15**. Consequently, the peptide sequence of **14** was deduced to have phenylalanine as *N*-terminal amino acid followed by leucine, whereas **15** starts with *N*-terminal leucine followed by phenylalanine. Moreover, both **14** and **15** differed from A-3302-B (**1**) by having leucine as the third amino acid instead of phenylalanine (Figure 7). The stereochemistry of the amino acids was determined after peptide hydrolysis using derivatization with Marfey’s reagent (Figure S56). The position of the D-leucine and L-leucine in the peptide is suggested to be analogous to the stereochemistry in A-3302-B (**1**) and thus depicted like this in Figure 7.

Nobilamide Q (**16**) (Figure 7 and Figure S57–S59) differs from A-3302-B (**1**) by having the *N*-terminal phenylalanine replaced with leucine, as deduced from its MS/MS fragmentation pattern (Figure S58). Peptide hydrolysis followed by Marfey’s analysis confirmed that **16** contains only L-phenylalanine, as well as L-leucine and D-leucine (Figure S59). L-leucine and D-leucine could occupy either the first two positions [D-Leu (1)-L-Leu (2)-L-Phe (3) or L-Leu (1)-D-Leu (2)-L-Phe (3)]. The structure of nobilamide Q (**16**) is given in Figure 7 with the most likely stereochemistry deduced in analogy to A-3302-B (**1**), assuming that the stereochemistry of the amino acids is also maintained after the exchange of the amino acids.

Nobilamide R (**17**) (Figures S60–S62) contains no phenylalanine residue as determined by both MS/MS (Figure S61) and acid peptide hydrolysis followed by Marfey’s analysis (Figure S62). The ratio of the peaks of D-Leu and L-Leu was two to one. Hence, for nobilamide R (**17**), the structure with D-Leu (1)-D-Leu (2)-L-Leu (3) is suggested; this conserves the configuration at these positions in analogy to A-3302-B (**1**) (Figure 7 and Figure S62).

### 3.6. Linear Nobilamides

**Figure 8:**
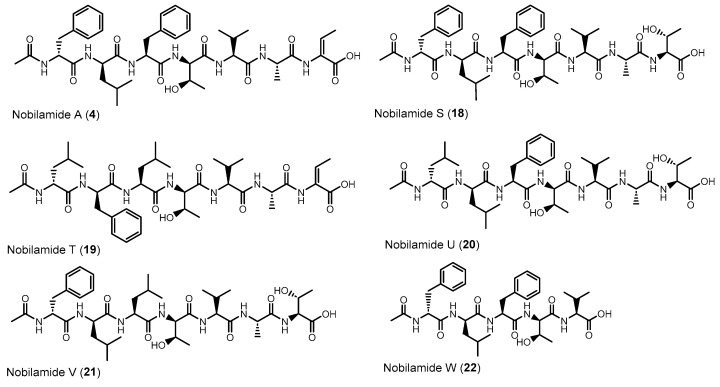
Linear nobilamides **4**, **18**–**21** and the truncated linear nobilamide W (**22**).

Nobilamides **18** and **19** are linear peptides that are related to the cyclic nobilamides **8** and **15** because of their identical amino acid sequences (Figure 8). The linear nobilamides **18**–**22** can be detected directly in culture supernatants, indicating that they are not generated during sample preparation. Linear nobilamides can be identified by their MS/MS fragment distance b_4_-b_3_ = 101 (allo-Thr) (Figure 4) and by NMR because of the characteristic shift of the β hydrogen of D-allo-Thr at 3.89–3.97.

Compound **4** was identified by MS/MS, NMR, and peptide hydrolysis followed by Marfey’s analysis to be identical to nobilamide A (Figures S63–S71), which was previously identified from *Streptomyces* sp. CN48 and *Streptomyces* sp. CT3a [30] as linear *N*-acetylheptapeptide variant of the cyclic A-3302-B (**1**). 

Nobilamide S (**18**) (Figures S72–S80) differs from the cyclic nobilamide I (**8**) by 18 amu (H_2_O) and MS/MS fragmentation revealed a distance of b_4_-b_3_ ions of 101 (D-allo-Thr) for nobilamide S (**18**) (Figure S73). Marfey’s analysis of the amino acids of nobilamide S (**18**) (Figure S74) revealed the same amino acids as in nobilamide I (**8**). In the ^1^H NMR, the shift of the β-hydrogen of D-allo-Thr at 3.89 ppm for nobilamide S (**18**) compared to the β-hydrogen at 4.26 ppm of the lactone nobilamide I (**8**) (Figures S75, S77, S48 and S50) clearly indicated that **18** is the linear form of nobilamide I (**8**). Nobilamide S (**18**) constitutes a so far unknown linear nobilamide.

Nobilamide T (**19**) (Figures S81–S83) is the linear form of the lactone nobilamide P (**15**), as can be inferred from their related MS/MS fragmentation (Figure S82). Nobilamide T (**19**) was only obtained in minute amounts (<0.2 mg/L). The stereochemistry of the amino acids of **19** was assigned based on the detected amino acids from Marfey’s analysis and the assumption that the stereochemistry of the amino acids in the peptide sequence is analogous to that of A-3302-B (**1**) (Figure S83).

Nobilamide U (**20**) and nobilamide V (**21**) (Figures S84–S86) are linear *N*-acetylheptapeptides containing L-threonine at position seven as determined from their MS/MS fragmentation (Figure S85) and by Marfey’s analysis of the amino acids of the hydrolyzed peptide (Figure S86). However, the presence of a small but significant amount of L-isoleucine in the Marfey-derivatized samples of **20** and **21** (Figure S86) suggests that nobilamide U (**20**) and nobilamide V (**21**) may in addition contain an unidentified isomer.

The pentapeptide **22** (Figures S87–S95) contained, in contrast to the rest of the *N*-acetylheptapeptide nobilamides, two amino acid units less (Figure 8). HR-ESI-MS, MS/MS, NMR, and Marfey’s analysis confirmed that **22** is a linear *N*-acetylpentapeptide with the same amino acid sequence common to the major nobilamides such as A-3302-B (**1**). Nobilamide W (**22**) has not been previously reported.

### 3.7. Screening Nobilamides for Their Bioactivity

The antibiotic potential of selected nobilamides from *Bacillus* sp. G2112, namely A-3302-B (**1**), nobilamide A (**4**), nobilamide I (**8**), nobilamide O (**14**) and nobilamide P (**15**) mixture, nobilamide S (**18**), nobilamide U (**20**) and nobilamide V (**21**) mixture, and nobilamide W (**22**) was initially investigated in agar diffusion assays at 7 µg, 50 µg, and 100 µg/hole against selected phytopathogens (*F. equiseti*, *F. graminearum*, *E. tracheiphila*, and *P. syringae* pv. *glycinea*). In addition, nobilamides were tested to determine whether they inhibit the growth of non-phytopathogenic strains (*Pseudomonas* sp. G124, *B. subtilis*, *B. amyloliquefaciens*, *B. megaterium*, *B. thuringensis*, and *L. sphaericus*)*. Bacillus* strains were included in the screening for antibiotic activity of nobilamides in order to evaluate whether bacilli that do not produce nobilamides are inhibited by them. Among the tested microorganisms, only *L. sphaericus* was inhibited at 7 µg/hole by A-3302-B (**1**) but not by the other tested nobilamides (Figure S96). Testing of nobilamides against *S. aureus*, *S. pyogenes*, and *E. faecalis* was carried out to confirm the antimicrobial activity of compound **1** as previously reported [27,42] and to determine whether other nobilamides exert similar activity as **1**. The testing for antimicrobial activity was extended to other Gram-positive human pathogens, including *K. pneumonia*, *V. cholera*, and the model organism for pathogenic mycobacteria, *M. aurum* [43]. Only A-3302-B (**1**) strongly inhibited the growth of *S. aureus* at as low as 7 µg/hole (Figure S96) but not the growth of *S. pyogenes*, *E. faecalis*, *K. pneumoniae*, *V. cholera,* and *M. aurum* at up to 100 µg/hole. 

Because the antibiotic activity of nobilamides against *L. sphaericus* has not been previously evaluated, further testing of nobilamides was carried out against this organism. The initial tests indicated that A-3302-B (**1**) strongly inhibited *L. sphaericus* at 7 µg/hole in agar diffusion assays. Further testing was performed using concentrations ranging from 0.05 µg/hole to 100 µg/hole. Interestingly, inhibition was only obtained in the range of 0.5 µg to 20 µg/hole, and maximum inhibition of *L. sphaericus* was observed at 5 µg/hole, with an inhibition zone of 21.3 mm ± 0.6 mm (Table S1, Figure S97a). At 0.05 µg/hole, 50 µg/hole, and 100 µg/hole, *L. sphaericus* was not inhibited by A-3302-B (**1**). Repeated experiments yielded the same results. Moreover, A-3302-A (**2**) and nobilamide J (**9**), which differ from A-3302-B (**1**) by just an ***N***-propionyl and ***N***-isobutanoyl moiety, respectively, instead of the ***N***-acetyl moiety of **1**, exerted similar activity as A-3302-B (**1**) against *L. sphaericus* (Figure S97b).

**Figure 9:**
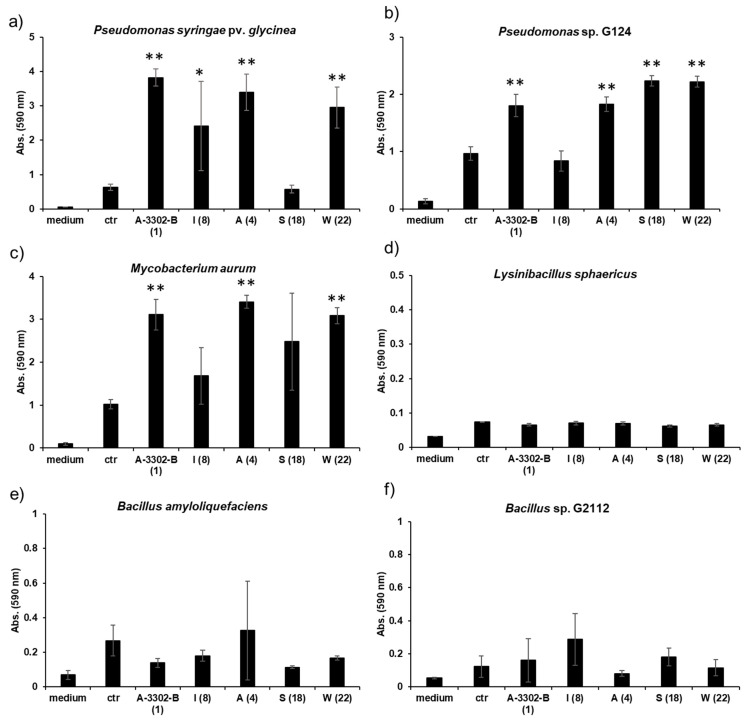
Effect of nobilamides on biofilm formation by pseudomonads, bacilli, and *M. aurum*. A-3302-B (**1**), nobilamide I (**8**), nobilamide A (**4**), nobilamide S (**18**), and nobilamide W (**22**) were tested to determine whether they influence biofilm formation in selected microorganisms. Most tested nobilamides strongly promoted biofilm formation in (**a**) *P. syringae* pv. *glycinea*, (**b**) *Pseudomonas* sp. G124 (isolate from cucumber), and (**c**) *M. aurum*. The selected nobilamides did not cause biofilm formation in (**d**) *L. sphaericus*, (**e**) *B. amyloliquefaciens,* and (**f**) *Bacillus* sp. G2112 that produced the compounds. Legend: medium: King’s B medium, ctr: control: cultures without addition of nobilamide peptides, tested nobilamides are given with their letter identifier and the compound number in brackets. * Denotes significant effect (*p* < 0.05) and ** denotes highly significant effect (*p* < 0.01). Values represent means of three replicates ± standard deviation (error bars).

The potential of nobilamides to influence biofilm formation in other microorganisms was investigated using the crystal violet biofilm staining method [39]. Nobilamide peptides differing in their amino acid length, lactones, and linear nobilamide peptides were selected in order to investigate their effect on biofilm formation (Figure 9, Table S2). Quantities of 0.2 µg/µL of A-3302-B (**1**), nobilamide A (**4**), and nobilamide W (**22**) strongly induced biofilm formation in *M. aurum*, *P. syringae* pv. *glycinea,* and *Pseudomonas* sp. G124 (isolate from cucumber). Nobilamide S (**18**) induced biofilm formation in *Pseudomonas* sp. G124 but did not promote biofilm formation in *P. syringae* pv. *glycinea*. Nobilamide I (**8**) generally had a less clear effect on biofilm formation and worked best on *P. syringae* pv. *glycinea.* In *M. aurum* and *P. syringae* pv. *glycinea*, the increase in biofilm formation caused by A-3302-B (**1**), nobilamide A (**4**), and nobilamide W (**22**) was statistically significant (*p* < 0.01) compared to the control (Figure 9a,c). In *Pseudomonas* sp. G124, all tested nobilamides, except nobilamide I (**8**), caused a significant increase in biofilm production (*p* < 0.01) (Figure 9b). The tested nobilamides did not significantly influence the biofilm production of *B. amyloliquefaciens* (Figure 9e) and *L. sphaericus* (Figure 9d). Interestingly, exogenously added nobilamide peptides also did not influence the biofilm production of the nobilamide-producing *Bacillus* sp. G2112 (Figure 9f).

## 4. Discussion

A-3302-B and nobilamide peptides constitute a group of rarely described *N*-acylpeptides. Interestingly, nobilamide peptides have been reported in diverse bacteria [26,28,29,30,31,44]. Nobilamide peptides have been recognized for their medicinal potential as antibiotics against Gram-positive bacteria [27,28], as vallinoid 1 (TRPV-1) receptor suppressors [30], as inhibitors of cancer cell mobility [31], and as inhibitors of cell-to-cell herpes simplex virus transmission [29]. Moreover, A-3302-B (**1**) was found to inhibit biofilm formation in *S. aureus* [28].

Here we identified that *Bacillus* sp. G2112, an isolate from cucumber (*Cucumis sativus*) leaves, produces a remarkable diversity of antimicrobial peptides: surfactins, iturins, and fengycins [11,40,41], and an unprecedentedly large variety of both known and novel nobilamide peptides (Figure 5, Figure 6, Figure 7 and Figure 8). *Bacillus* sp. G2112 forms nobilamide peptides during its exponential growth phase. A-3302-B (**1**) was produced first and constitutes the most abundant member of the nobilamide-type peptides, while the truncated linear *N*-acetyl-pentapeptide nobilamide W (**22**) appeared last, though still during the exponential growth phase (Figure 3b). In comparison to other secondary metabolites produced by *Bacillus* sp. G2112, namely, surfactins, iturins, and fengycins, only surfactins were detected as early as A-3302-B (**1**) in the culture supernatants (Figure 3a), indicating that the nobilamide peptides may play an important role during the exponential growth of *Bacillus* sp. G2112 because of the investment of energy into their production in the early growth phase [45].

Of the eighteen nobilamide peptides described in *Bacillus* sp. G2112, only four were previously known. These were identified in *Bacillus* sp. TL-119 and *Bacillus* sp. URID 12.1 (A-3302-B (**1**) and A-3302-A (**2**)) [26,27,28], *Streptomyces* sp. CN48 and *Streptomyces* sp. CT3a (A-3302-B (**1**), A-3302-A (**2**), and nobilamide A (**4**)) [30], *Saccharomonospora* sp. CNQ-490 (A-3302-B (**1**) and nobilamide I (**8**)) [31] and *Micromonospora* sp. MAG 9–7 (A-3302-B (**1**)) [29]. The production of the major nobilamide A-3302-B (**1**) by these distantly related microorganisms from different environments but not by related *Bacillus* spp. (Figure S5) suggests that the suspected non-ribosomal biosynthetic gene cluster may have been horizontally exchanged [46,47], although the possibility of convergent evolution cannot be ruled out at the moment [48,49]. *Bacillus* sp. G2112 produces the widest variety of nobilamide peptides known to date. Unlike *Streptomyces* sp. CN48 and *Streptomyces* sp. CT3a, which only produced nobilamide peptides with one phenylalanine to tyrosine substitution and truncated products [30], *Bacillus* sp. G2112′s synthetic machinery appears to have relaxed substrate specificity, permitting amino acid substitutions in five of the seven positions in the heptapeptide sequence (Figure 5, Figure 6, Figure 7 and Figure 8). Some of the nobilamide peptides can reach up to ca. 65% of the main product A-3302-B (**1**). However, we did not find cyclic truncated products, such as nobilamide D (**7**), in which the second amino acid, D-leucine, is left out, which was observed in *Streptomyces* sp. CN48 and *Streptomyces* sp. CT3a, and represents another remarkable flexibility in the biosynthesis of nobilamides [30].

In *Bacillus* sp. G2112, amino acid usage at the sixth position of the nobilamide peptide sequence appeared to be most flexible, permitting glycine, alanine, homoalanine, and serine to be incorporated (Figure 6). Apparently, the responsible adenylation domain of the NRPS can accept several small amino acids [50,51,52]. In the first, second, and third positions of the nobilamide sequence, phenylalanine, leucine, and, rarely, isoleucine could be incorporated by *Bacillus* sp. G2112, generating further product diversity. The observed flexibility of these suspected NRPS domains would be perfectly in line with the studies by Stachelhaus et al., which demonstrated ambiguous use of these amino acids by such adenylation domains [53]. The seventh amino acid of the nobilamide sequence appears to be conserved for L-threonine which likely undergoes product modification by dehydration to Z-α,β-dehydrobutyrine (Dhb) found in some of the nobilamide peptides [54]. Only the fourth and fifth positions of the nobilamide sequence were conserved in all nobilamides identified from *Bacillus* sp. G2112 with D-allo-threonine and L-valine, respectively, and therefore may be crucial for activity. 

The variation in the acyl group length between two and five carbons added to the diversity of nobilamides produced by *Bacillus* sp. G2112. Potentially, several other nobilamides with longer acyl chains could be produced by *Bacillus* sp. G2112. Therefore, offering specific organic acids to *Bacillus* sp. G2112 in the growth medium may increase the yield of specific nobilamides and could lead to more diverse products [55,56].

The occurrence of truncated nobilamide peptides, such as nobilamide W (**22**) and nobilamide C (**6**), may be attributed to flexibility/imperfections in the enzymatic biosynthesis [30] or may be the result of post-NRPS synthesis modifications. The emergence of nobilamide W (**22**) in the late exponential growth phase, its high concentration in the spent culture medium, and its strong biofilm-promoting activity (Figure 9) may suggest that the modifications are spurred by changing ecological needs. Such shortened but still active compounds may represent an economical option in resource-depleted, aging cultures.

Testing nobilamide peptides from *Bacillus* sp. G2112 for their potential to inhibit selected phytopathogens did not reveal any antibiotic activity. However, A-3302-B (**1**) exhibited strong antibiotic activity against *S. aureus* and *L. sphaericus* in low amounts in agar diffusion assays. Antibiotic activity of A-3302-B against *S. aureus*, known for its severe nosocomial and wound infections [57], has been previously demonstrated [27,28]. However, the antibiotic activity of A-3302-B (**1**) and its acyl homologs, A-3302-A (**2**) and nobilamide J (**9**), against *L. sphaericus* is not only novel but also interesting because of its strong specific activity at a narrow concentration range (0.5 μg to 20 μg/hole). Although an organism of growing importance in agricultural biotechnology [58], *L. sphaericus* has also been implicated in severe sepsis in an infected immunocompetent adult [59] and in bacteremia in children undergoing cancer treatment and bone marrow transplant [60]. Nobilamides may provide an effective alternative to ciprofloxacin and penicillin for treating *L. sphaericus* infections. While our results strongly demonstrate that both the lactone ring and the C-terminal dehydrobutyrine are crucial for the antibiotic activity of nobilamides (Figure S97b), more work is needed to elucidate the mechanism that limits the activity within a narrow concentration range.

Rather than antibiotic activity, all tested nobilamides, whether linear (**4**, **18**), cyclic (**1**, **8**), or truncated (nobilamide W, **22**), strongly promoted biofilm formation (*p* < 0.01) in other organisms, both Gram-negative (*Pseudomonas* spp.) and Gram-positive (*M. aurum,* Figure 9). The biofilm-inducing activity of these structurally diverse nobilamides suggests that the common *N*-acetyl-D-Phe-D-Leu-L-Phe-D-allo-Thr-L-Val-pentapeptide motif may be crucial for the biofilm-inducing effect. In contrast to our observation of biofilm induction in *Pseudomonas* spp. and *M. aurum*, Chalasani et al. observed that A-3302-B (**1**) inhibited biofilm development in *S. aureus* [28], indicating that different microorganisms react differently to nobilamides. Moreover, the tested nobilamides did not obviously influence biofilm formation in their producer *Bacillus* sp. G2112 and other tested *Bacilli* (Figure 9). However, it must be noted that our method for determining biofilms is not suitable for determining every kind of biofilms produced by microorganisms (floating biofilms) [61].

Microbial secondary metabolites play diverse roles beyond killing as antibiotics in their natural contexts [3,6,62,63,64]. Sublethal concentrations of antibiotics that cause potassium leakage, including the self-produced surfactin, have been argued to constitute a signal for the induction of biofilm formation in *B. subtilis* [7]. A different point of view identifies these ‘signals’ as ecological stressors that induce microorganisms to respond in specific ways, including formation of biofilms as protection [65,66,67]. Indeed, different organisms respond differently to the same secondary metabolites [8,68,69]. Therefore, the promotion of biofilm formation by nobilamides in organisms that they do not kill suggests that nobilamides cause physiological stress in those organisms (Figure 9). *Bacillus* sp. G2112 may use nobilamide peptides, among others, to limit competitors such as *Pseudomonas* sp. G124 to defensive biofilm states that reduce the space and resources they can acquire from their host plants. Biofilm induction in foreign microorganisms can have a strong impact on shaping microbial communities, and associated higher organisms such as host plants may benefit [70,71]. Kulkarni et al. nicely demonstrated that methyl jasmonate from plants induces biofilm formation in the rhizosphere and thus is involved in shaping its microbial community [71]. Moreover, cyclic lipopeptides from *Bacillus* spp. and *Pseudomonas* spp. have been shown to influence microbial communities in the rhizosphere [10,11]. A similar scenario can be conceived for *Bacillus* sp. G2112 whereby it would influence the growth physiology of microbial neighbors on plants, such as *Pseudomonas* sp. G124, which was also isolated from cucumber leaves [13] using nobilamides. Because both *Bacillus* spp. and *Pseudomonas* spp. are common in the rhizosphere, one may speculate that *Bacillus* sp. G2112 may also occur in the cucumber rhizosphere and produce nobilamides that may influence the rhizosphere microbiome of (cucumber) plants and thus cause beneficial effects for crop plants. Clearly, it is important to uncover the biological functions of secondary metabolites, such as nobilamides, in their natural contexts beyond antibiotic activity. Doing so may reveal novel applications for these compounds, e.g., in biocontrol. 

Future experiments should address in detail the ecological role of nobilamides for the producing strain *Bacillus* sp. G2112 in order to understand what advantages *Bacillus* sp. G2112, and the host plant, might gain from inducing biofilm formation in other neighboring organisms such as pseudomonads.

## Data Availability

The data presented in this study are available in the Supplementary Materials and from the authors.

## Supplementary Materials

The supporting information can be downloaded at: https://www.mdpi.com/article/10.3390/biom14101244/s1. Figure S1: Global natural products social (GNPS) molecular networking analysis of the secondary metabolites extracted with ethyl acetate at pH 2.5 from the culture supernatant of *Bacillus* sp. G2112 grown in King’s B medium for 14 d; Figure S2: Growth curve of *Bacillus* sp. G2112 grown in King’s B medium for 4 d; Figure S3: Comparison of the timing of peptide secondary metabolite formation by *Bacillus* sp. G2112; Figure S4: Time-course of the production of major nobilamide peptides; Figure S5: Screening *Bacillus* spp. for nobilamide production by LC-MS; Figure S6: HR-ESI-MS of A-3302-B (**1**); Figure S7: MS/MS identification of A-3302-B (**1**); Figure S8: Stereochemistry of the amino acids of A-3302-B (**1**); Figure S9: ^1^H NMR spectrum (800 MHz, CD_3_OD) of A-3302-B (**1**); Figure S10: ^13^C NMR spectrum (201 MHz, CD_3_OD) of A-3302-B (**1**); Figure S11: ^1^H-^1^H-COSY NMR spectrum (800 MHz, CD_3_OD) of A-3302-B (**1**); Figure S12: ^1^H-^13^C-HSQC NMR spectrum (800 MHz, CD_3_OD) of A-3302-B (**1**); Figure S13: ^1^H-^13^C HMBC NMR spectrum (800 MHz, CD_3_OD) of A-3302-B (**1**); Figure S14: Key ^1^H-^1^H COSY and ^1^H-^13^C HMBC correlations of A-3302-B (**1**); Figure S15: HR-ESI-MS of A-3302-A (**2**); Figure S16: MS/MS of the quasimolecular ion [M+H]^+^ *m*/*z* 818.4 of A-3302-A (**2**); Figure S17: Stereochemistry of the amino acids of A-3302-A (**2**); Figure S18: HR-ESI-MS of nobilamide J (**9**); Figure S19: ESI-MS/MS of nobilamide J (**9**); Figure S20: Stereochemistry of the amino acids of nobilamide J (**9**); Figure S21: ^1^H NMR spectrum (600 MHz, CD_3_OD) of nobilamide J (**9**); Figure S22: ^13^C NMR spectrum (151 MHz, CD_3_OD) of nobilamide J (**9**); Figure S23: ^1^H-^1^H COSY NMR spectrum (600 MHz, CD_3_OD) of nobilamide J (**9**); Figure S24: ^1^H-^13^C HSQC NMR spectrum (600 MHz, CD_3_OD) of nobilamide J (**9**); Figure S25: ^1^H-^13^C-HMBC NMR spectrum (600 MHz, CD_3_OD) of nobilamide J (**9**); Figure S26: Key ^1^H-^1^H COSY and ^1^H-^13^C HMBC correlations of nobilamide J (**9**); Figure S27: HR-ESI-MS of nobilamide K (**10**); Figure S28: ESI-MS/MS of nobilamide K (**10**); Figure S29: Stereochemistry of the amino acids of nobilamide K (**10**); Figure S30: HR-ESI-MS of nobilamide L (**11**); Figure S31: ESI-MS/MS of nobilamide L (**11**); Figure S32: Stereochemistry of the amino acids of nobilamide L (**11**); Figure S33: ^1^H NMR spectrum (600 MHz, CD_3_OD) of nobilamide L (**11**); Figure S34: ^13^C NMR spectrum (151 MHz, CD_3_OD) of nobilamide L (**11**); Figure S35: ^1^H-^1^H COSY NMR spectrum (600 MHz, CD_3_OD) of nobilamide L (**11**); Figure S36: ^1^H-^13^C HSQC NMR spectrum (600 MHz, CD_3_OD) of nobilamide L (**11**); Figure S37: ^1^H-^13^C HMBC NMR spectrum (600 MHz, CD_3_OD) of nobilamide L (**11**); Figure S38: Key ^1^H-^1^H COSY and ^1^H-^13^C HMBC NMR spectrum (600 MHz, CD_3_OD) of nobilamide L (**11**); Figure S39: HR-ESI-MS of nobilamide M (**12**); Figure S40: ESI-MS/MS of nobilamide M (**12**); Figure S41: Stereochemistry of the amino acids of nobilamide M (**12**); Figure S42: HR-ESI-MS of nobilamide N (**13**); Figure S43: ESI-MS/MS of nobilamide N (**13**); Figure S44: Stereochemistry of the amino acids of nobilamide N (**13**); Figure S45: HR-ESI-MS of nobilamide I (**8**); Figure S46: ESI-MS/MS of nobilamide I (**8**); Figure S47: Stereochemistry of the amino acids of nobilamide I (**8**); Figure S48: ^1^H NMR spectrum (600 MHz, CD_3_OD) of nobilamide I (**8**); Figure S49: ^13^C NMR spectrum (151 MHz, CD_3_OD) of nobilamide I (**8**); Figure S50: ^1^H-^1^H COSY NMR spectrum (600 MHz, CD_3_OD) of nobilamide I (**8**); Figure S51: ^1^H-^13^C HSQC NMR spectrum (600 MHz, CD_3_OD) of nobilamide I (**8**); Figure S52: ^1^H-^13^C HMBC NMR spectrum (600 MHz, CD_3_OD) of nobilamide I (**8**); Figure S53: Key ^1^H-^1^H COSY and ^1^H-^13^C HMBC NMR spectrum (600 MHz, CD_3_OD) of nobilamide I (**8**); Figure S54: ESI-MS/MS of nobilamide O (**14,** below) and nobilamide P (**15,** above); Figure S55: HR-ESI-MS of nobilamide O (**9**) and nobilamide P (**10**); Figure S56: Stereochemistry of the amino acids of the mixture of nobilamide O (**14**) and nobilamide P (**15**); Figure S57: HR-ESI-MS of nobilamide Q (**16**); Figure S58: ESI-MS/MS of nobilamide Q (**16**); Figure S59: Stereochemistry of the amino acids of nobilamide Q (**16**); Figure S60: HR-ESI-MS of nobilamide R (**17**); Figure S61: ESI-MS/MS of nobilamide R (**17**); Figure S62: Stereochemistry of the amino acids of nobilamide R (**17**); Figure S63: HR-ESI-MS of nobilamide A (**4**); Figure S64: ESI-MS/MS of nobilamide A (**4**); Figure S65: Stereochemistry of the amino acids of nobilamide A (**4**); Figure S66: ^1^H NMR spectrum (600 MHz, CD_3_OD) of nobilamide A (**4**); Figure S67: ^13^C NMR spectrum (151 MHz, CD_3_OD) of nobilamide A (**4**); Figure S68: ^1^H-^1^H COSY NMR spectrum (600 MHz, CD_3_OD) of nobilamide A (**4**); Figure S69: ^1^H-^13^C HSQC NMR spectrum (600 MHz, CD_3_OD) of nobilamide A (**4**); Figure S70: ^1^H-^13^C HMBC NMR spectrum (600 MHz, CD_3_OD) of nobilamide A (**4**); Figure S71: Key ^1^H-^1^H COSY and ^1^H-^13^C HMBC NMR spectrum (600 MHz, CD_3_OD) of nobilamide A (**4**); Figure S72: HR-ESI-MS of nobilamide S (**18**); Figure S73: ESI-MS/MS of nobilamide S (**18**); Figure S74: Stereochemistry of the amino acids of nobilamide S (**18**); Figure S75: ^1^H NMR spectrum (600 MHz, CD_3_OD) of nobilamide S (**18**); Figure S76: ^13^C NMR spectrum (151 MHz, CD_3_OD) of nobilamide S (**18**); Figure S77: ^1^H-^1^H COSY NMR spectrum (600 MHz, CD_3_OD) of nobilamide S (**18**); Figure S78: ^1^H-^13^C HSQC NMR spectrum (600 MHz, CD_3_OD) of nobilamide S (**18**); Figure S79: ^1^H-^13^C HMBC NMR spectrum (600 MHz, CD_3_OD) of nobilamide S (**18**); Figure S80: Key ^1^H-^1^H COSY and ^1^H-^13^C HMBC NMR spectrum (600 MHz, CD_3_OD) of nobilamide S (**18**); Figure S81: HR-ESI-MS of nobilamide T (**19**); Figure S82: ESI-MS/MS of nobilamide T (**19**); Figure S83: Stereochemistry of the amino acids of nobilamide T (**19**); Figure S84: HR-ESI-MS of nobilamide U (**20**) and nobilamide V (**21**); Figure S85: ESI-MS/MS of nobilamide U (**20**) and nobilamide V (**21**); Figure S86: Stereochemistry of the amino acids the mixture of nobilamide U (**20**) and nobilamide V (**21**); Figure S87: HR-ESI-MS of nobilamide W (**22**); Figure S88: ESI-MS/MS of nobilamide W (**22**); Figure S89: Stereochemistry of the amino acids of nobilamide W (**22**); Figure S90: ^1^H NMR spectrum (600 MHz, CD_3_OD) of nobilamide W (**22)**; Figure S91: ^13^C NMR spectrum (151 MHz, CD_3_OD) of nobilamide W (**22**); Figure S92: ^1^H-^1^H COSY NMR spectrum (600 MHz, CD_3_OD) of nobilamide W (**22**); Figure S93: ^1^H-^13^C HSQC NMR spectrum (600 MHz, CD_3_OD) of nobilamide W (**22**); Figure S94: ^1^H-^13^C HMBC NMR spectrum (600 MHz, CD_3_OD) of nobilamide W (**22**); Figure S95: Key ^1^H-^1^H COSY and ^1^H-^13^C HMBC NMR spectrum (600 MHz, CD_3_OD) of nobilamide W (**22**); Figure S96: Agar diffusion assays of nobilamide peptides against microbial pathogens; Figure S97: Pictures of agar diffusion assays of nobilamide peptides against *L. sphaericus* in different amounts/hole; Table S1: Diameter of inhibition zones caused by A-3302-B (**1**) against *L. sphaericus* in agar diffusion assays; Table S2: Means and standard deviation values for the biofilm assays.
