# Supplementary material for: The Epiphyte Bacillus sp. G2112 Produces a Large Diversity of Nobilamide Peptides That Promote Biofilm Formation in Pseudomonads and Mycobacterium aurum
Source: Biomolecules. 2024 Oct 1;14(10):1244. doi: 10.3390/biom14101244 (PMC11505918; doi:10.3390/biom14101244)
Supplement: Supplementary file 1 [file biomolecules-14-01244-s001.zip › biomolecules-3167481-supplementary.pdf]

The Epiphyte *Bacillus* sp. G2112 Produces a Large Diversity of Nobilamide Peptides That Promote Biofilm Formation in Pseudomonads and *Mycobacterium aurum*

Kenechukwu Iloabuchi[1,2] and Dieter Spiteller[1*]

[1]Chemical Ecology/Biological Chemistry, Department of Biology, University of Konstanz, Universitätsstraße 10, 78457 Konstanz, Germany
[2]Department of Biochemistry, Faculty of Biological Sciences, University of Nigeria Nsukka, Obukpa Road, 410105 Nsukka, Nigeria

Table of contents

Molecular networking analysis of secondary metabolites from *Bacillus* sp. G2112 5
Growth curve of *Bacillus* sp. G2112 6
Comparison of the timing of peptide secondary metabolite formation by *Bacillus* sp. G2112.. 7
Time course of the formation of nobilamide peptides 8
Screening *Bacillus* spp. for nobilamide production 9
A-3302-B (1) 10
  HR-ESI-MS of A-3302-B (1) 10
  ESI-MS/MS of A-3302-B (1) 10
  Stereochemistry of the amino acids of A-3302-B (1) 11
  ^{1}H NMR spectrum of A-3302-B (1) 12
  ^{13}C NMR spectrum of A-3302-B (1) 12
  ^{1}H-^{1}H-COSY NMR spectrum of A-3302-B (1) 13
  ^{1}H-^{13}C-HSQC NMR spectrum of A-3302-B (1) 14
  ^{1}H-^{13}C HMBC NMR spectrum of A-3302-B (1) 15
  Key ^{1}H-^{1}H COSY and ^{1}H-^{13}C HMBC correlations of A-3302-B (1) 16
NMR data of A-3302-B (1) 16
A-3302-A (2) 17
  HR-ESI-MS of A-3302-A (2) 17
  HR-MS/MS of A-3302-A (2) 17
  Stereochemistry of the amino acids of A-3302-A (2) 18
Nobilamide J (9) 19
  HR-ESI-MS of nobilamide J (9) 19
  ESI-MS/MS of nobilamide J (9) 19

Stereochemistry of the amino acids of nobilamide J (9) 20
^{1}H NMR spectrum of nobilamide J (9) 21
^{13}C NMR spectrum of nobilamide J (9) 21
^{1}H-^{1}H COSY NMR spectrum of nobilamide J (9) 22
^{1}H-^{13}C HSQC NMR spectrum of nobilamide J (9) 23
HMBC NMR spectrum of nobilamide J (9) 24
Key ^{1}H-^{1}H COSY and ^{1}H-^{13}C HMBC correlations of nobilamide J (9) 25
NMR data of nobilamide J (9) 25
Nobilamide K (10) 26
HR-ESI-MS of nobilamide K (10) 26
ESI-MS/MS of nobilamide K (10) 26
Stereochemistry of the amino acids of nobilamide K (10) 27
Nobilamide L (11) 28
HR-ESI-MS of nobilamide L (11) 28
ESI-MS/MS of nobilamide L (11) 28
Stereochemistry of the amino acids of nobilamide L (11) 29
^{1}H NMR spectrum of nobilamide L (11) 30
^{13}C NMR spectrum of nobilamide L (11) 30
^{1}H-^{1}H COSY NMR spectrum of nobilamide L (11) 31
^{1}H-^{13}C HSQC NMR spectrum (600 MHz, CD_3OD) of nobilamide L (11) 32
^{1}H-^{13}C HMBC NMR spectrum of nobilamide L (11) 33
Key ^{1}H-^{1}H COSY and ^{1}H-^{13}C HMBC NMR spectrum of nobilamide L (11) 34
NMR data of nobilamide L (11) 34
Nobilamide M (12) 35
HR-ESI-MS of nobilamide M (12) 35
ESI-MS/MS of nobilamide M (12) 35
Stereochemistry of the amino acids of nobilamide M (12). 36
Nobilamide N (13) 37
HR-ESI-MS of nobilamide N (13) 37
ESI-MS/MS of nobilamide N (13) 37
Stereochemistry of the amino acids of nobilamide N (13) 38
Nobilamide I (8) 39
HR-ESI-MS of nobilamide I (8) 39
ESI-MS/MS of nobilamide I (8) 39

Stereochemistry of the amino acids of nobilamide I (8) 40
^{1}H NMR spectrum of nobilamide I (8) 41
^{13}C NMR spectrum of nobilamide I (8) 41
^{1}H-^{1}H COSY NMR spectrum of nobilamide I (8) 42
^{1}H-^{13}C HSQC NMR spectrum (600 MHz, CD_3OD) of nobilamide I (8) 43
^{1}H-^{13}C HMBC NMR spectrum (600 MHz, CD_3OD) of nobilamide I (8) 44
Key ^{1}H-^{1}H COSY and ^{1}H-^{13}C HMBC NMR spectrum of nobilamide I (8) 45
NMR data of nobilamide I (8) 45
Nobilamide O (14) and Nobilamide P (15) 46
HR-ESI-MS of nobilamide O (14) and nobilamide P (15) 46
ESI-MS/MS of nobilamide O (14) and nobilamide P (15) 46
Stereochemistry of the amino acids of the mixture of nobilamide O (14) and nobilamide P (15) 47
Nobilamide Q (16) 48
HR-ESI-MS of nobilamide Q (16)) 48
ESI-MS/MS of nobilamide Q (16) 48
Stereochemistry of the amino acids of nobilamide Q (16) 49
HR-ESI-MS of nobilamide R (17) 50
ESI-MS/MS of nobilamide R (17) 50
Stereochemistry of the amino acids of nobilamide R (17) 51
Nobilamide A (4) 52
HR-ESI-MS of nobilamide A (4) 52
ESI-MS/MS of nobilamide A (4) 52
Stereochemistry of the amino acids of nobilamide A (4) 53
^{1}H NMR spectrum (600 MHz, CD_3OD) of nobilamide A (4) 53
^{13}C NMR spectrum (151 MHz, CD_3OD) of nobilamide A (4) 54
^{1}H-^{1}H COSY NMR spectrum (600 MHz, CD_3OD) of nobilamide A (4) 55
^{1}H-^{13}C HSQC NMR spectrum of nobilamide A (4) 56
^{1}H-^{13}C HMBC NMR spectrum of nobilamide A (4) 57
Key ^{1}H-^{1}H COSY and ^{1}H-^{13}C HMBC NMR spectrum of nobilamide A (4) 58
NMR data of nobilamide A (4) 58
Nobilamide S (18) 59
HR-ESI-MS of nobilamide S (18) 59
ESI-MS/MS of nobilamide S (18) 59

Stereochemistry of the amino acids of nobilamide S (18) 60
^{1}H NMR spectrum of nobilamide S (18) 61
^{13}C NMR spectrum of nobilamide S (18) 61
^{1}H-^{1}H COSY NMR spectrum of nobilamide S (18) 62
^{1}H-^{13}C HSQC NMR spectrum (600 MHz, CD_3OD) of nobilamide S (18) 63
^{1}H-^{13}C HMBC NMR spectrum (600 MHz, CD_3OD) of nobilamide S (18) 64
Key ^{1}H-^{1}H COSY and ^{1}H-^{13}C HMBC NMR spectrum of nobilamide S (18) 65
NMR data of nobilamide S (18) 65
Nobilamide T (19) 66
HR-ESI-MS of nobilamide T (19) 66
ESI-MS/MS of nobilamide T (19) 66
Stereochemistry of the amino acids of the mixture of nobilamide T (19) 67
Nobilamide U (20) and nobilamide V (21) 68
HR-ESI-MS of nobilamide U (20) and nobilamide V (21) 68
ESI-MS/MS of nobilamide U (20) and nobilamide V (21) 68
Stereochemistry of the amino acids the mixture of nobilamide U (20) and nobilamide V (21) 69
Nobilamide W (22) 70
HR-ESI-MS of nobilamide W (22) 70
ESI-MS/MS of nobilamide W (22) 70
Stereochemistry of the amino acids of nobilamide W (22) 71
^{1}H NMR spectrum of nobilamide W (22) 72
^{13}C NMR spectrum of nobilamide W (22) 72
^{1}H-^{1}H COSY NMR spectrum of nobilamide W (22) 73
^{1}H-^{13}C HSQC NMR spectrum of nobilamide W (22) 74
^{1}H-^{13}C HMBC NMR spectrum of nobilamide W (22) 75
Key ^{1}H-^{1}H COSY and ^{1}H-^{13}C HMBC NMR spectrum (600 MHz, CD_3OD) of nobilamide W (22) 76
NMR data of nobilamide W (22) 76
Agar diffusion assays of nobilamide peptides against microbial pathogens 77
Agar diffusion assays of nobilamide peptides against *L. sphaericus* 78
Diameter of inhibition zones caused by nobilamides against *L. sphaericus* 79
Effect of nobilamides on biofilm formation of selected microorganisms 80
References 81

Molecular networking analysis of secondary metabolites from *Bacillus* sp. G2112

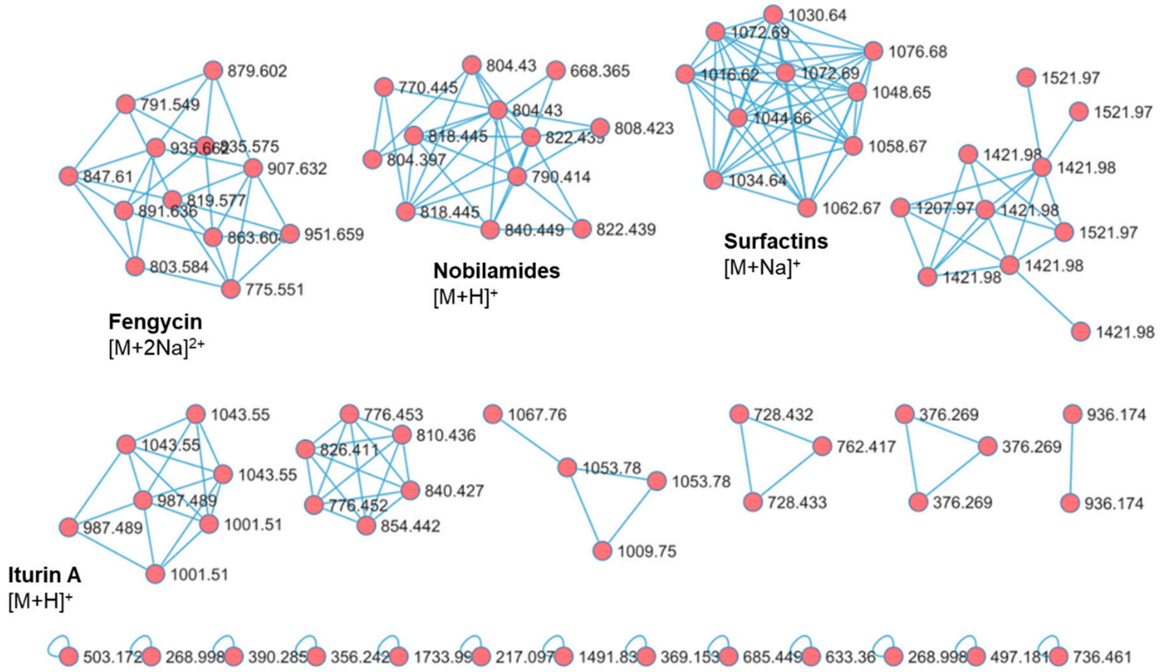

Figure S1: Global natural products social (GNPS) molecular networking analysis [**36**] of the secondary metabolites extracted with ethyl acetate at pH 2.5 from the culture supernatant of *Bacillus* sp. G2112 grown in King's B medium for 14 d. The clusters for surfactin, fengycin, iturin, and nobilamide peptides are indicated. (GNPS; http://gnps.ucsd.edu, accessed on 7th December 2023).

Growth curve of *Bacillus* sp. G2112

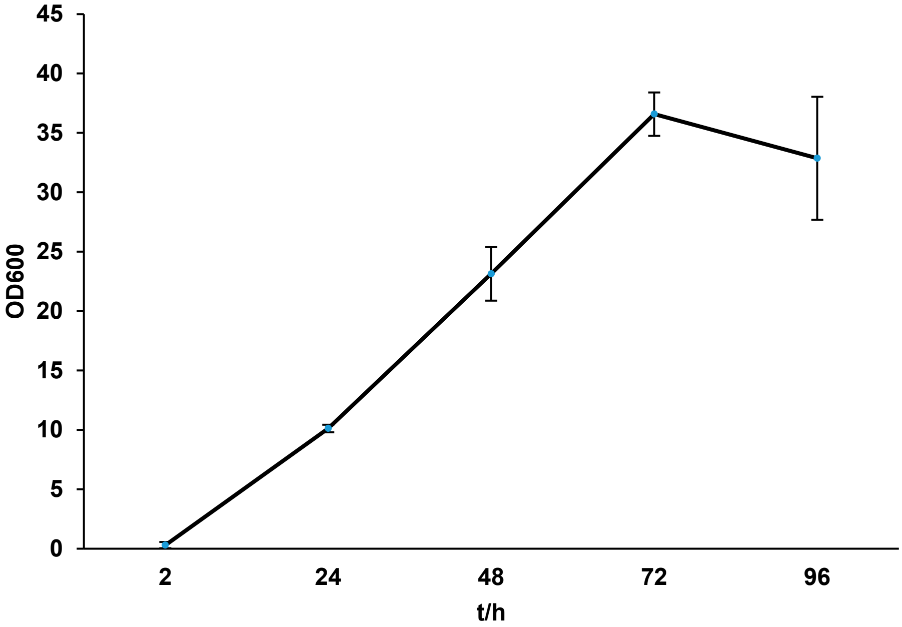

Figure S2: Growth curve of *Bacillus* sp. G2112 grown in King's B medium for 4 d. Optical density was measured at 600 nm in triplicate. Error bars represent standard deviation.

Comparison of the timing of peptide secondary metabolite formation by *Bacillus* sp. G2112

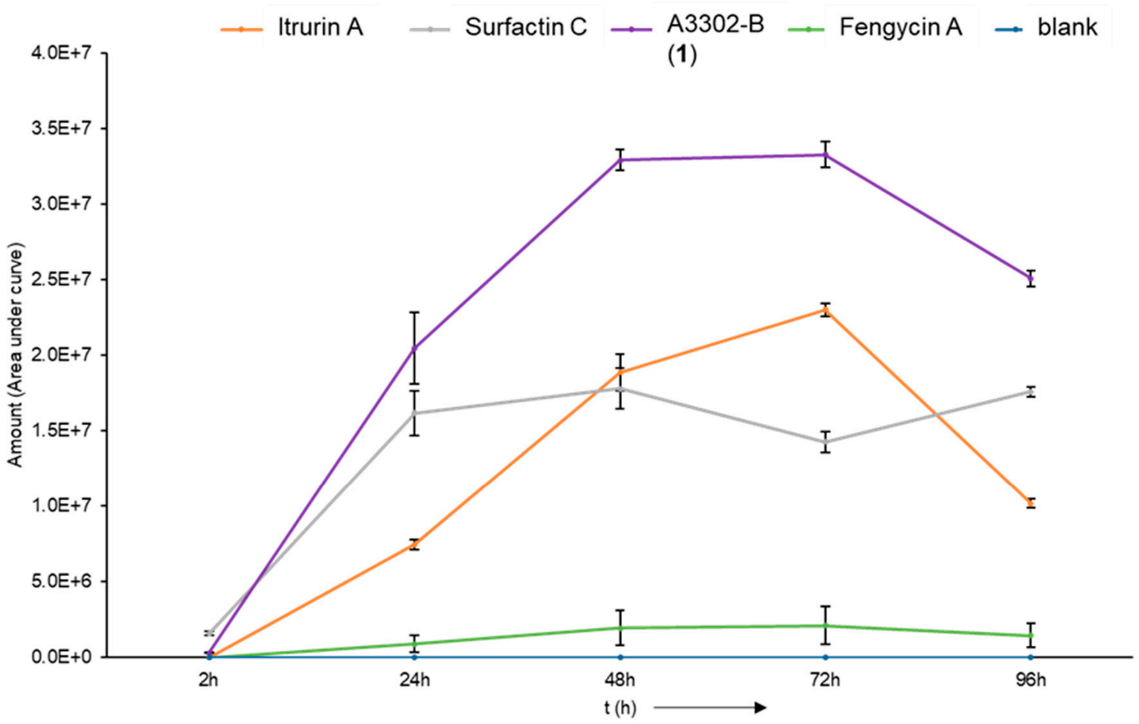

Figure S3: Comparison of the timing of peptide secondary metabolite formation by *Bacillus* sp. G2112: nobilamide A-3302-B (**1**, ([M+H]$^+$ *m/z* 804.4), iturin A ([M+H]$^+$ *m/z* 1057.6), surfactin C15 ([M+H]$^+$ *m/z* 1036.7), and fengycin A ([M+H]$^+$ *m/z* 1463.8). Data points represent mean values of three biological replicates. Error bars indicate standard deviation. Only peak aeras of the [M+H]$^+$ ions are depicted that do not reflect the amounts of the respective compounds but show when the metabolites reach maximal production.

Time course of the formation of nobilamide peptides

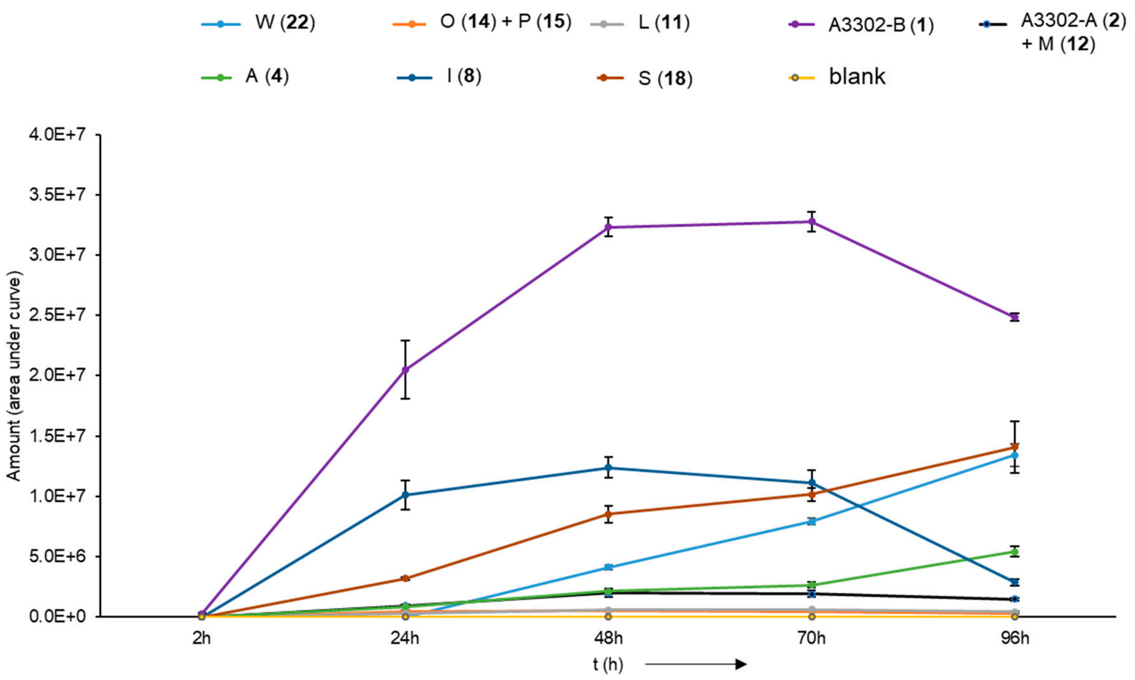

Figure S4: Time-course of the production of major nobilamide peptides. Relative production of A-3302-B (**1**), A-3302-A (**2**), nobilamide I (**8**), nobilamide L (**11**), nobilamide M (**12**), nobilamide O (**14**) and nobilamide P (**15**), nobilamide A (**4**), nobilamide S (**18**) and nobilamide W (**22**) over time was determined from the respective peak areas. Data points represent mean values of three biological replicates. Error bars represent standard deviation.

Screening *Bacillus* spp. for nobilamide production

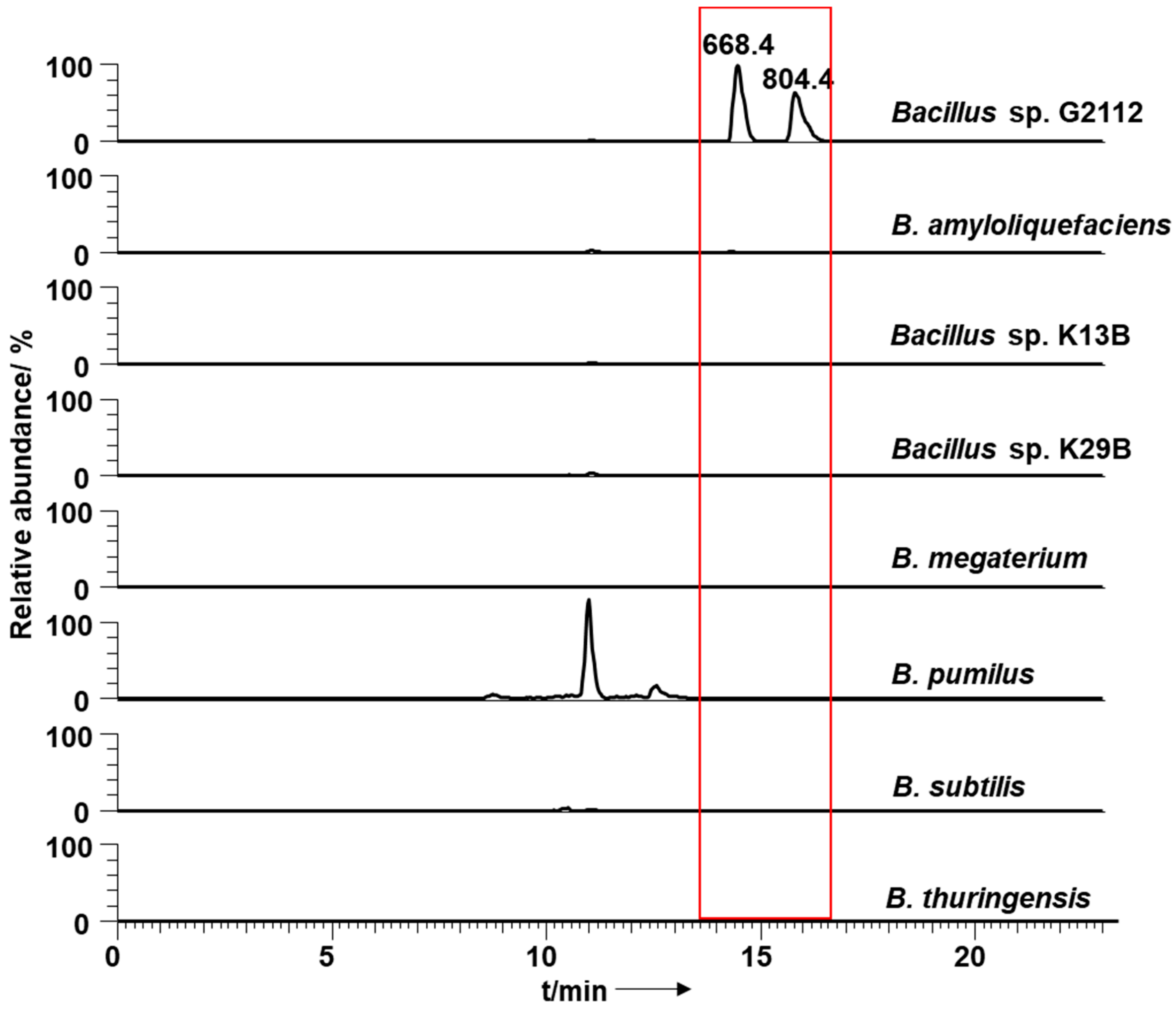

Figure S5. Screening *Bacillus* spp. for nobilamide production by LC-MS. The combined ion trace chromatograms for A-3302-B (**1**, $[M+H]^+$ at *m/z* 804.4) and nobilamide Y (**19**, $[M+H]^+$ at *m/z* 668.4) are depicted. The spent medium supernatants of eight different *Bacillus* strains grown in King's B medium for 14 d were analysed. Only *Bacillus* sp. G2112 produced nobilamide peptides (HPLC method: LC-MS LTQ).

A-3302-B (1)

HR-ESI-MS of A-3302-B (1)

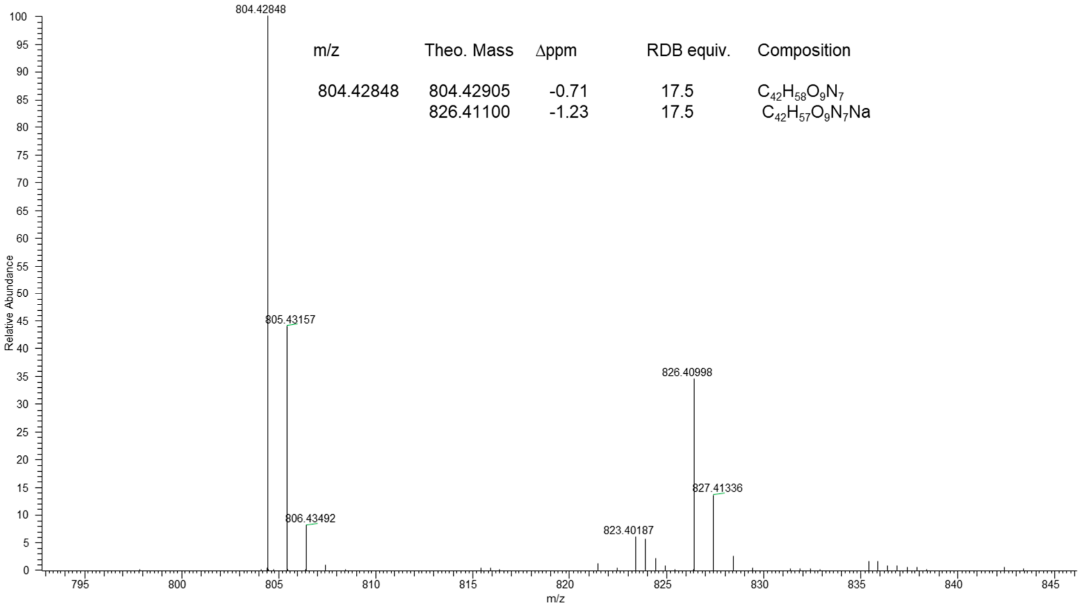

Figure S6. HR-ESI-MS of A-3302-B (**1**), retention time 19.5 min, Macherey-Nagel RP8 column (250 x 2 mm, 5 µm), HPLC method: LC-HR-ESI-MS.

ESI-MS/MS of A-3302-B (1)

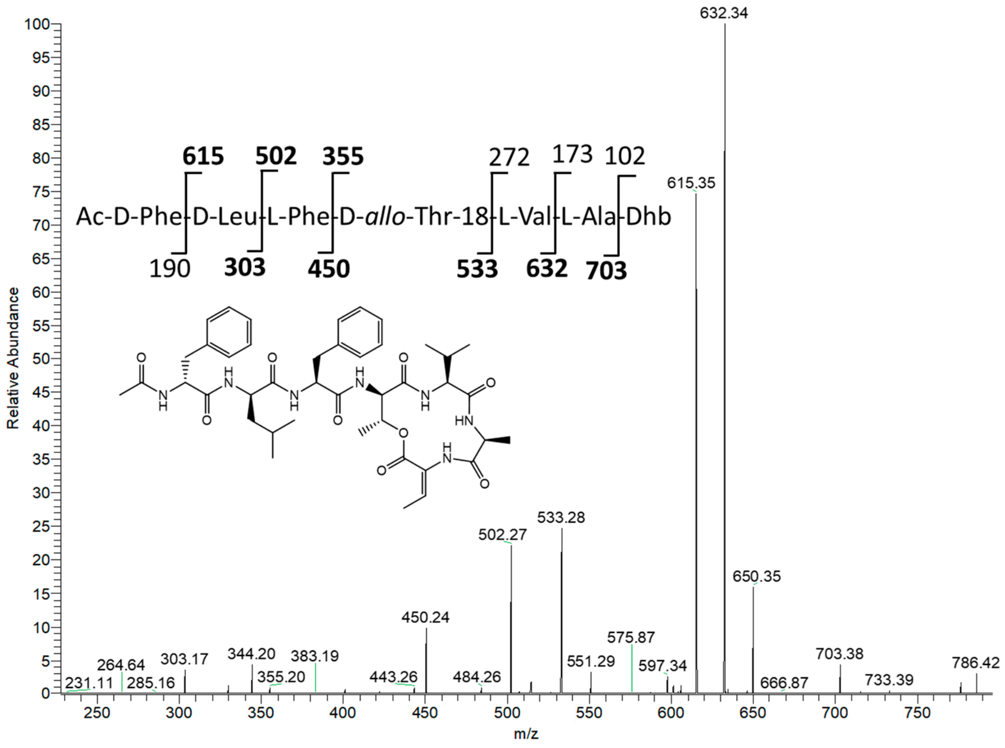

Figure S7. MS/MS identification of A-3302-B (**1**). MS/MS of the quasimolecular ion *m/z* 804.4 of A-3302-B (**1**). The structure of **1** and the y and b ions fragments of **1** after initial ring opening of the ester (allo-Thr-18) are shown. Observed fragments are highlighted in bold. Dhb: *Z*-α,β-dehydrobutyrine.

Stereochemistry of the amino acids of A-3302-B (1)

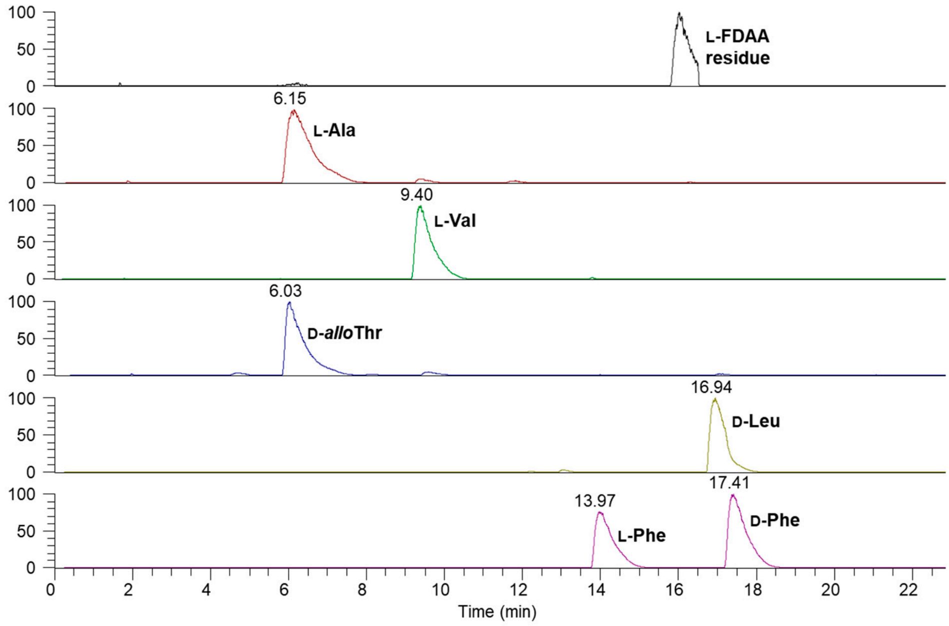

Figure S8: Stereochemistry of the amino acids of A-3302-B (**1**). The stereochemistry of the amino acids of A-3302-B (**1**) was analysed by LC-MS after acid hydrolysis of A-3302-B (**1**) and derivatization with Marfey's reagent **[37,38]**.

^{1}H NMR spectrum of A-3302-B (1)

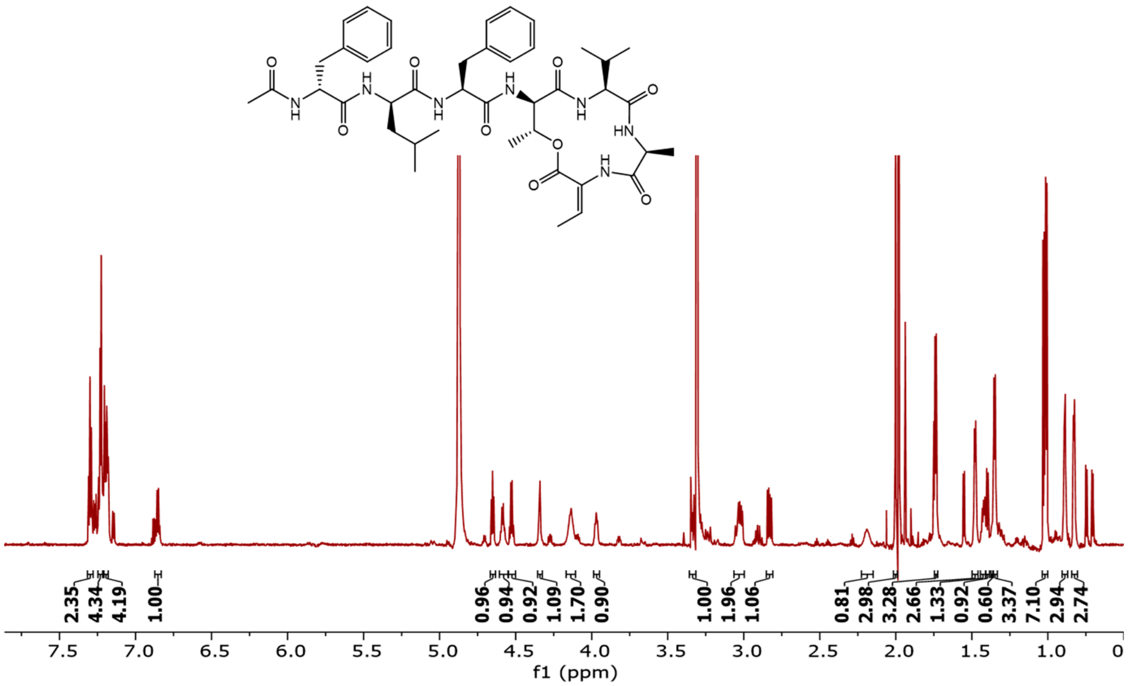

Figure S9. ^{1}H NMR spectrum (800 MHz, CD_3OD) of A-3302-B (**1**)

^{13}C NMR spectrum of A-3302-B (1)

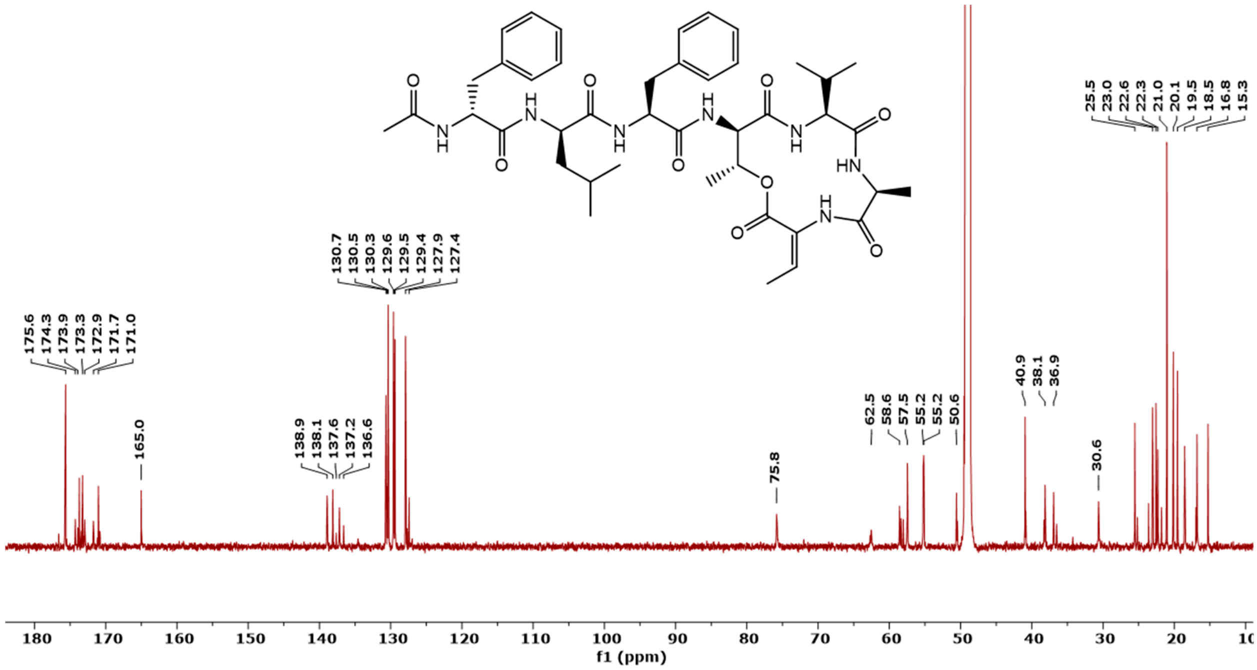

Figure S10. ^{13}C NMR spectrum (201 MHz, CD_3OD) of A-3302-B (**1**).

^{1}H-^{1}H-COSY NMR spectrum of A-3302-B (1)

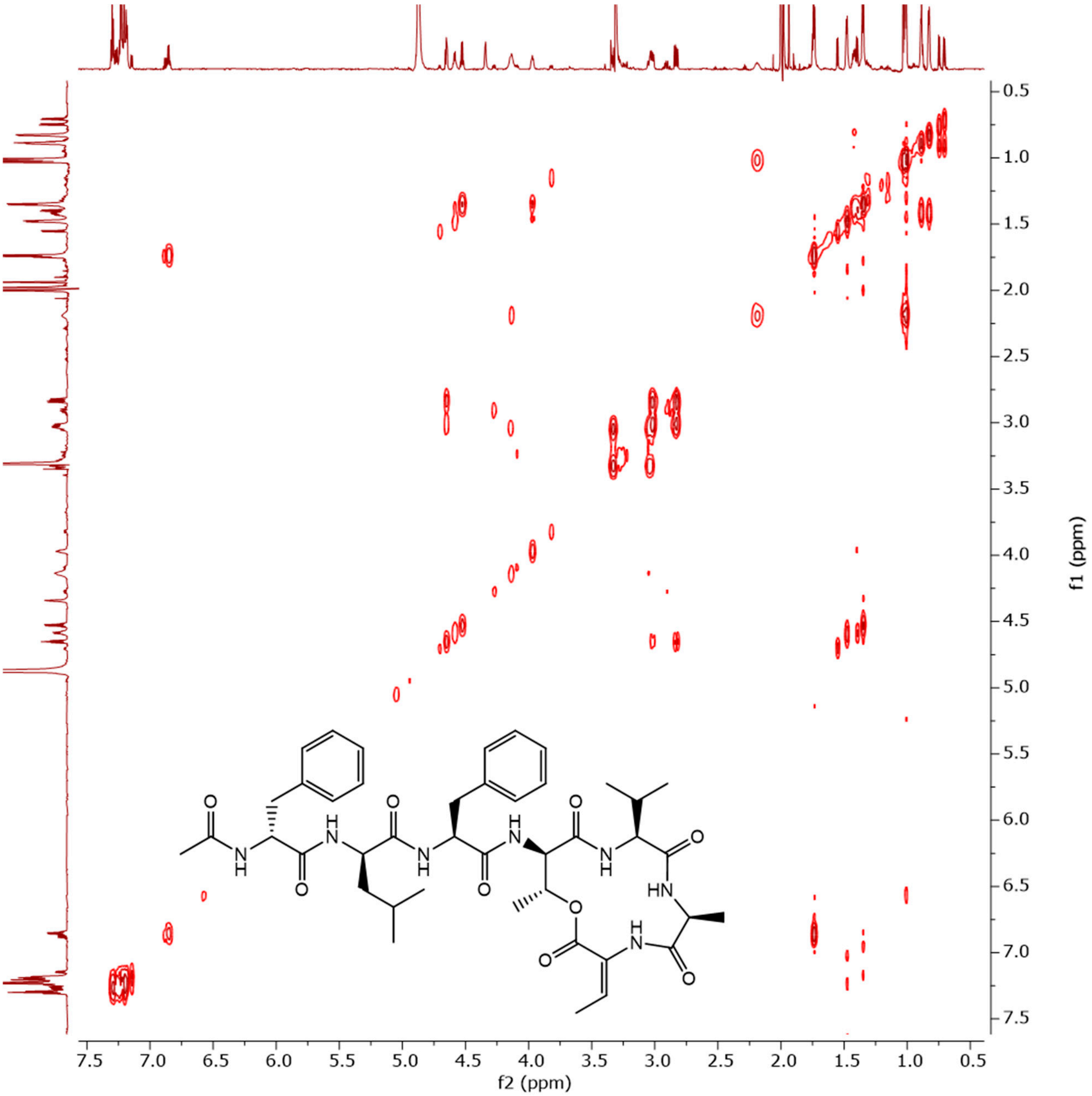

Figure S11. ^{1}H-^{1}H-COSY NMR spectrum (800 MHz, CD_3OD) of A-3302-B (**1**).

^{1}H-^{13}C-HSQC NMR spectrum of A-3302-B (1)

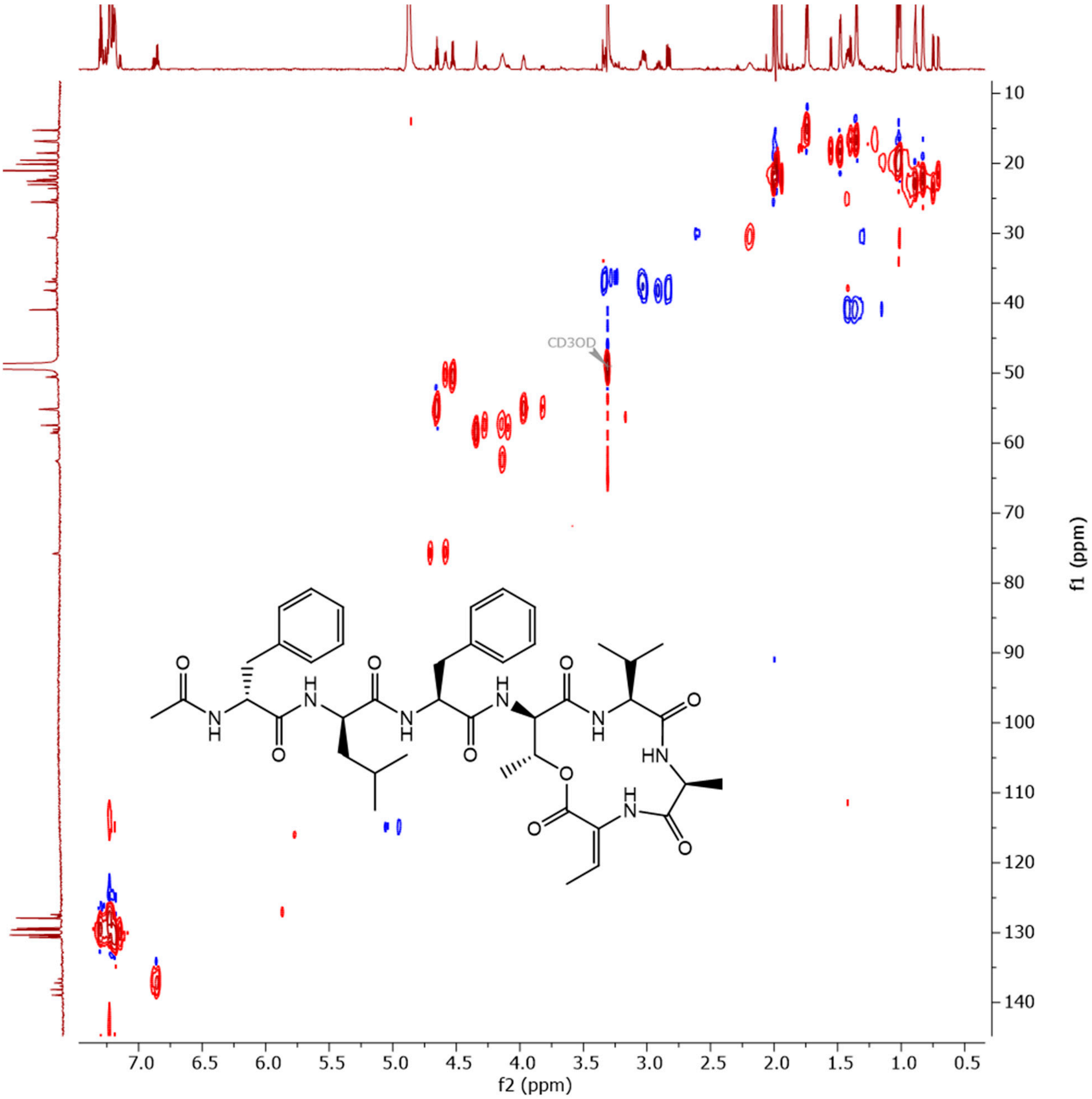

Figure S12. ^{1}H-^{13}C-HSQC NMR spectrum (800 MHz, CD_3OD) of A-3302-B (**1**).

^{1}H-^{13}C HMBC NMR spectrum of A-3302-B (1)

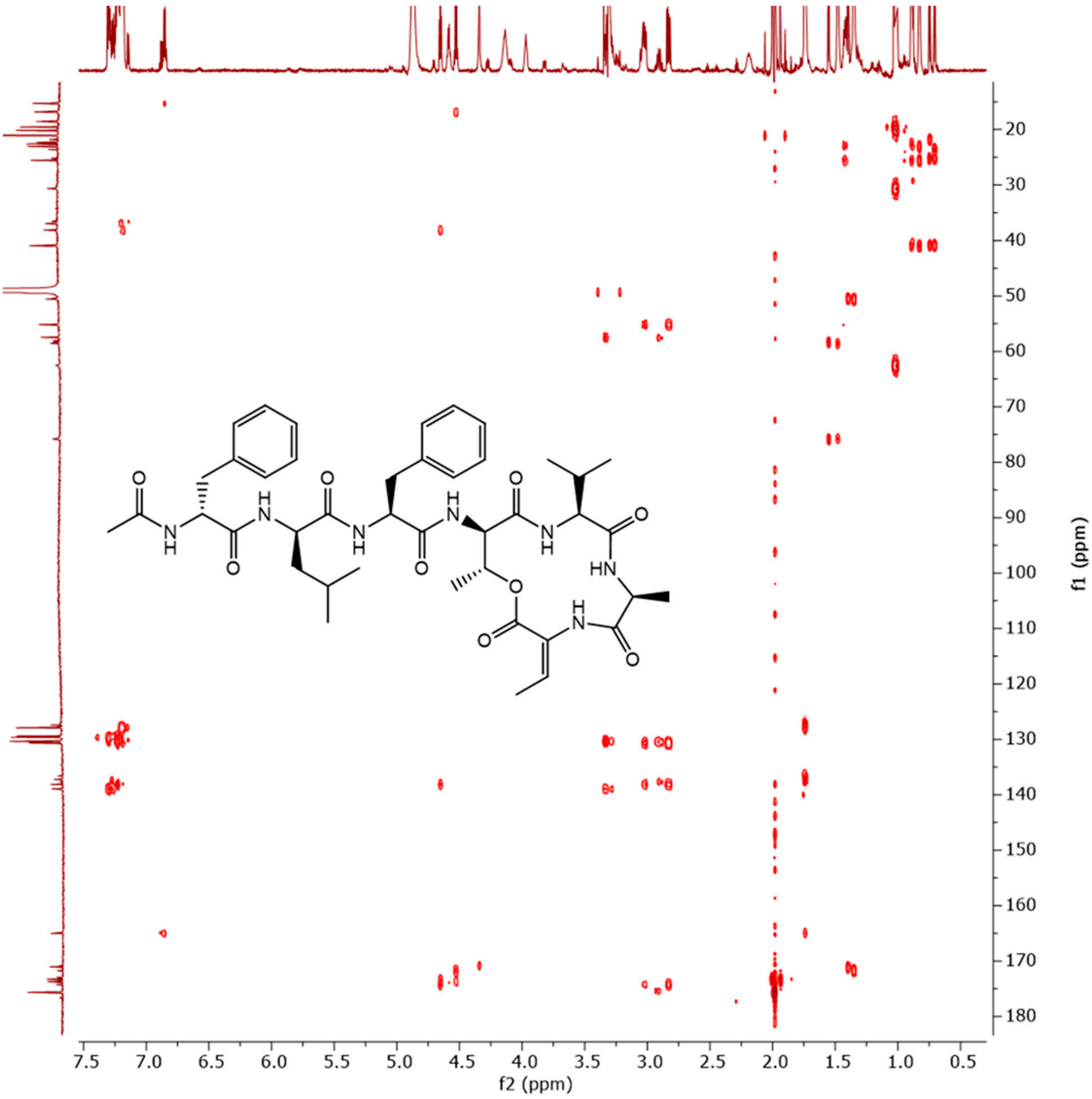

Figure S13. ^{1}H-^{13}C HMBC NMR spectrum (800 MHz, CD_3OD) of A-3302-B (**1**).

Key ^{1}H-^{1}H COSY and ^{1}H-^{13}C HMBC correlations of A-3302-B (1)

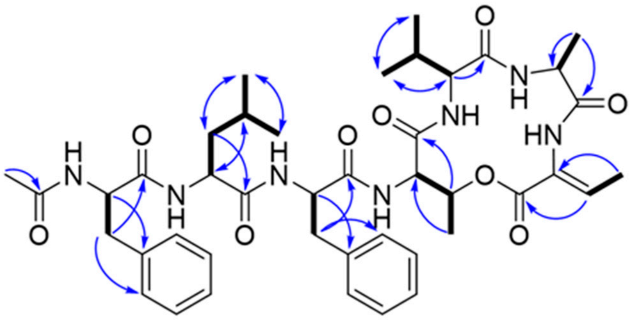

Figure S14. Key ^{1}H-^{1}H COSY and ^{1}H-^{13}C HMBC correlations of A-3302-B (**1**).

NMR data of A-3302-B (1)

7.1 mg, white amorphous powder.

^{1}H-NMR (800 MHz, CD_3OD): δ (ppm): 0.83 (d, *J* = 5.7 Hz, 3H), 0.89 (d, *J* = 5.8 Hz, 3H), 1.01 (d, *J* = 6.8 Hz, 3H), 1.03 (d, *J* = 6.6 Hz, 3H), 1.35 (d, *J* = 7.0 Hz, 3H), 1.36 – 1.38 (m, 1H), 1.41 – 1.45 (m, 2H), 1.48 (d, *J* = 6.4 Hz, 3H), 1.74 (d, *J* = 7.1 Hz, 3H), 2.00 (s, 3H), 2.16 – 2.22 (m, 1H), 2.83 (dd, *J* = 14.1, 7.3 Hz, 1H), 3.02 (dd, *J* = 14.2, 6.8 Hz, 1H), 3.04 – 3.06 (m, 1H), 3.32 – 3.35 (m, 1H), 3.95 – 3.99 (m, 1H), 4.12 – 4.16 (m, 1H), 4.34 (d, *J* = 4.0 Hz, 1H), 4.53 (q, *J* = 6.9 Hz, 1H), 4.59 (q, *J* = 6.8 Hz, 1H), 4.65 (t, *J* = 6.7 Hz, 1H), 6.85 (q, *J* = 7.1 Hz, 1H), 7.16 – 7.32 (m, 10H).

^{13}C-NMR (201 MHz, CD_3OD): δ (ppm): 15.26, 16.81, 18.53, 19.54, 20.13, 22.31, 22.57, 23.04, 25.54, 30.62, 36.93, 38.13, 40.95, 50.57, 55.16, 55.24, 57.46, 58.56, 62.54, 75.83, 127.41, 127.91, 129.39, 129.53, 129.61, 130.35, 130.48, 130.66, 136.61, 137.20, 137.64, 138.13, 138.92, 165.02, 171.03, 171.72, 172.94, 173.26, 173.91, 174.27, 175.64.

A-3302-A (2)

HR-ESI-MS of A-3302-A (2)

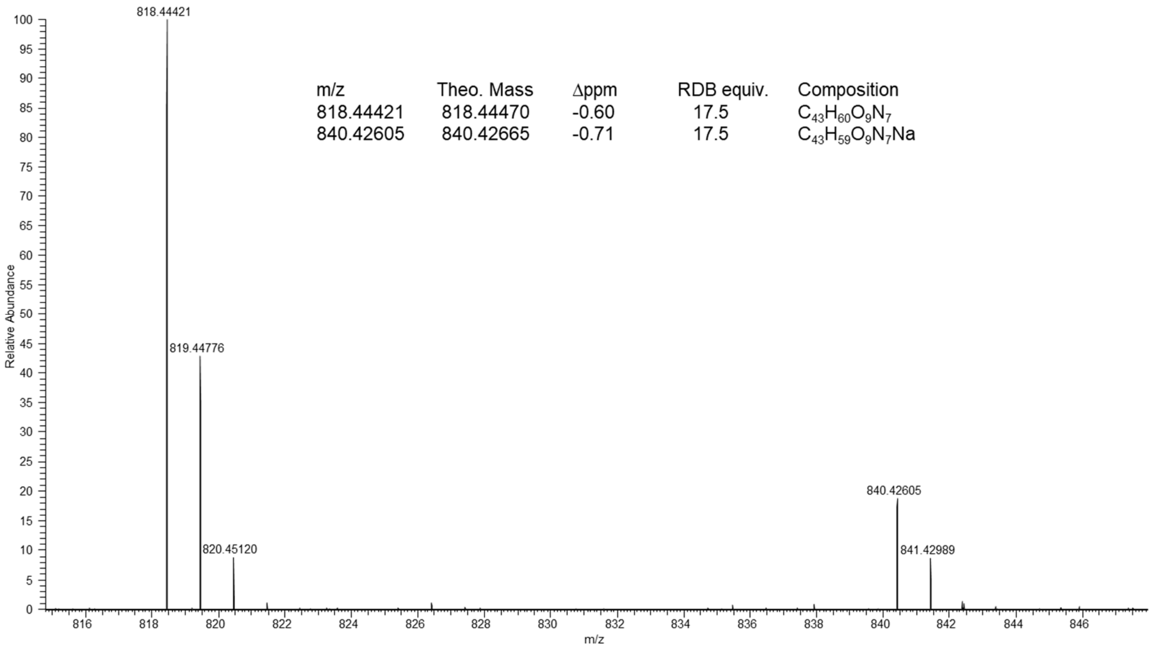

Figure S15. HR-ESI-MS of A-3302-A (**2**), retention time: 21.1 min (HPLC method: LC-HR-ESI-MS).

HR-MS/MS of A-3302-A (2)

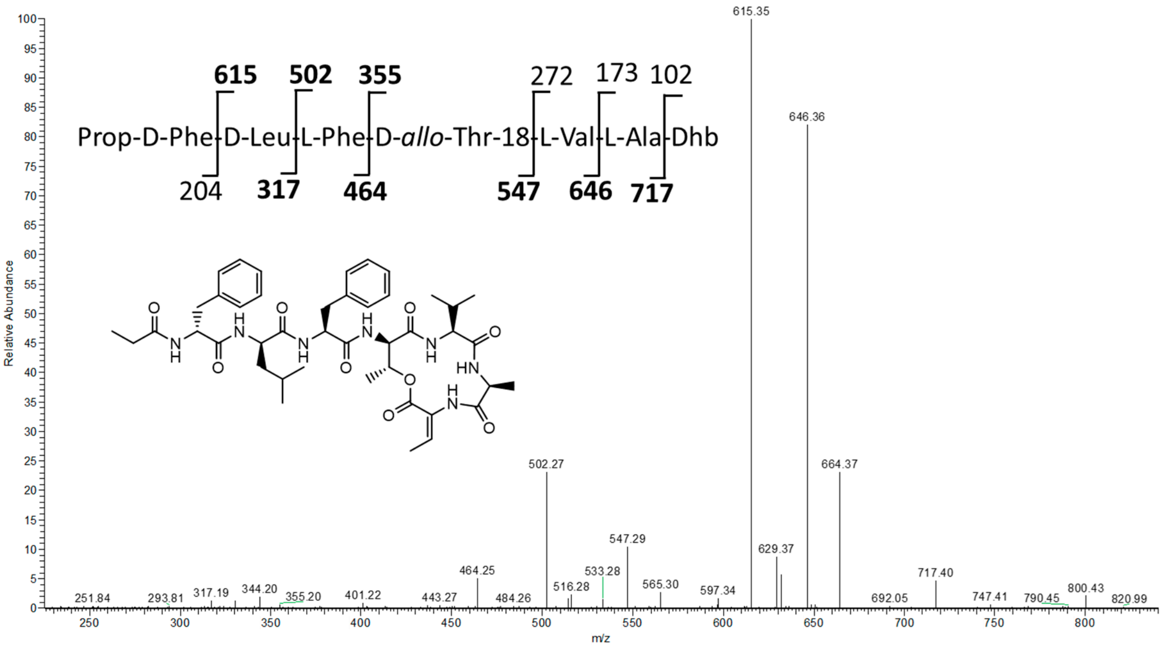

Figure S16. MS/MS of the quasimolecular ion $[M+H]^+$ *m/z* 818.4 of A-3302-A (**2**). The structure of **2** and the y and b peptide fragments after initial ring opening of the ester (*allo*-Thr-18) in the mass spectrometer are presented. The observed fragments are highlighted in bold. Dhb: *Z*-α,β-dehydrobutyrine.

Stereochemistry of the amino acids of A-3302-A (2)

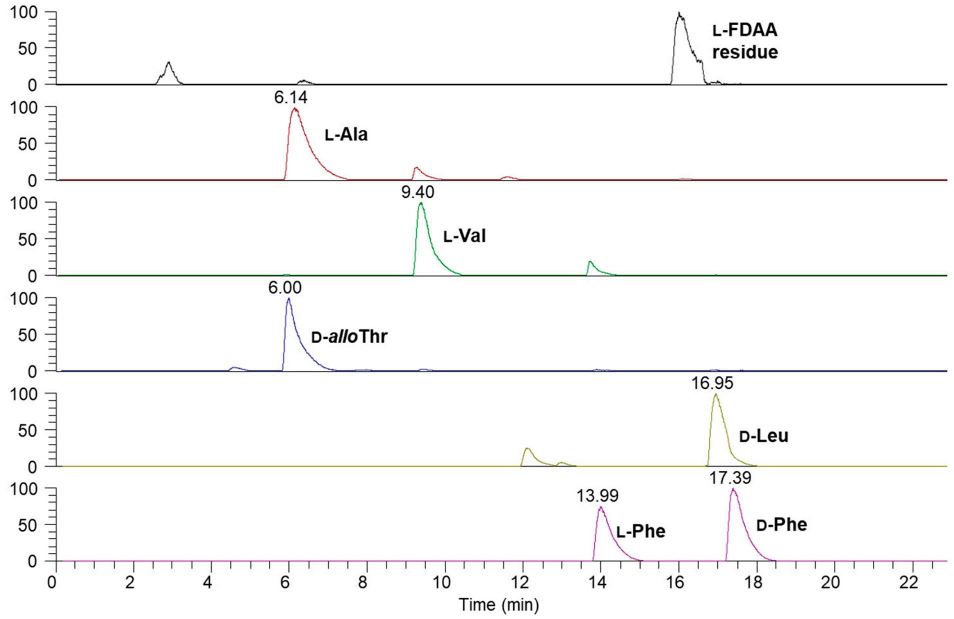

Figure S17: Stereochemistry of the amino acids of A-3302-A (**2**). The stereochemistry of the amino acids of A-3302-A (**2**) was analysed by LC-MS after acid hydrolysis of A-3302-A (**2**) and derivatization with Marfey's reagent **[37,38]**.

Nobilamide J (9)

HR-ESI-MS of nobilamide J (9)

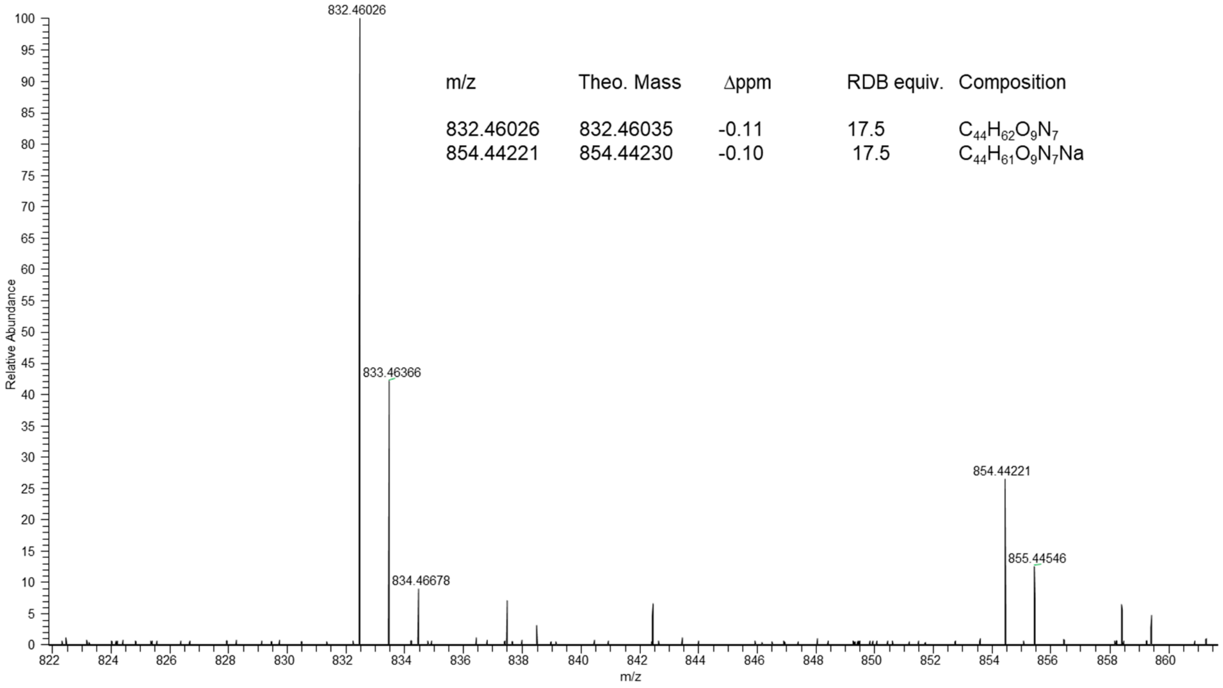

Figure S18. HR-ESI-MS of nobilamide J (**9**), retention time: 22.6 min (HPLC method: LC-HR-ESI-MS).

ESI-MS/MS of nobilamide J (9)

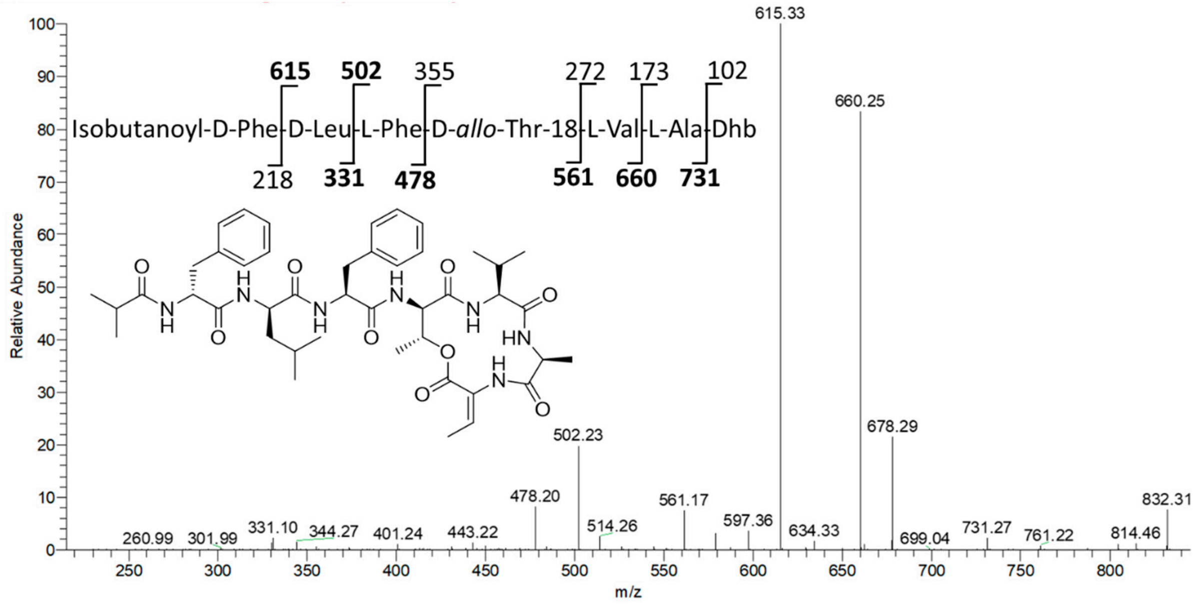

Figure S19: ESI-MS/MS of nobilamide J (**9**). Nobilamide J (**9**) was identified by MS/MS of its quasimolecular ion $[M+H]^+$ *m/z* 832.4. The structure of **9** and the y and b fragments after initial ring opening of the ester (alloThr-18) in the mass spectrometer are presented. The observed fragments are highlighted in bold. Dhb: *Z*-α,β-dehydrobutyrine.

Stereochemistry of the amino acids of nobilamide J (9)

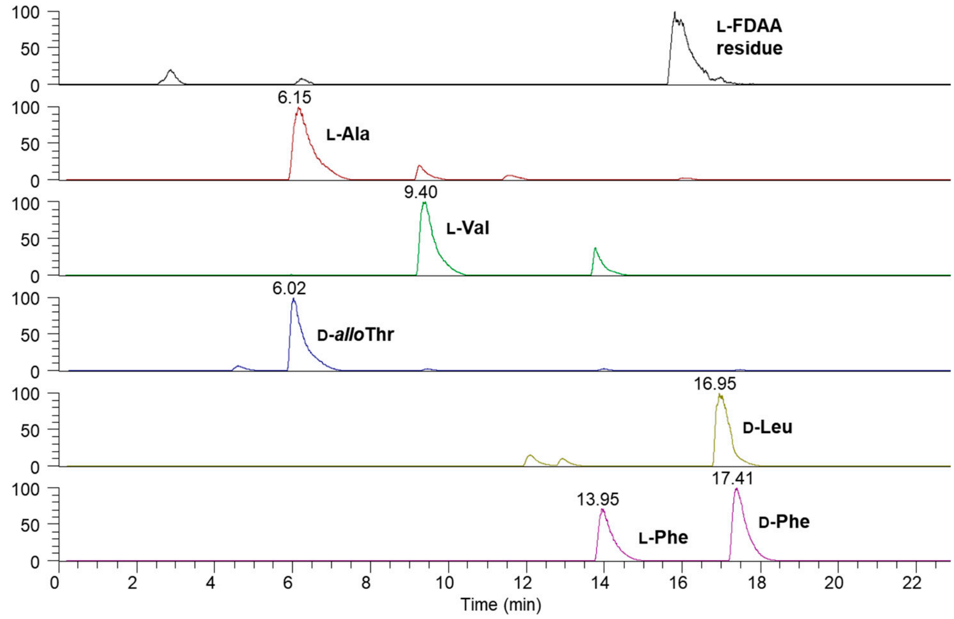

Figure S20: Stereochemistry of the amino acids of nobilamide J (**9**). The stereochemistry of the amino acids of nobilamide J (**9**) was analysed by LC-MS after acid hydrolysis of nobilamide J (**9**) and derivatization with Marfey's reagent **[37,38]**.

^{1}H NMR spectrum of nobilamide J (9)

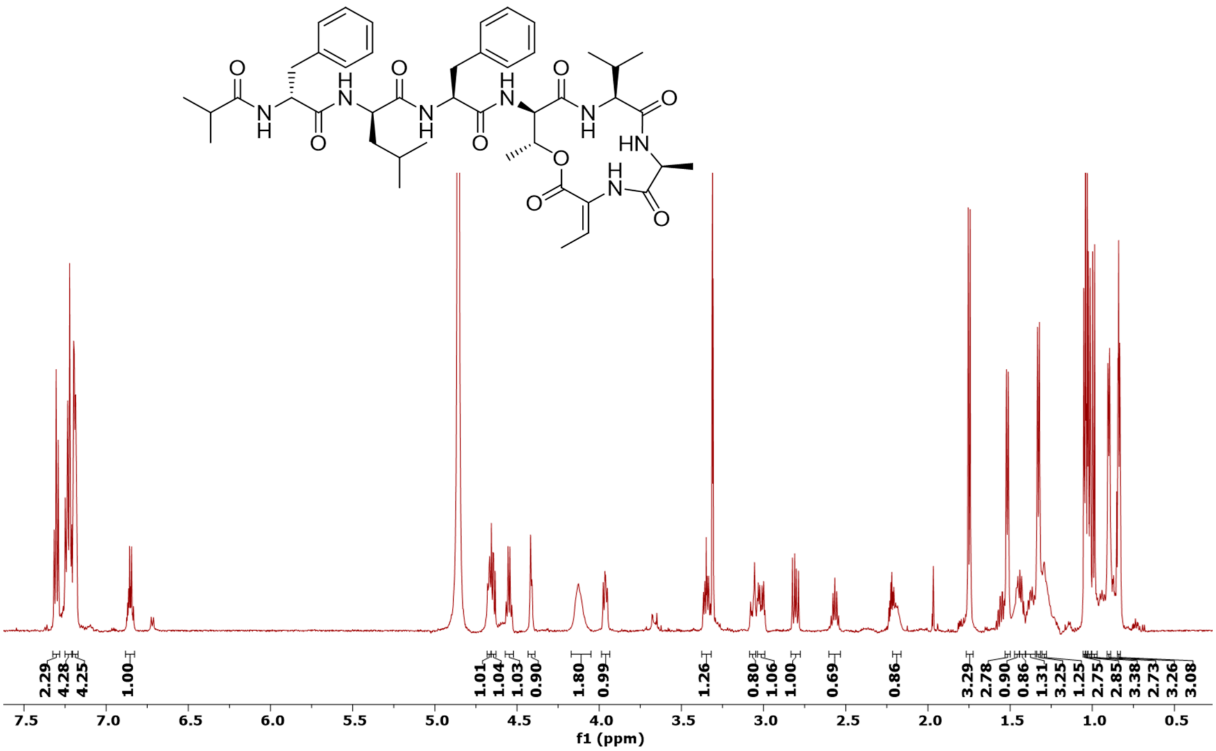

Figure S21. ^{1}H NMR spectrum (600 MHz, CD_3OD) of nobilamide J (**9**).

^{13}C NMR spectrum of nobilamide J (9)

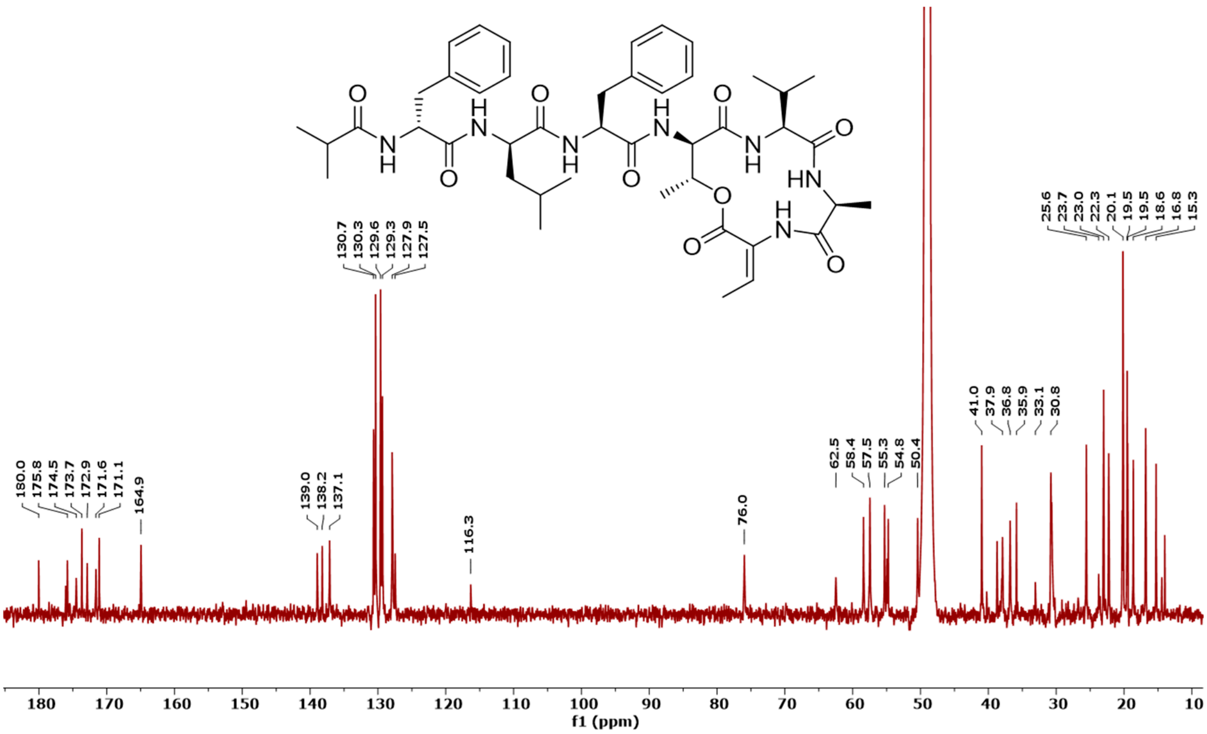

Figure S22. ^{13}C NMR spectrum (151 MHz, CD_3OD) of nobilamide J (**9**).

^{1}H-^{1}H COSY NMR spectrum of nobilamide J (9)

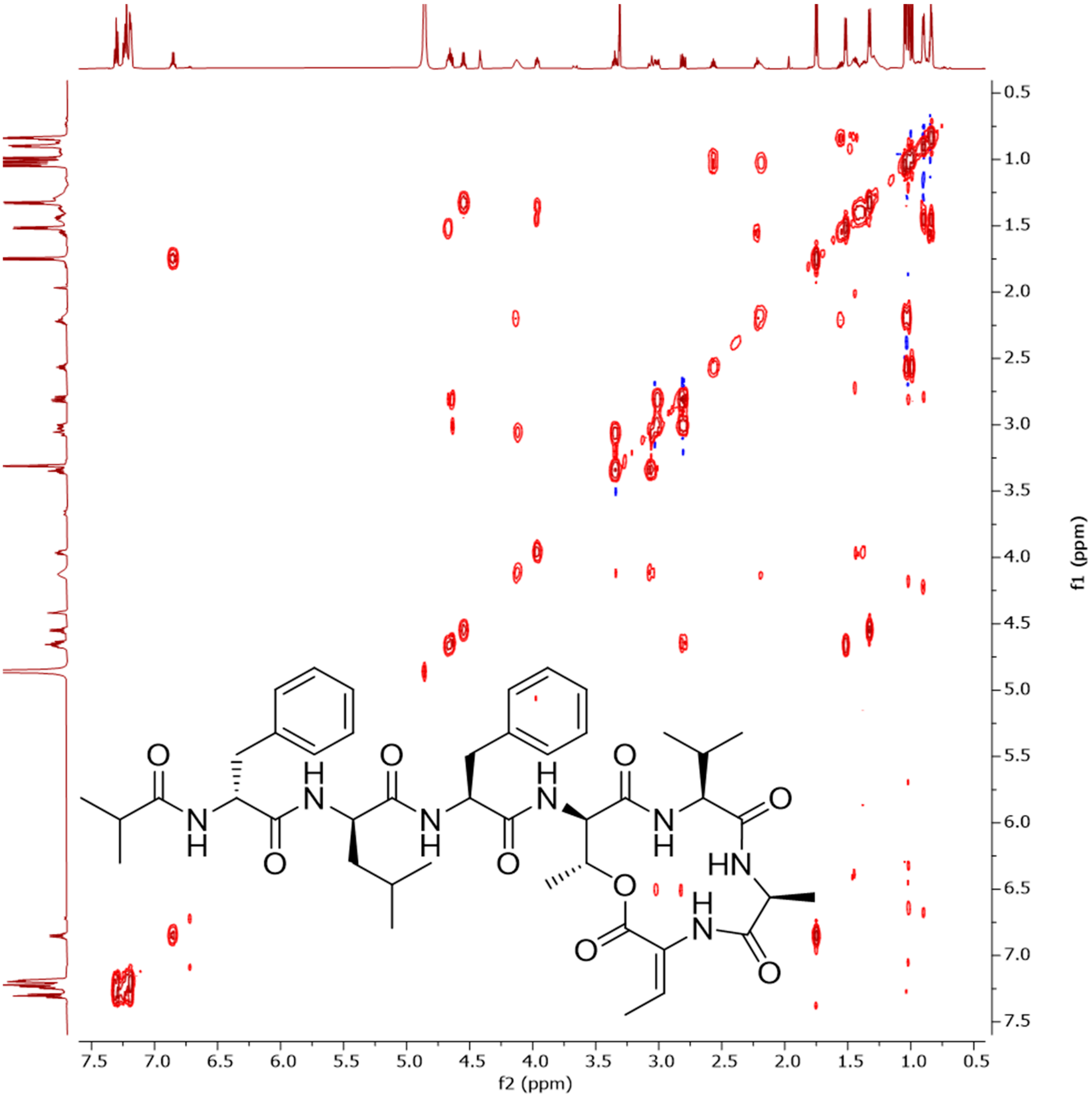

Figure S23. ^{1}H-^{1}H COSY NMR spectrum (600 MHz, CD_3OD) of nobilamide J (**9**).

^{1}H-^{13}C HSQC NMR spectrum of nobilamide J (9)

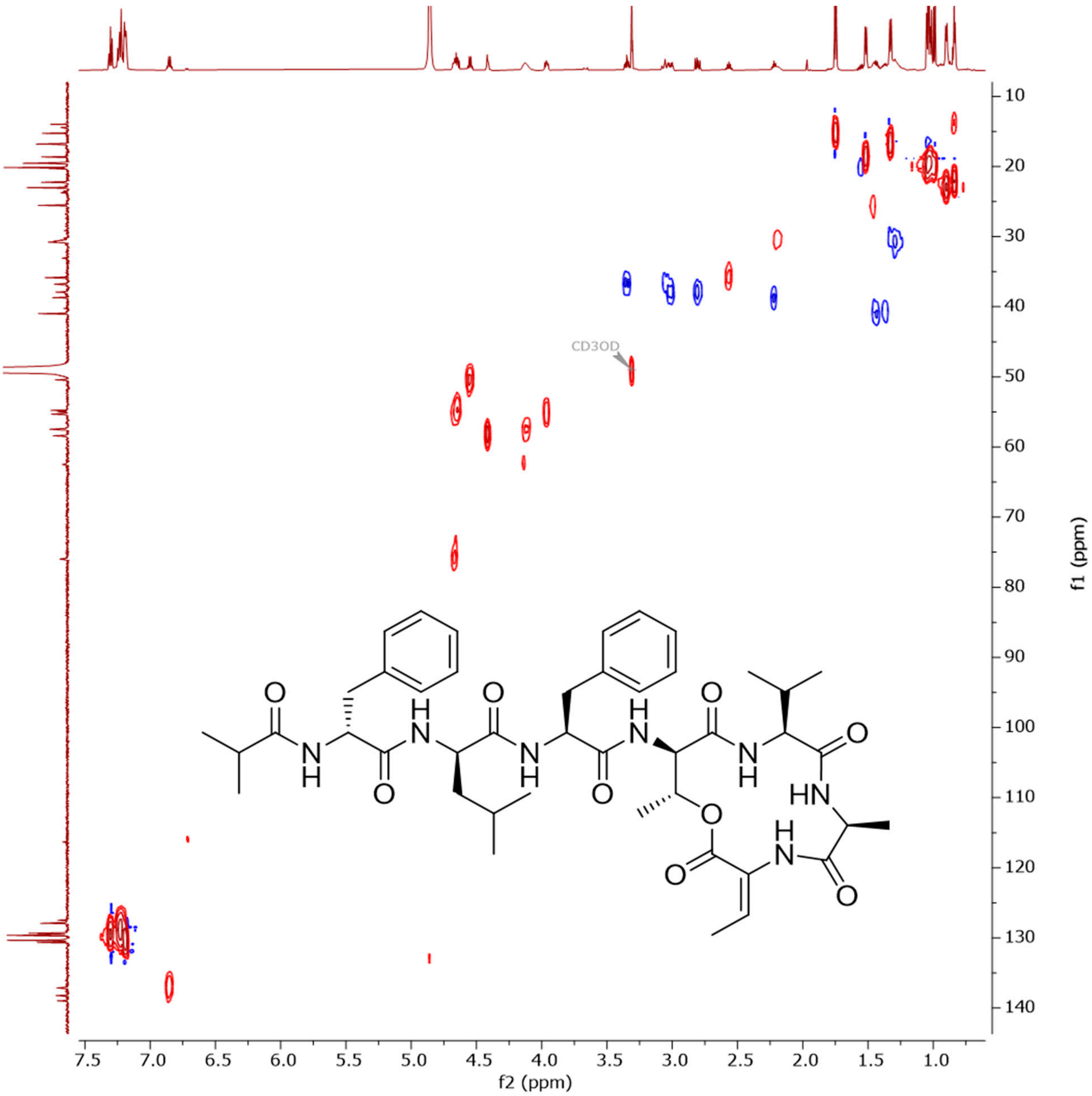

Figure S24. ^{1}H-^{13}C HSQC NMR spectrum (600 MHz, CD_3OD) of nobilamide J (**9**).

HMBC NMR spectrum of nobilamide J (9)

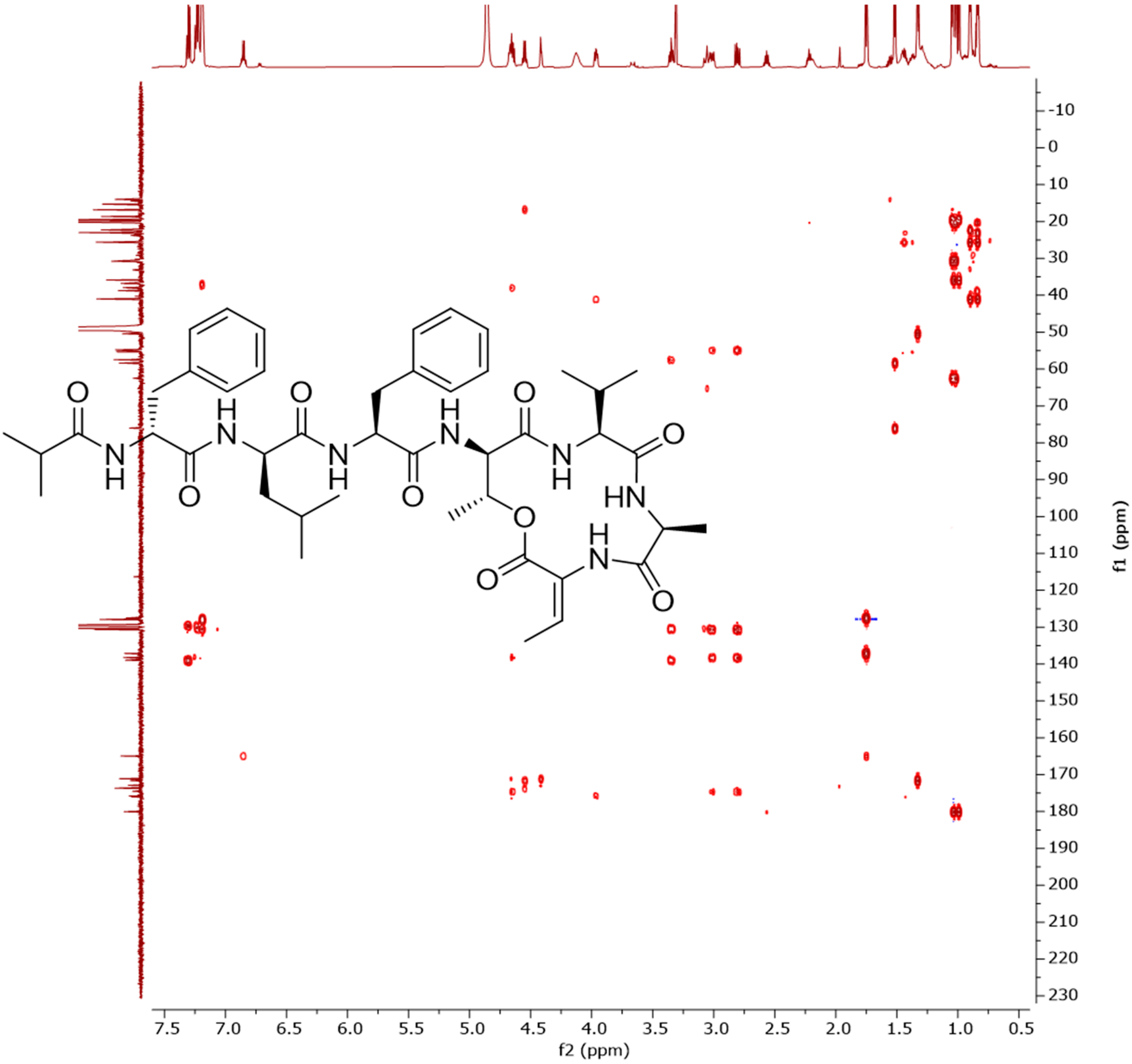

Figure S25. ^{1}H-^{13}C-HMBC NMR spectrum (600 MHz, CD_3OD) of nobilamide J (**9**).

Key ^{1}H-^{1}H COSY and ^{1}H-^{13}C HMBC correlations of nobilamide J (9)

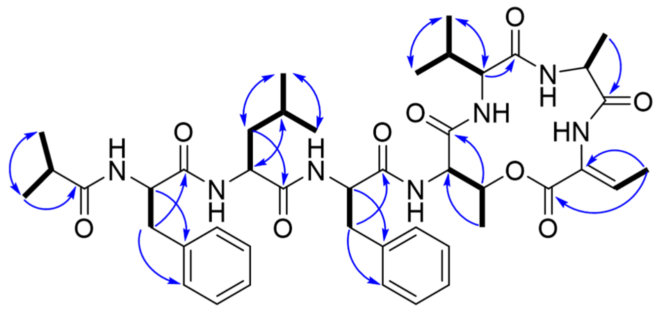

Figure S26. Key ^{1}H-^{1}H COSY and ^{1}H-^{13}C HMBC correlations of nobilamide J (**9**)

NMR data of nobilamide J (9)

0.8 mg, white amorphous powder.

^{1}H-NMR (600 MHz, CD_3OD): δ (ppm): 0.84 (d, *J* = 6.3 Hz, 3H), 0.90 (d, *J* = 6.4 Hz, 3H), 0.99 (d, *J* = 7.0 Hz, 3H), 1.02 (d, *J* = 6.8 Hz, 3H), 1.04 (d, *J* = 7.0 Hz, 3H), 1.05 (d, *J* = 6.8 Hz, 3H), 1.33 (d, *J* = 6.9 Hz, 3H), 1.35 – 1.41 (m, 1H), 1.40 – 1.48 (m, 1H), 1.44 – 1.47 (m, 1H), 1.52 (d, *J* = 6.6 Hz, 3H), 1.75 (d, *J* = 7.3 Hz, 3H), 2.15 – 2.25 (m, 1H), 2.57 (p, *J* = 6.9 Hz, 1H), 2.81 (dd, *J* = 14.1, 8.1 Hz, 1H), 2.98 – 3.09 (m, 1H), 3.05 – 3.08 (m, 1H), 3.35 (dd, *J* = 13.8, 5.5 Hz, 1H), 3.96 (dd, *J* = 8.9, 5.9 Hz, 1H), 4.07 – 4.17 (m, 2H), 4.41 (d, *J* = 5.1 Hz, 1H), 4.55 (d, *J* = 6.9 Hz, 1H), 4.64 (dd, *J* = 8.2, 5.8 Hz, 1H), 4.65 – 4.68 (m, 1H), 6.85 (qd, *J* = 7.2, 3.6 Hz, 1H), 7.16 – 7.34 (m, 10H).

^{13}C-NMR (201 MHz, CD_3OD): δ (ppm): 15.27, 16.81, 18.64, 19.46, 19.55, 20.15, 22.26, 23.02, 23.74, 25.56, 30.78, 33.07, 35.85, 36.79, 37.92, 40.98, 50.43, 54.77, 55.31, 57.47, 58.41, 62.49, 75.98, 127.48, 127.91, 129.34, 129.62, 130.35, 130.66, 137.13, 138.23, 138.96, 164.95, 171.11, 171.59, 172.87, 173.67, 174.49, 175.79, 180.02.

Nobilamide K (10)

HR-ESI-MS of nobilamide K (10)

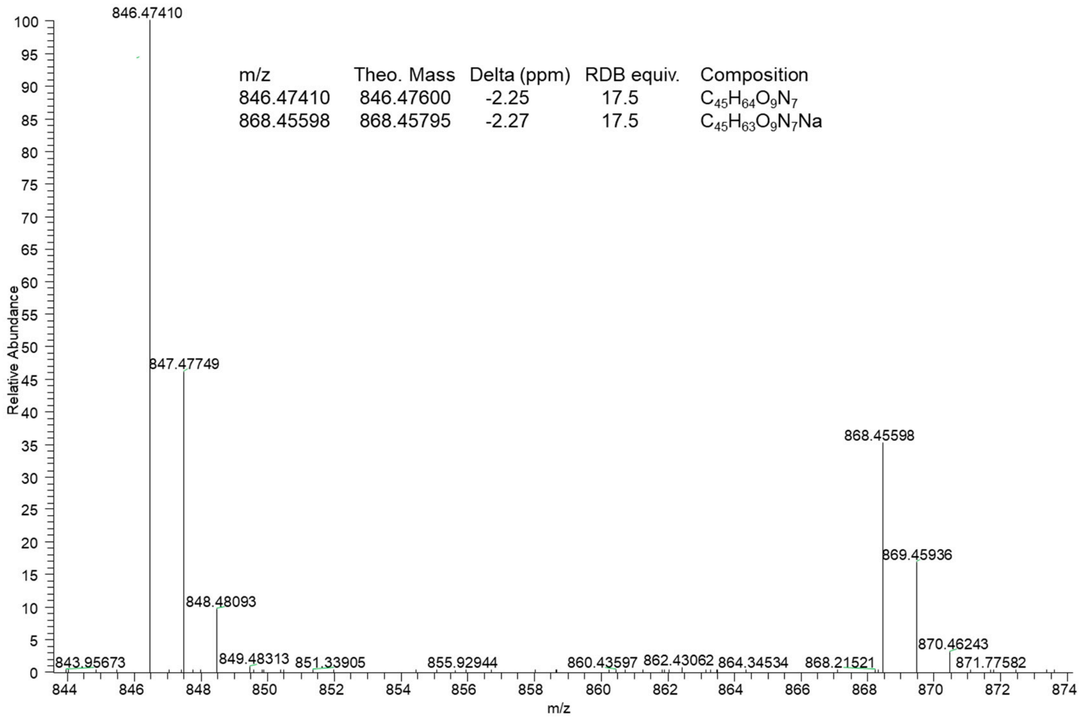

Figure S27. HR-ESI-MS of nobilamide K (**10**), retention time 23.8 min (HPLC method: LC-HR-ESI-MS).

ESI-MS/MS of nobilamide K (10)

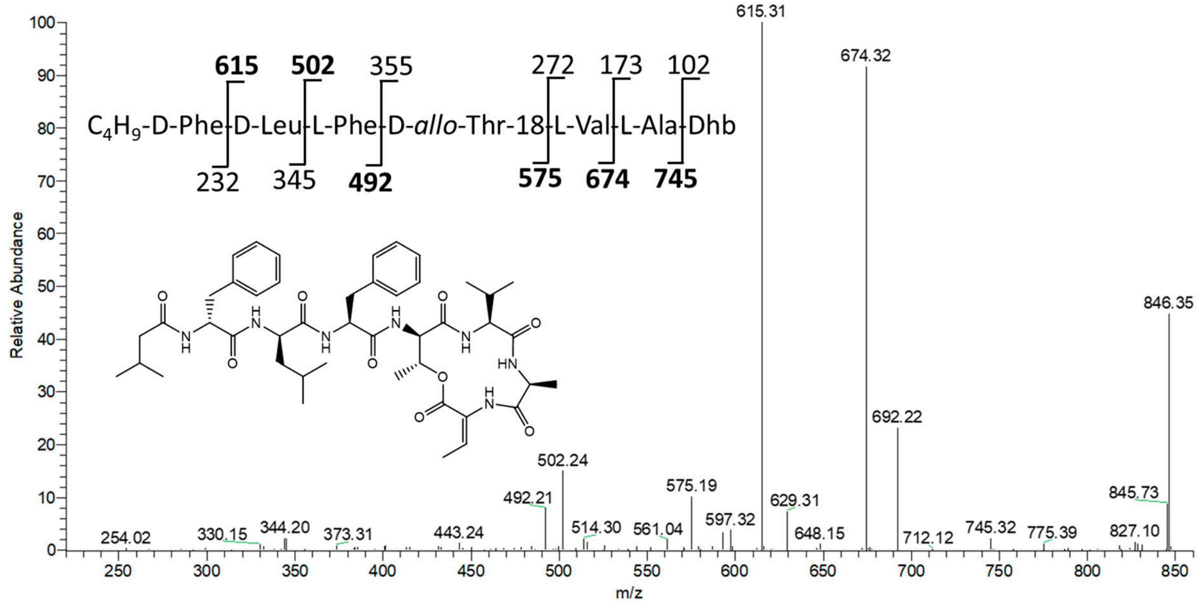

Figure S28. ESI-MS/MS of nobilamide K (**10**). MS/MS of the quasimolecular ion *m/z* 846.4 of nobilamide K (**10**). The structure of **10** and the y and b fragments after initial ring opening of the ester (alloThr-18) in the mass spectrometer are presented. The observed fragments are highlighted in bold. Dhb: *Z*-α,β-dehydrobutyrine. Nobilamide K (**10**) is a minor component.

Stereochemistry of the amino acids of nobilamide K (10)

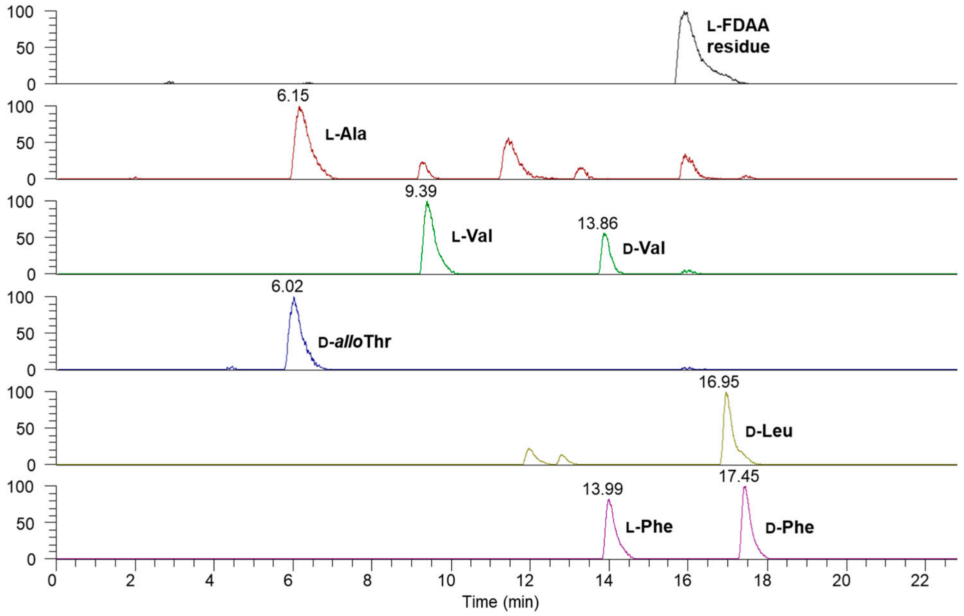

Figure S29. Stereochemistry of the amino acids of nobilamide K (**10**). The stereochemistry of the amino acids of nobilamide K (**10**) was analysed by LC-MS after acid hydrolysis of nobilamide K (**10**) and derivatization with Marfey's reagent **[37,38]**.

Nobilamide L (11)

HR-ESI-MS of nobilamide L (11)

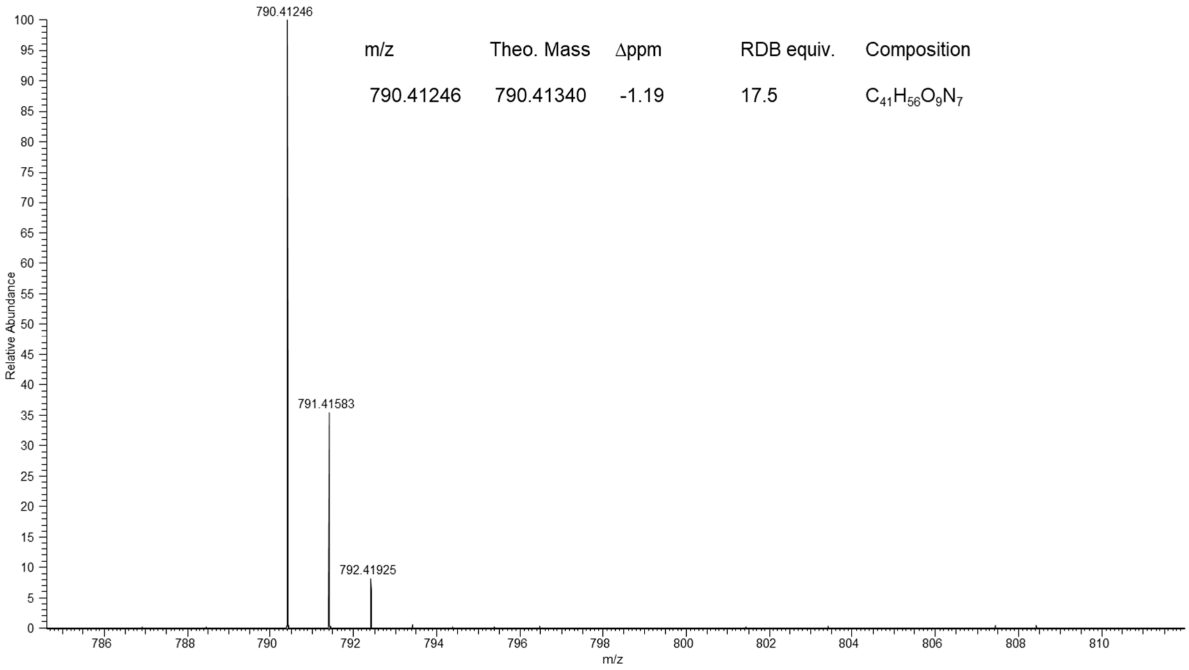

Figure S30. HR-ESI-MS of nobilamide L (**11**), retention time 17.7 min (HPLC method: LC-HR-ESI-MS).

ESI-MS/MS of nobilamide L (11)

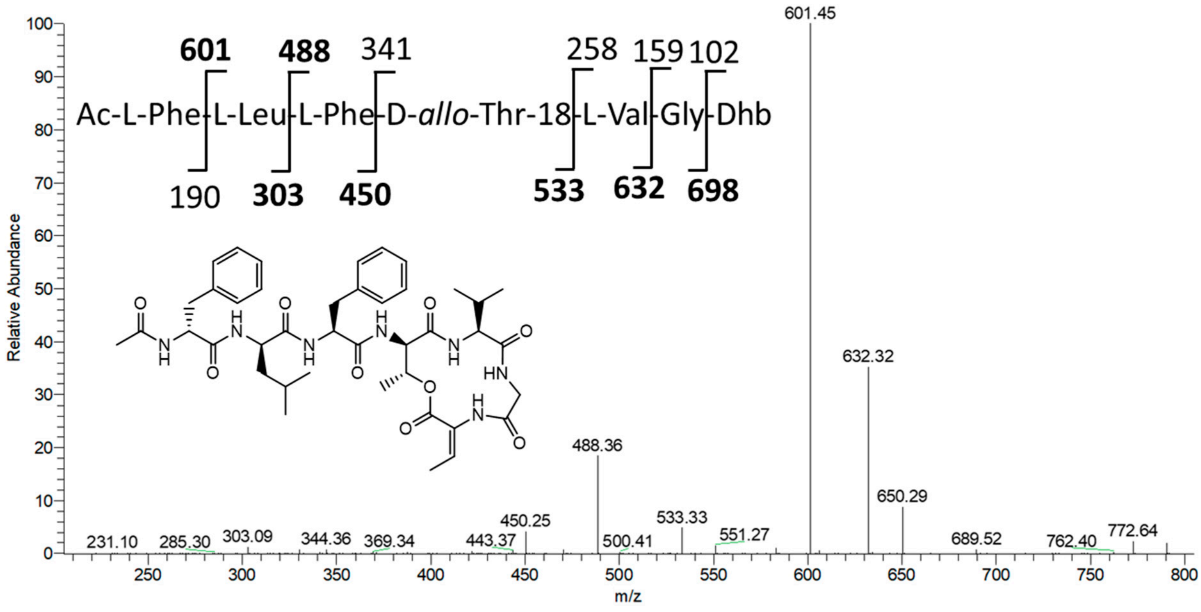

Figure S31. ESI-MS/MS of nobilamide L (**11**). MS/MS of the quasimolecular ion $[M+H]^+$ *m/z* 790.4 of nobilamide L (**11**). The structure of **5** and the y and b fragments after initial ring opening of the ester (alloThr-18) in the mass spectrometer are presented. The observed fragments are highlighted in bold. Dhb: *Z*-α,β-dehydrobutyrine.

Stereochemistry of the amino acids of nobilamide L (11)

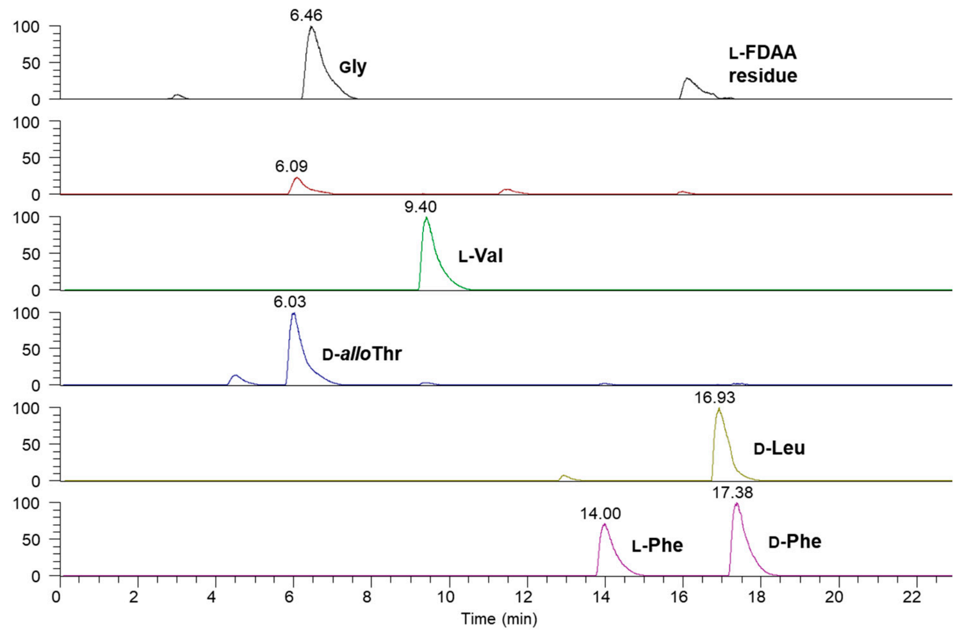

Figure S32. Stereochemistry of the amino acids of nobilamide L (**11**). The stereochemistry of the amino acids of nobilamide L (**11**) was analysed by LC-MS after acid hydrolysis of nobilamide L (**11**) and derivatization with Marfey's reagent **[37,38]**.

¹H NMR spectrum of nobilamide L (11)

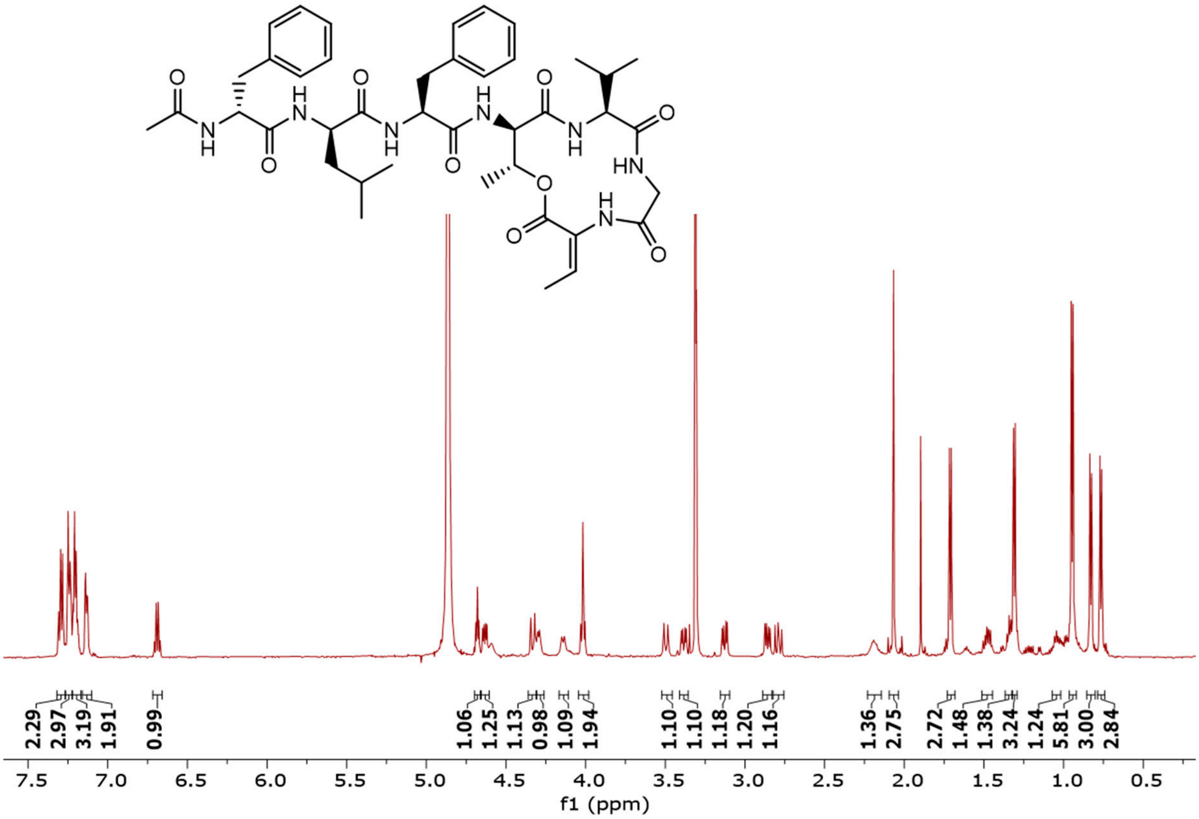

Figure S33. ¹H NMR spectrum (600 MHz, CD_3OD) of nobilamide L (**11**).

¹³C NMR spectrum of nobilamide L (11)

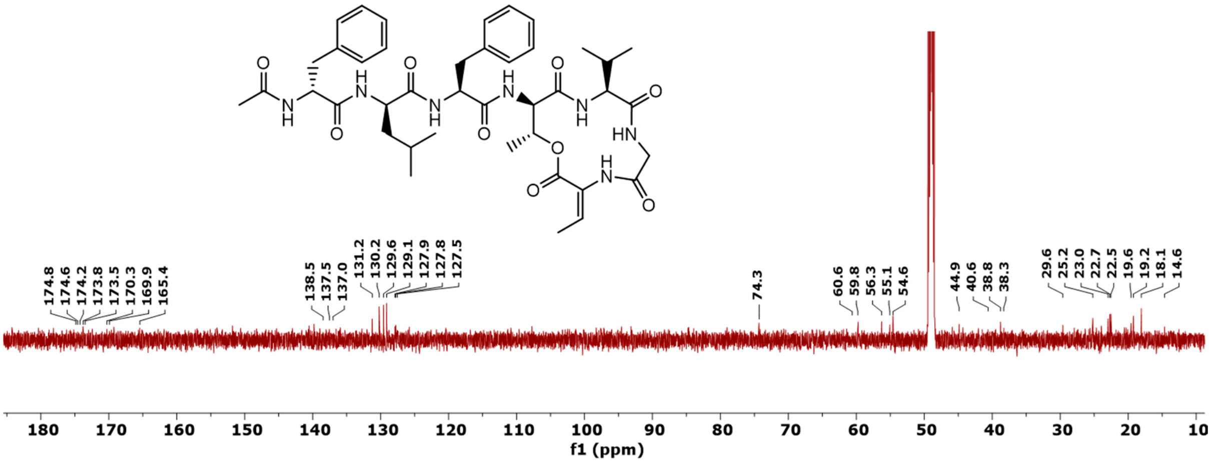

Figure S34. ¹³C NMR spectrum (151 MHz, CD_3OD) of nobilamide L (**11**).

^{1}H-^{1}H COSY NMR spectrum of nobilamide L (11)

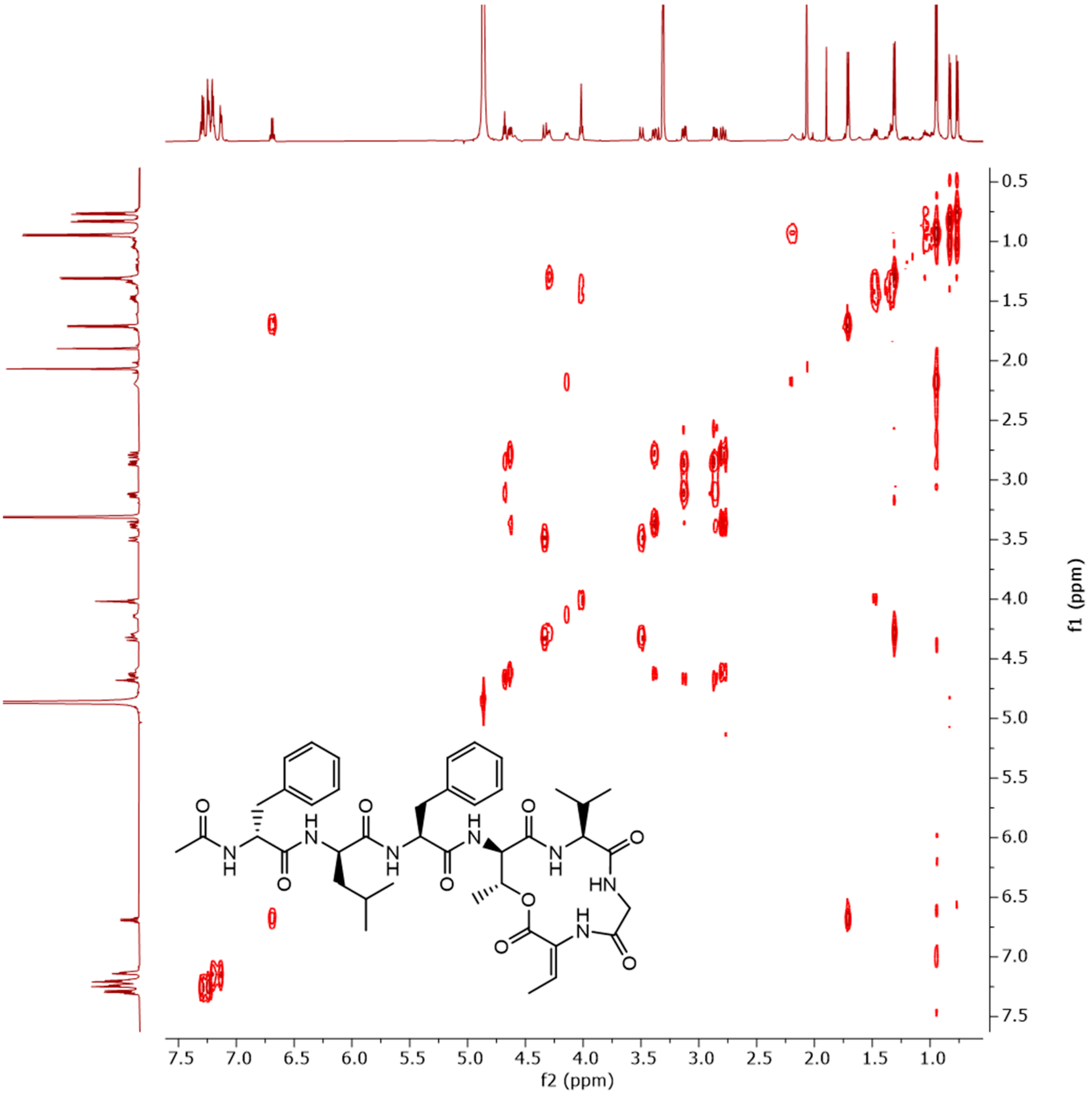

Figure S35. ^{1}H-^{1}H COSY NMR spectrum (600 MHz, CD_3OD) of nobilamide L (**11**).

^{1}H-^{13}C HSQC NMR spectrum (600 MHz, CD3OD) of nobilamide L (11)

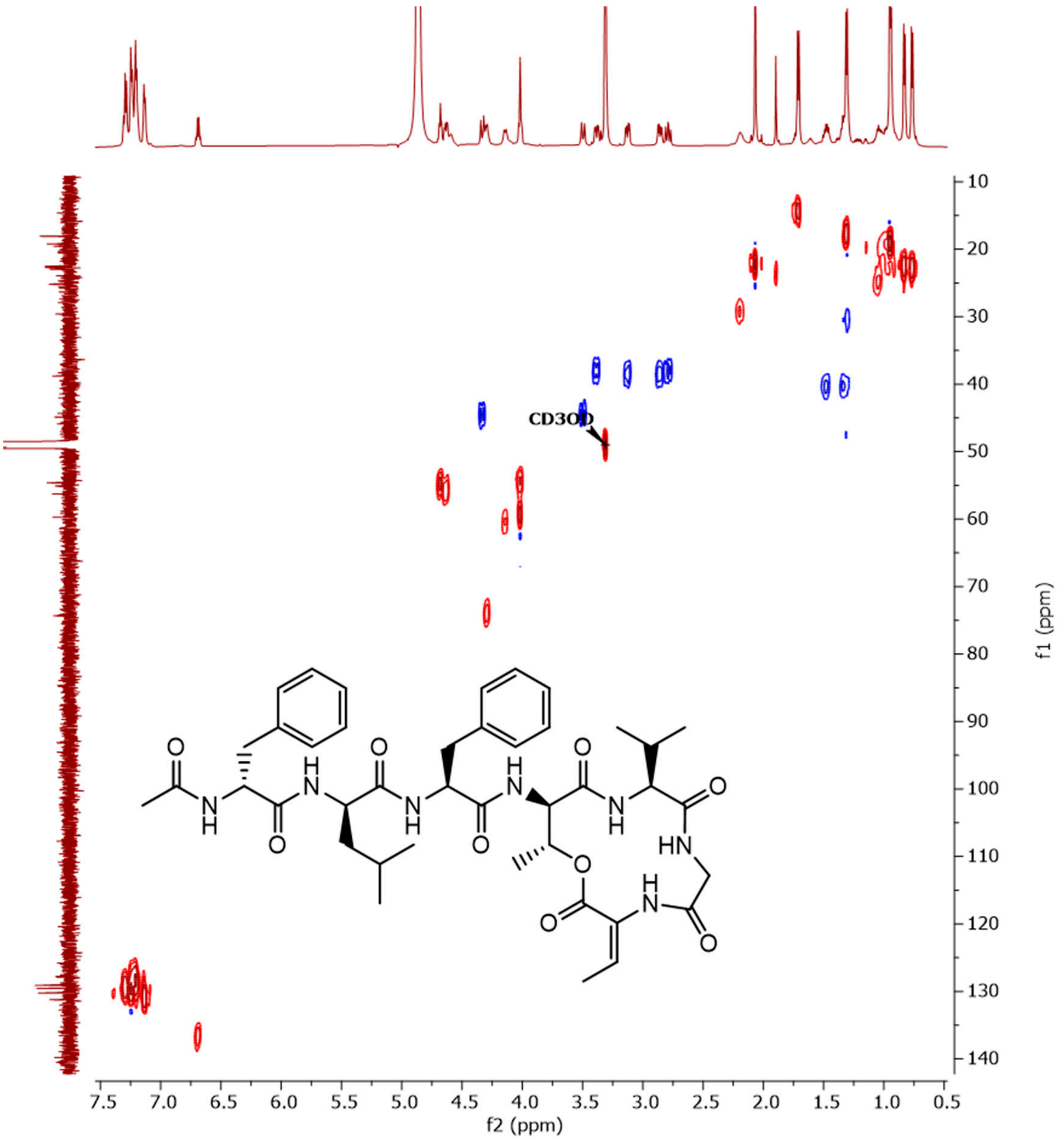

Figure S36. ^{1}H-^{13}C HSQC NMR spectrum (600 MHz, CD_3OD) of nobilamide L (**11**).

¹H-¹³C HMBC NMR spectrum of nobilamide L (11)

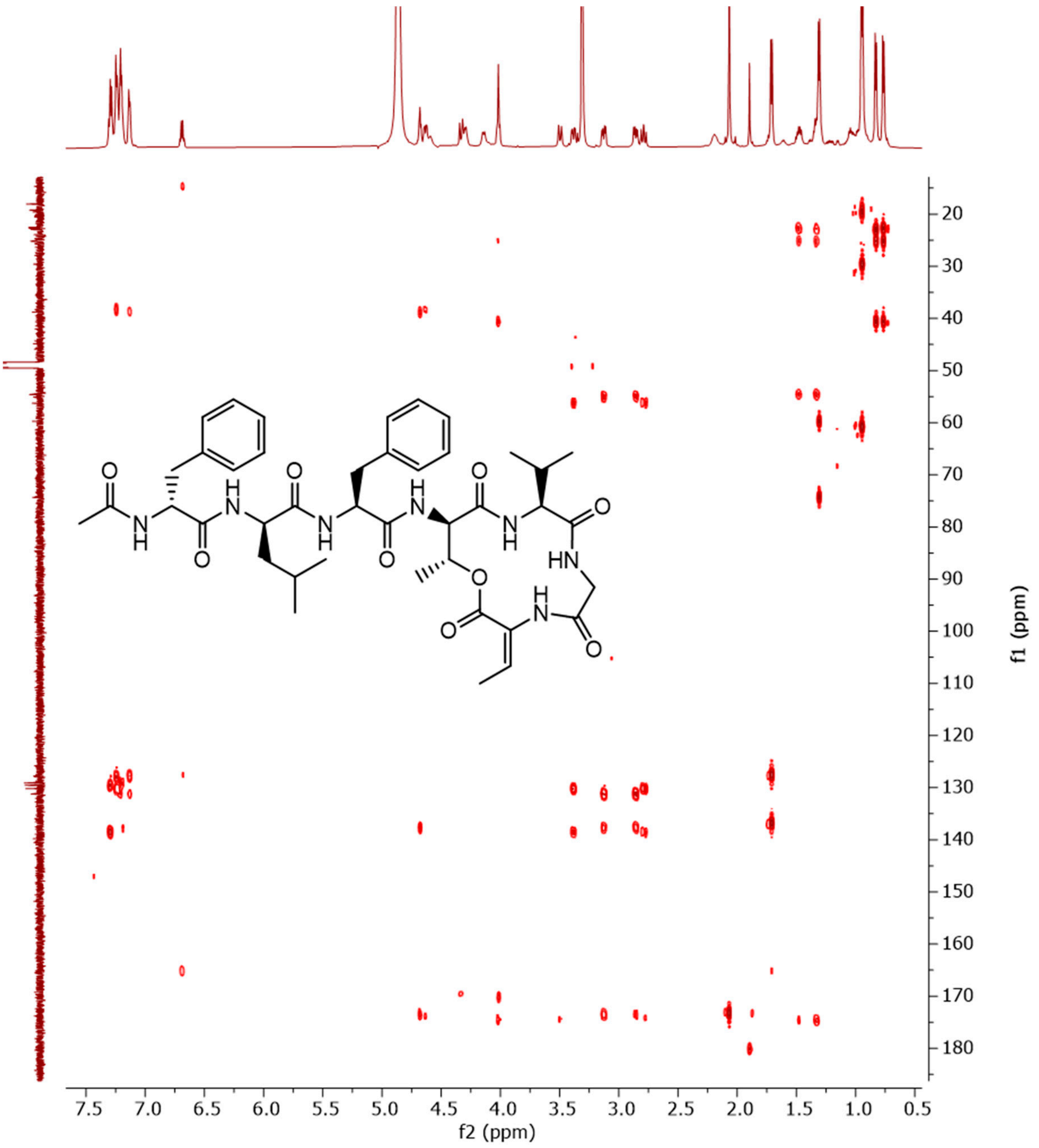

Figure S37. ¹H-¹³C HMBC NMR spectrum (600 MHz, CD_3OD) of nobilamide L (**11**).

Key ^{1}H-^{1}H COSY and ^{1}H-^{13}C HMBC NMR spectrum of nobilamide L (11)

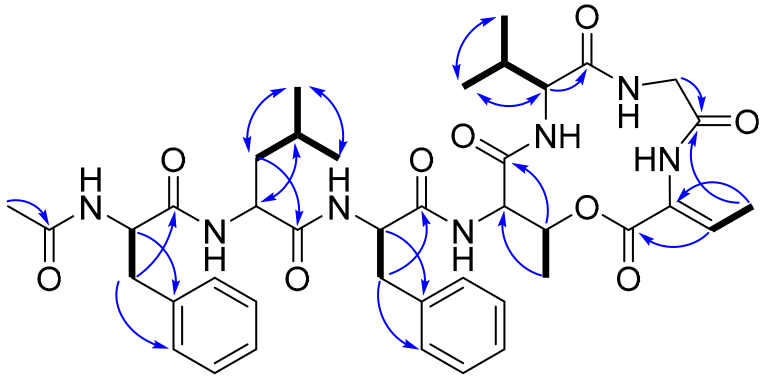

Figure S38. Key ^{1}H-^{1}H COSY and ^{1}H-^{13}C HMBC NMR spectrum (600 MHz, CD_3OD) of nobilamide L (**11**).

NMR data of nobilamide L (11)

1.0 mg, white amorphous powder.

^{1}H-NMR (600 MHz, CD_3OD): δ (ppm): 0.77 (d, *J* = 6.5 Hz, 3H), 0.83 (d, *J* = 6.5 Hz, 3H), 0.95 (d, *J* = 6.6 Hz, 6H), 1.05 (dt, *J* = 13.7, 6.7 Hz, 1H), 1.31 (d, *J* = 6.5 Hz, 3H), 1.48 (ddd, *J* = 14.0, 8.1, 6.2 Hz, 1H), 1.71 (d, *J* = 7.2 Hz, 3H), 2.07 (s, 3H), 2.17 (s, 1H), 2.79 (dd, *J* = 13.8, 11.2 Hz, 1H), 2.86 (dd, *J* = 13.9, 5.8 Hz, 1H), 3.13 (dd, *J* = 13.9, 5.4 Hz, 1H), 3.38 (dd, *J* = 13.8, 5.0 Hz, 1H), 3.50 (d, *J* = 14.7 Hz, 1H), 3.98 – 4.04 (m, 2H), 4.14 (d, *J* = 10.2 Hz, 1H), 4.30 (d, *J* = 6.6 Hz, 1H), 4.33 (d, *J* = 14.7 Hz, 1H), 4.63 (dd, *J* = 11.2, 4.9 Hz, 1H), 4.68 (t, *J* = 5.6 Hz, 1H), 6.69 (q, *J* = 7.2 Hz, 1H), 7.10 – 7.16 (m, 2H), 7.17 – 7.22 (m, 3H), 7.24 (dt, *J* = 8.3, 2.0 Hz, 3H), 7.27 – 7.32 (m, 2H).

^{13}C-NMR (151 MHz, CD_3OD): δ (ppm): 14.64, 18.06, 19.23, 19.57, 22.54, 22.71, 22.97, 25.20, 29.60, 38.30, 38.79, 40.60, 44.87, 54.61, 55.07, 56.28, 59.78, 60.61, 74.30, 127.55, 127.77, 127.90, 129.11, 129.58, 130.21, 131.22, 137.01, 137.53, 138.46, 165.43, 169.90, 170.29, 173.49, 173.78, 174.24, 174.59, 174.84.

Nobilamide M (12)

HR-ESI-MS of nobilamide M (12)

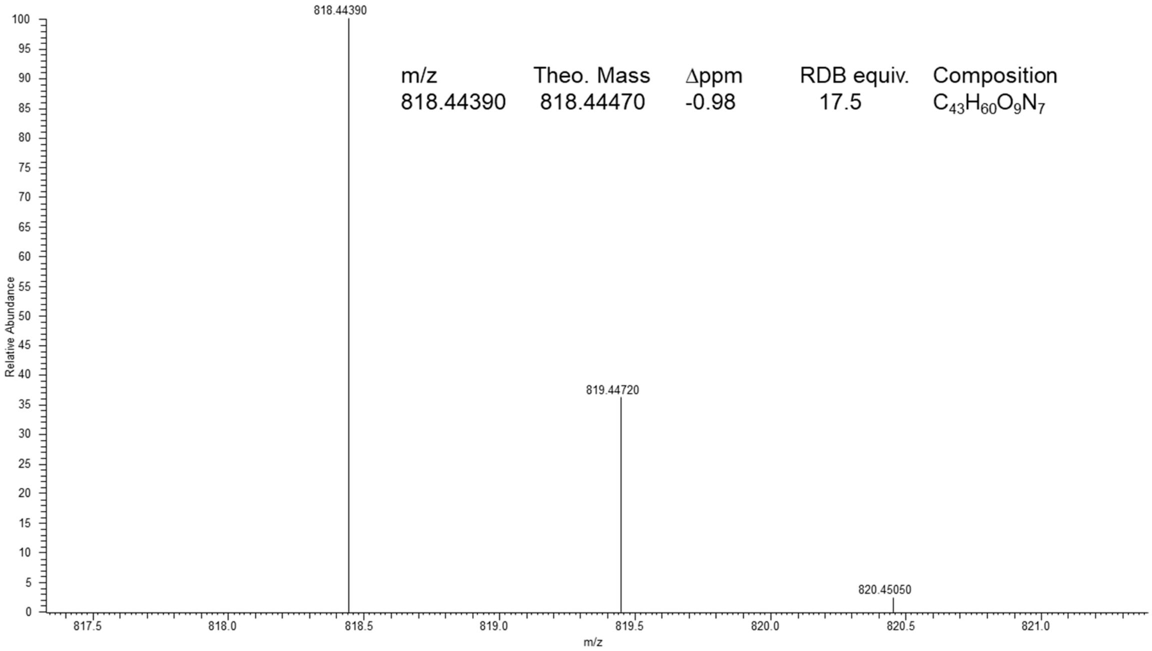

Figure S39. HR-ESI-MS of nobilamide M (**12**), retention time: 20.5 min (HPLC method: LC-HR-ESI-MS).

ESI-MS/MS of nobilamide M (12)

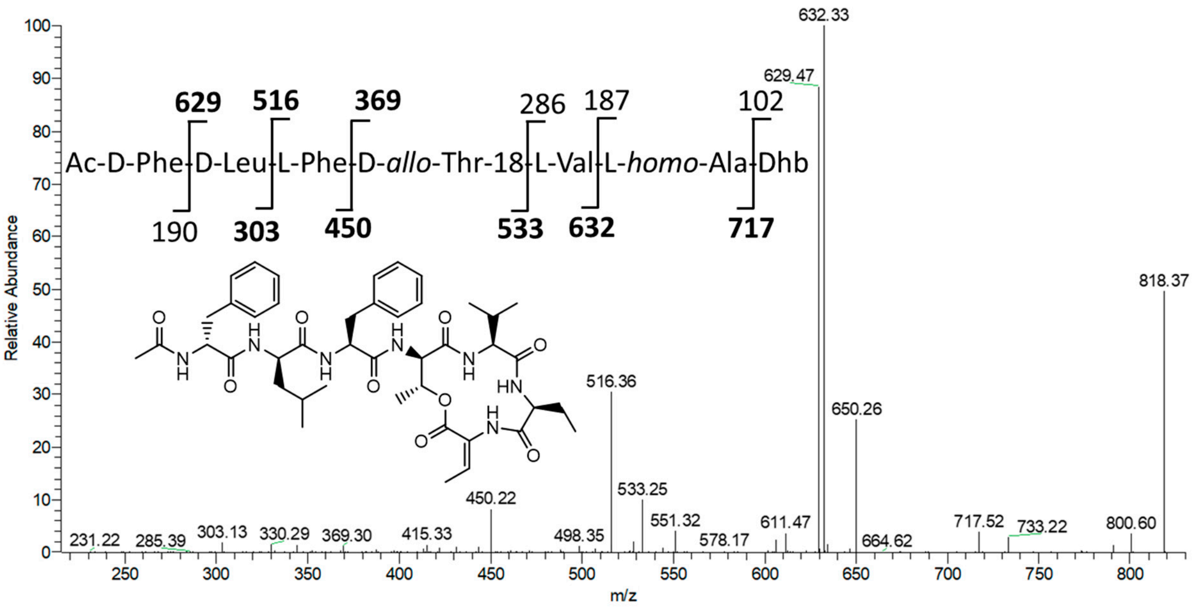

Figure S40. ESI-MS/MS of nobilamide M (**12**). MS/MS of the quasimolecular ion $[M+H]^+$ *m/z* 818.4 of nobilamide M (**12**). The structure of **12** and the y and b fragments after initial ring opening (D-*allo*-Thr-18) of the ester in the mass spectrometer are presented. The observed fragments are highlighted in bold. Dhb: *Z*-α,β-dehydrobutyrine.

Stereochemistry of the amino acids of nobilamide M (12).

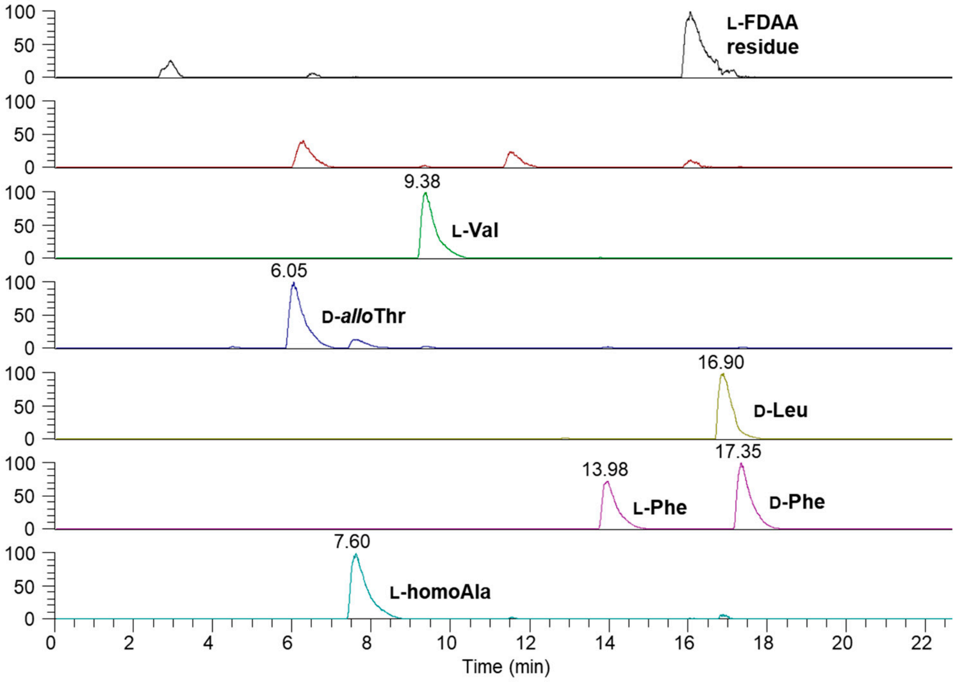

Figure S41. Stereochemistry of the amino acids of nobilamide M (**12**). The stereochemistry of the amino acids of nobilamide M (**12** was analysed by LC-MS after acid hydrolysis of nobilamide M (**12**) and derivatization with Marfey's reagent **[37,38**].

The stereochemistry of the homoalanine was not determined due to lack of a standard but tentatively assigned as L-isomer in analogy to L-alanine that is normally found in this position.

Nobilamide N (13)

HR-ESI-MS of nobilamide N (13)

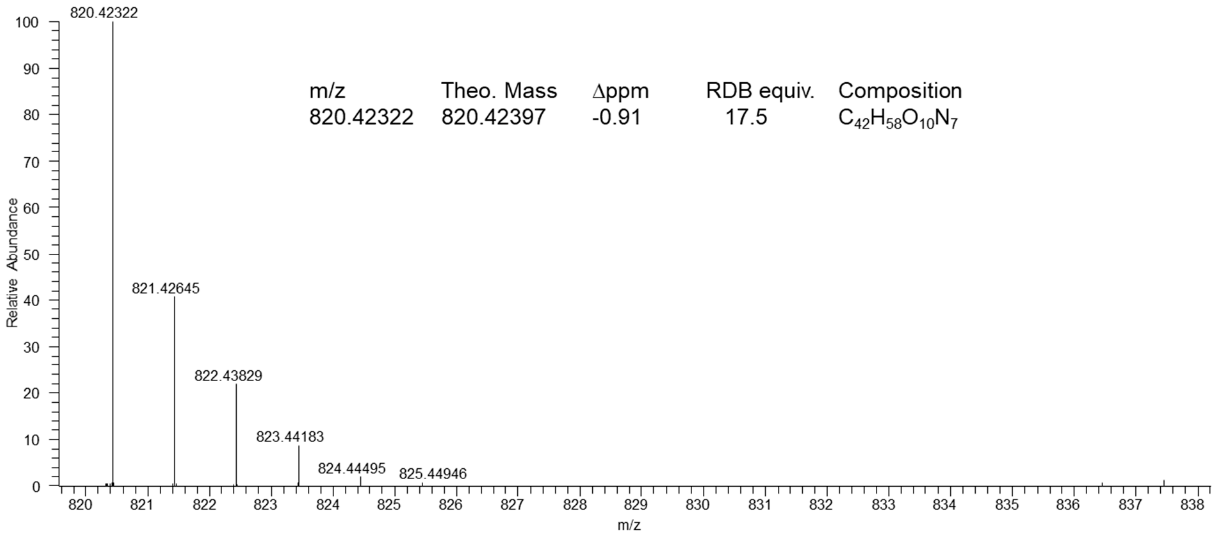

Figure S42. HR-ESI-MS of nobilamide N (**13**), retention time 16.2 min (HPLC method: LC-HR-ESI-MS).

ESI-MS/MS of nobilamide N (13)

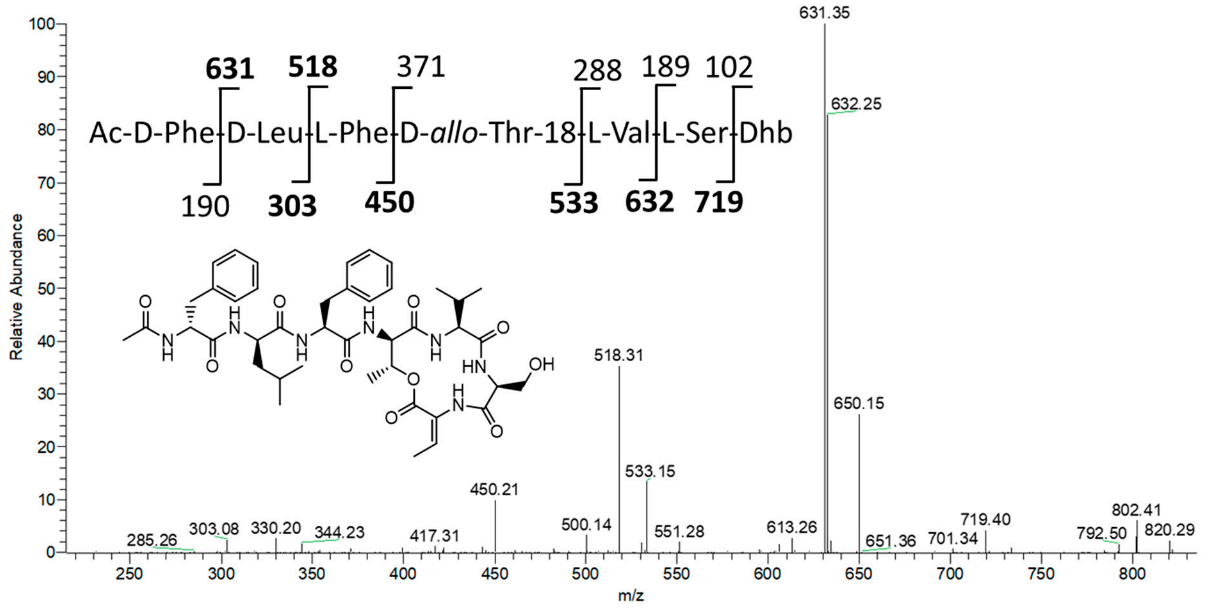

Figure S43. ESI-MS/MS of nobilamide N (**13**). MS/MS of the quasimolecular ion $[M+H]^+$ *m/z* 820.4 of nobilamide N (**13**). The structure of **13** and the y and b ions series fragments after initial ring opening of the ester in the mass spectrometer (D-*allo*-Thr-18) are presented. The observed fragments are highlighted in bold. Dhb: *Z*-α,β-dehydrobutyrine.

Stereochemistry of the amino acids of nobilamide N (13)

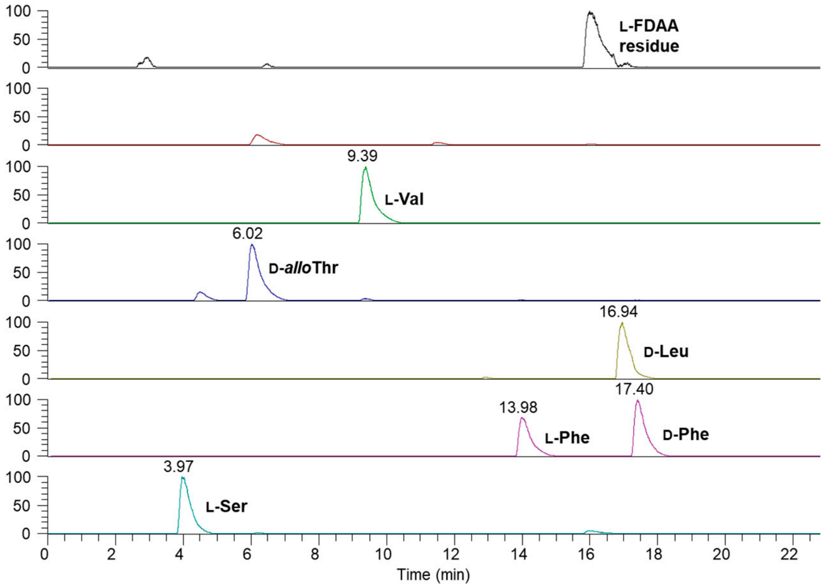

Figure S44. Stereochemistry of the amino acids of nobilamide N (**13**). The stereochemistry of the amino acids of nobilamide N (**13**) was analysed by LC-MS after acid hydrolysis of nobilamide N (**13**) and derivatization with Marfey's reagent **[37,38]**.

Nobilamide I (8)

HR-ESI-MS of nobilamide I (8)

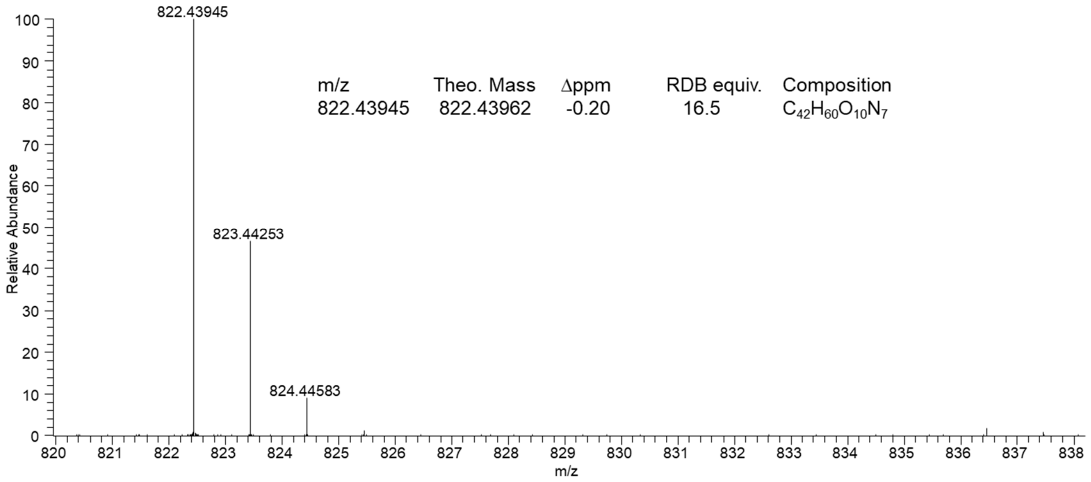

Figure S45. HR-ESI-MS of nobilamide I (**8**), retention time: 15.9 min (HPLC method: LC-HR-ESI-MS).

ESI-MS/MS of nobilamide I (8)

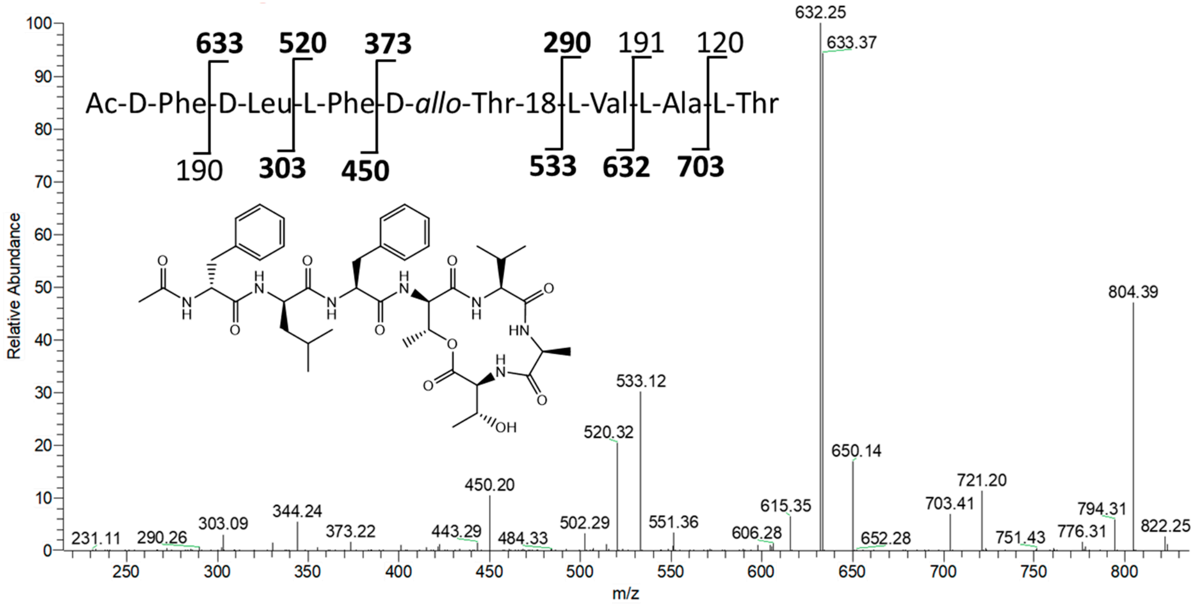

Figure S46. ESI-MS/MS of nobilamide I (**8**). MS/MS of the quasimolecular ion *m/z* 822.4 of nobilamide I (**8**). The structure of **8** and the y and b ions series fragments after initial ring opening (D-*allo*Thr-18) of the ester in the mass spectrometer are presented. The observed fragments are highlighted in bold.

Stereochemistry of the amino acids of nobilamide I (8)

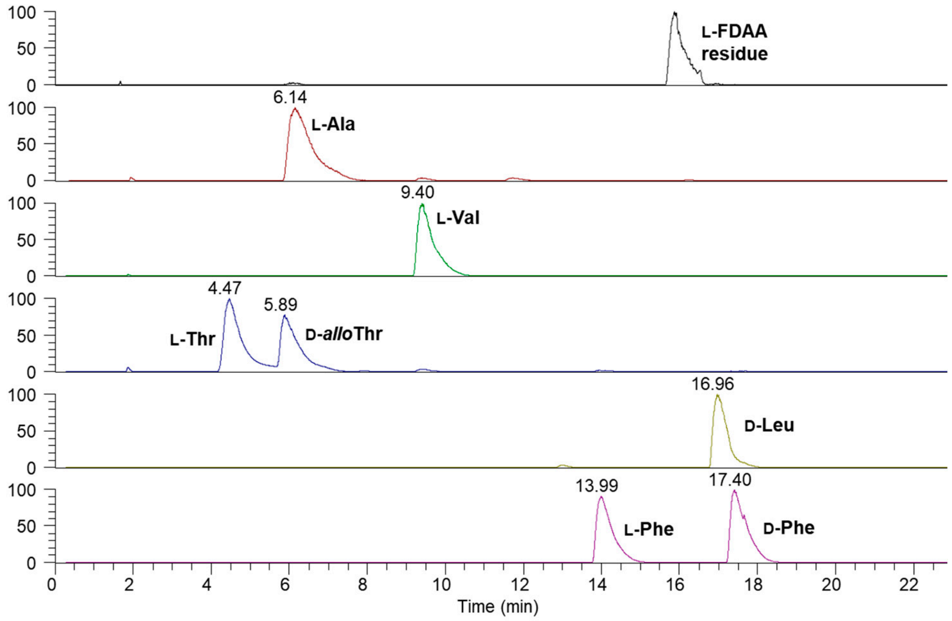

Figure S47. Stereochemistry of the amino acids of nobilamide I (**8**). The stereochemistry of the amino acids of nobilamide I (**8**) was analysed by LC-MS after acid hydrolysis of nobilamide I (**8**) and derivatization with Marfey's reagent **[37,38]**.

^{1}H NMR spectrum of nobilamide I (8)

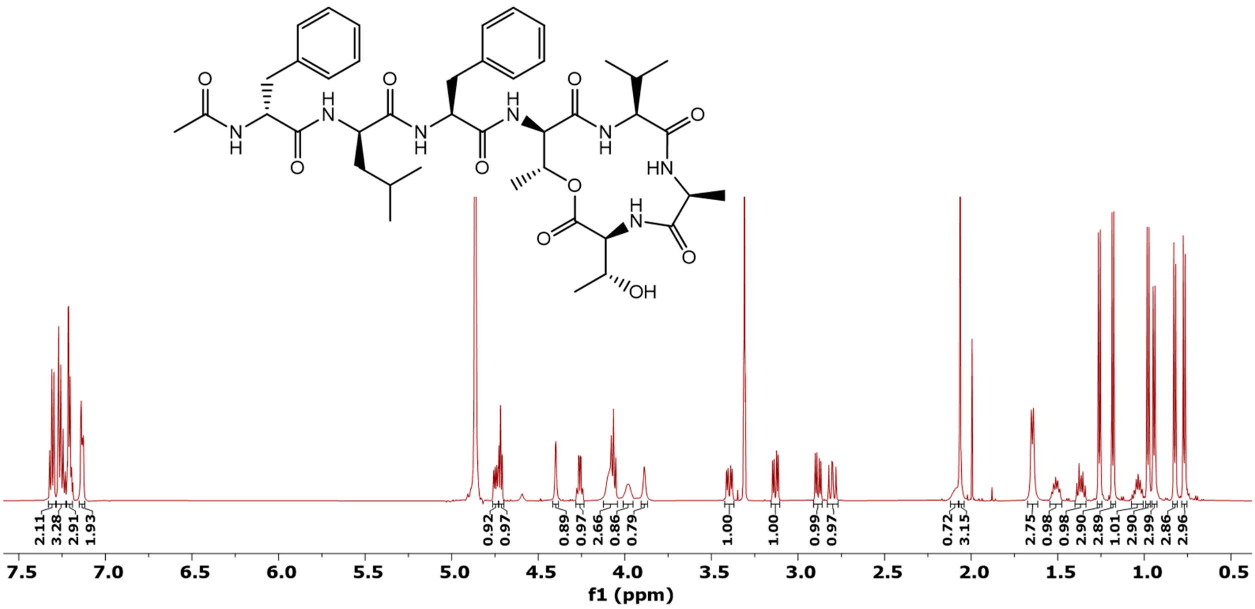

Figure S48. ^{1}H NMR spectrum (600 MHz, CD_3OD) of nobilamide I (**8**).

^{13}C NMR spectrum of nobilamide I (8)

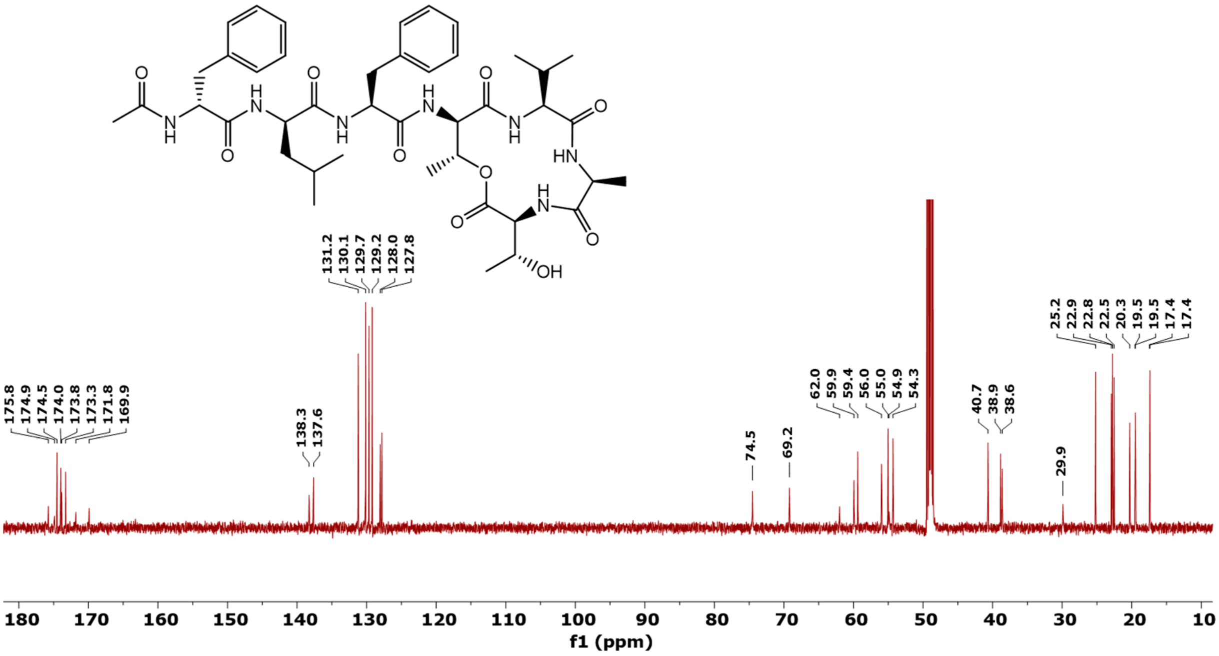

Figure S49. ^{13}C NMR spectrum (151 MHz, CD_3OD) of nobilamide I (**8**).

^{1}H-^{1}H COSY NMR spectrum of nobilamide I (8)

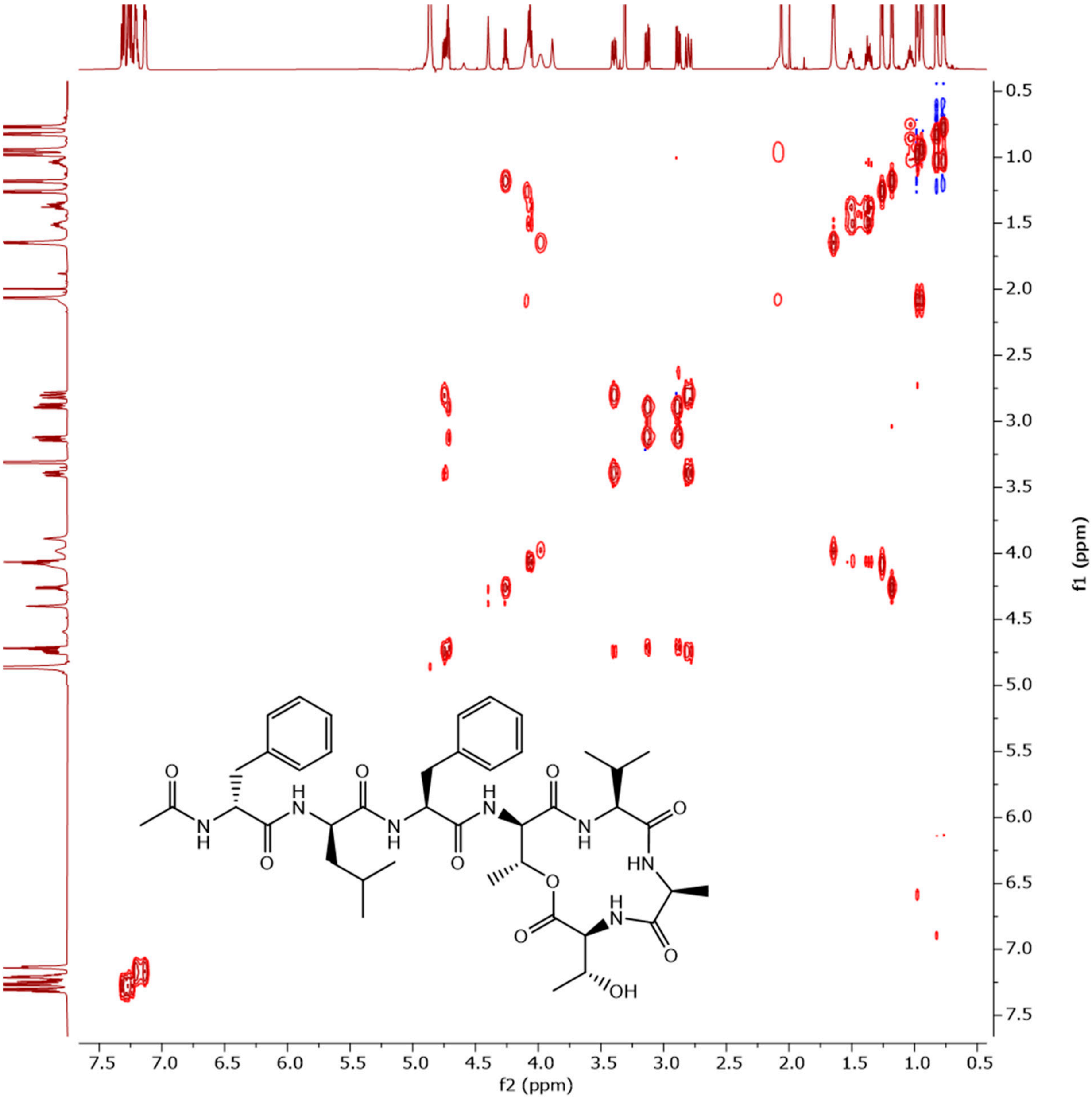

Figure S50. ^{1}H-^{1}H COSY NMR spectrum (600 MHz, CD_3OD) of nobilamide I (**8**).

^{1}H-^{13}C HSQC NMR spectrum (600 MHz, CD_3OD) of nobilamide I (8)

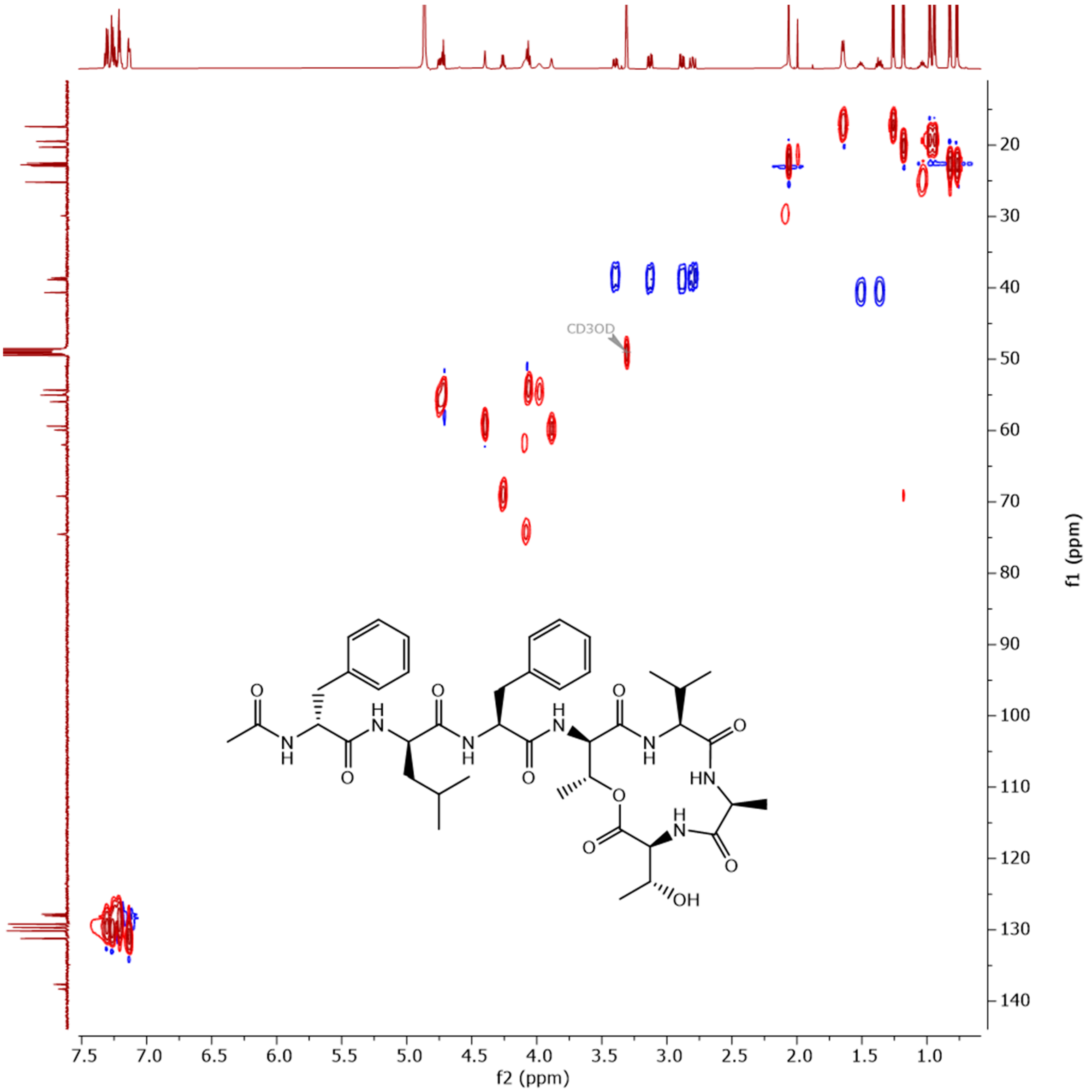

Figure S51. ^{1}H-^{13}C HSQC NMR spectrum (600 MHz, CD_3OD) of nobilamide I (**8**).

^{1}H-^{13}C HMBC NMR spectrum (600 MHz, CD_3OD) of nobilamide I (8)

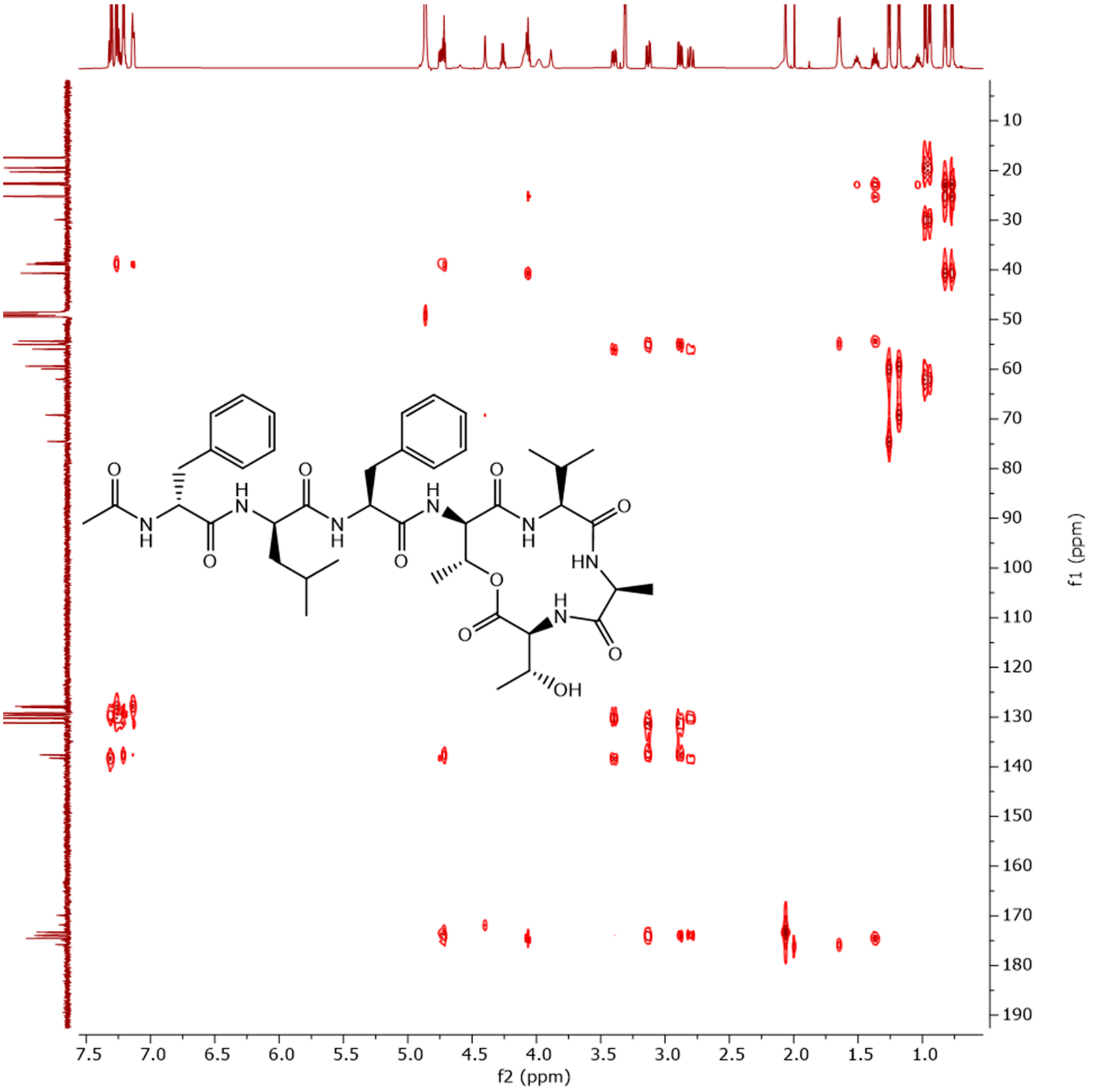

Figure S52. ^{1}H-^{13}C HMBC NMR spectrum (600 MHz, CD_3OD) of nobilamide I (**8**).

Key ^1H-^1H COSY and ^1H-^{13}C HMBC NMR spectrum of nobilamide I (8)

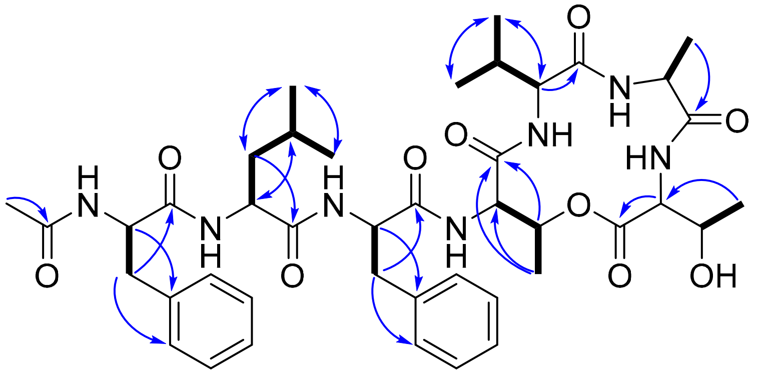

Figure S53. Key ^1H-^1H COSY and ^1H-^{13}C HMBC NMR spectrum (600 MHz, CD_3OD) of nobilamide I (**8**).

NMR data of nobilamide I (8)

2.2 mg, white filmy compound.

^1H-NMR (600 MHz, CD_3OD): δ (ppm): 0.77 (d, *J* = 6.6 Hz, 3H), 0.82 (d, *J* = 6.6 Hz, 3H), 0.94 (d, *J* = 6.6 Hz, 3H), 0.98 (d, *J* = 6.6 Hz, 3H), 1.04 (dq, *J* = 13.9, 6.9 Hz, 1H), 1.18 (d, *J* = 6.4 Hz, 3H), 1.26 (d, *J* = 6.4 Hz, 3H), 1.37 (dt, *J* = 13.4, 7.7 Hz, 1H), 1.51 (dt, *J* = 14.4, 6.9 Hz, 1H), 1.65 (d, *J* = 7.5 Hz, 3H), 2.06 (s, 3H), 2.80 (dd, *J* = 14.0, 10.9 Hz, 1H), 2.88 (dd, *J* = 13.9, 5.8 Hz, 1H), 3.13 (dd, *J* = 13.9, 5.2 Hz, 1H), 3.40 (dd, *J* = 14.0, 5.3 Hz, 1H), 3.89 (s, 1H), 3.98 (s, 1H), 4.04 – 4.12 (m, 3H), 4.26 (qd, *J* = 6.4, 2.1 Hz, 1H), 4.38 – 4.42 (m, 1H), 4.72 (t, *J* = 5.5 Hz, 1H), 4.74 (dd, *J* = 10.8, 5.2 Hz, 1H), 7.12 – 7.15 (m, 2H), 7.19 – 7.22 (m, 3H), 7.23 – 7.28 (m, 3H), 7.29 – 7.33 (m, 2H).

^{13}C-NMR (151 MHz, CD_3OD): δ (ppm). 17.39, 17.44, 19.48, 19.51, 20.28, 22.54, 22.76, 22.92, 25.18, 29.90, 38.62, 38.86, 40.67, 54.33, 54.88, 55.03, 55.97, 59.38, 59.93, 62.00, 69.21, 74.52, 127.80, 128.04, 129.19, 129.65, 130.14, 131.21, 137.62, 138.28, 169.91, 171.81, 173.28, 173.84, 173.97, 174.51, 175.77.

Nobilamide O (14) and Nobilamide P (15)

HR-ESI-MS of nobilamide O (14) and nobilamide P (15)

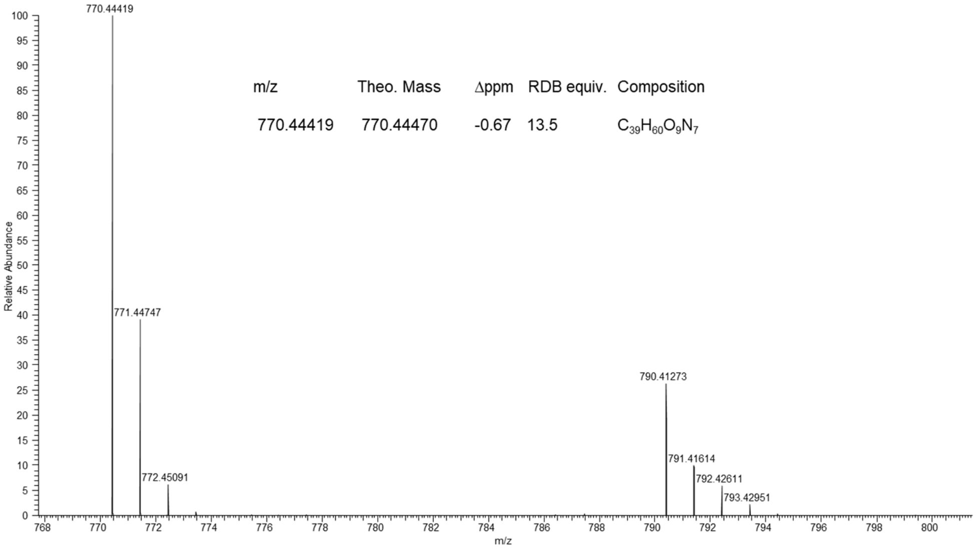

Figure S54. HR-ESI-MS of nobilamide O (**9**) and nobilamide P (**10**), retention time: 18.9 min and 18.6 min respectively (HPLC method: LC-HR-ESI-MS).

ESI-MS/MS of nobilamide O (14) and nobilamide P (15)

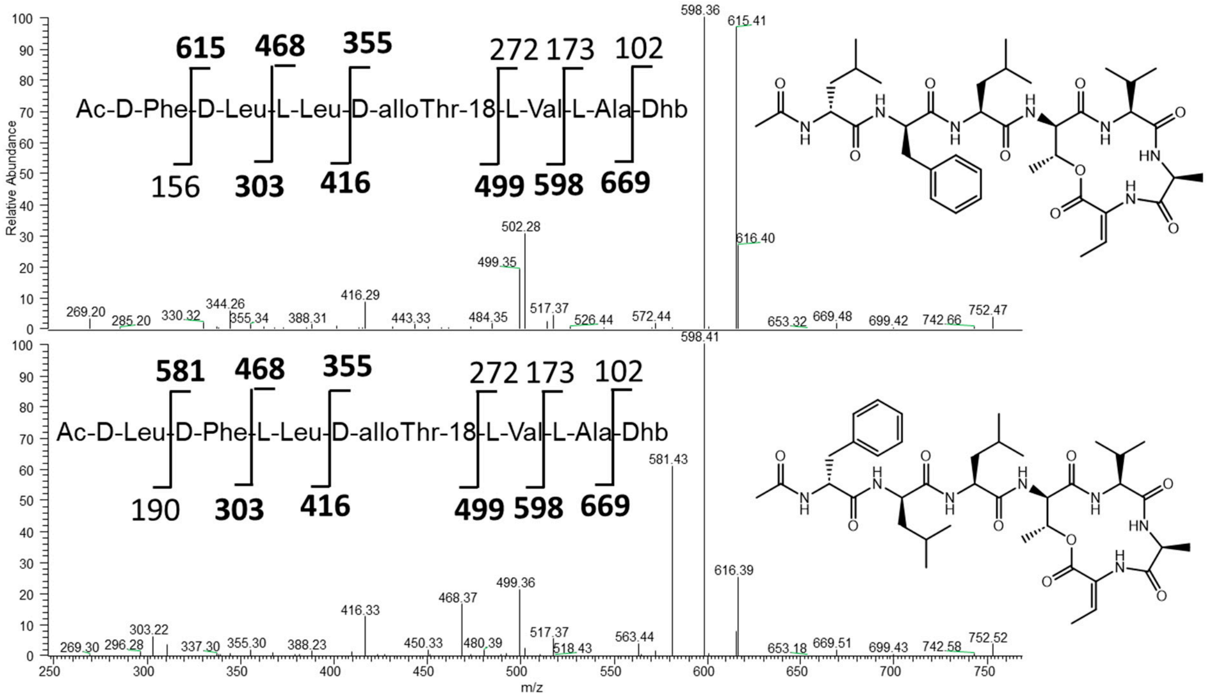

Figure S55. ESI-MS/MS of nobilamide O (**14,** below) and nobilamide P (**15,** above). MS/MS of the quasimolecular ion $[M+H]^+$ *m/z* 770.4 of nobilamide O (**14**) and nobilamide P (**15**). The structures of **14** and **15** and their y and b ions series fragments after initial ring opening (D-*allo*-Thr-18) of the ester in the mass spectrometer are presented. The observed fragments are highlighted in bold. Dhb: *Z*-α,β-dehydrobutyrine.

Stereochemistry of the amino acids of the mixture of nobilamide O (14) and nobilamide P (15)

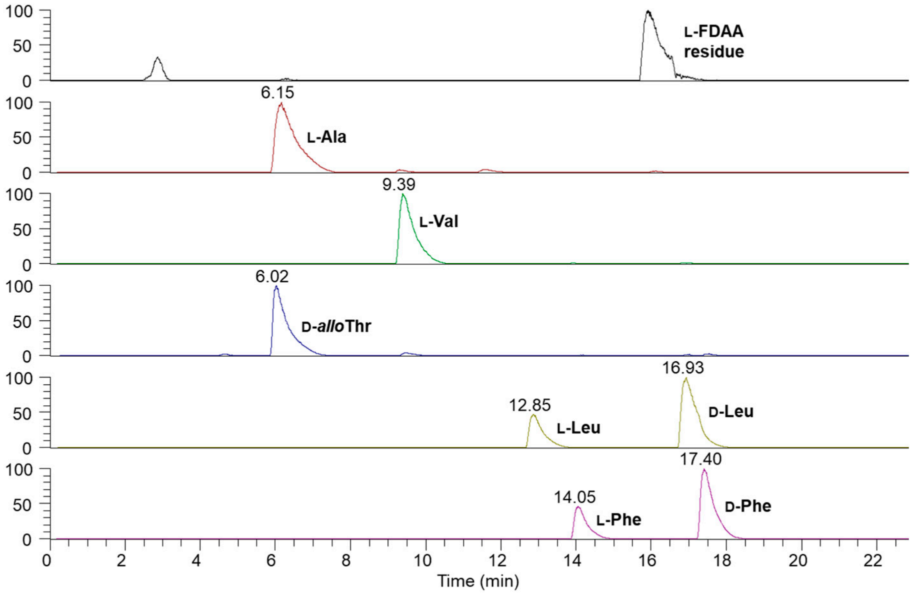

Figure S56. Stereochemistry of the amino acids of the mixture of nobilamide O (**14**) and nobilamide P (**15**). The stereochemistry of the amino acids of the mixture nobilamide O (**14**) and nobilamide P (**15**) was analysed by LC-MS after acid hydrolysis of nobilamide O (**14**) and nobilamide P (**15**) and derivatization with Marfey's reagent **[37,38**].

The tentative stereochemistry of the peptides, nobilamide O (**14**) and nobilamide P (**15**), is presented based on the Marfey analysis and the established stereochemistry of amino acids at the respective positions in the peptide sequence.

Nobilamide Q (16)

HR-ESI-MS of nobilamide Q (16))

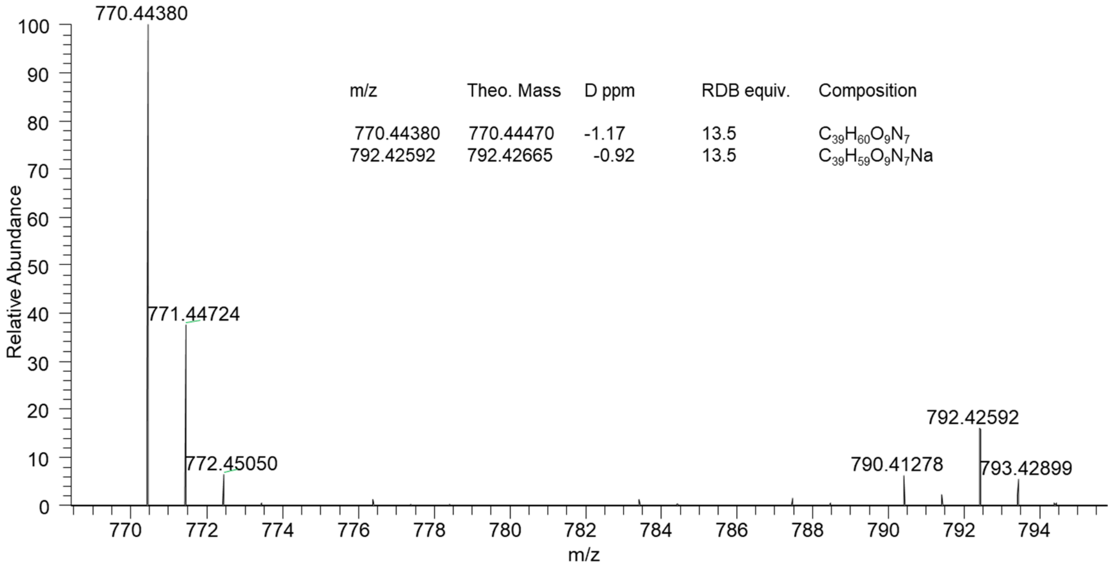

Figure S57. HR-ESI-MS of nobilamide Q (**16**), retention time: 20.1 min (HPLC method: LC-HR-ESI-MS).

ESI-MS/MS of nobilamide Q (16)

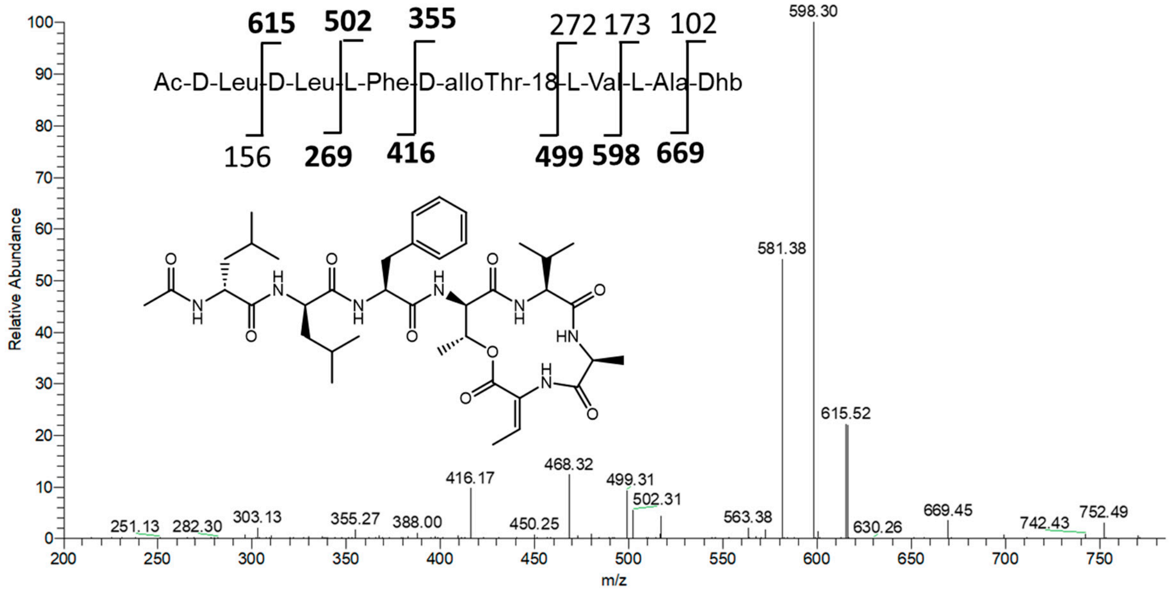

Figure S58. ESI-MS/MS of nobilamide Q (**16**). MS/MS of the quasimolecular ion *m/z* 770.4 of nobilamide Q (**16**). The structure of **16**, and the y and b fragments of **16** after initial ring opening of the ester (D-*allo*-Thr-18) are presented. Observed fragments are highlighted in bold. Dhb: *Z*-α,β-dehydrobutyrine.

Stereochemistry of the amino acids of nobilamide Q (16)

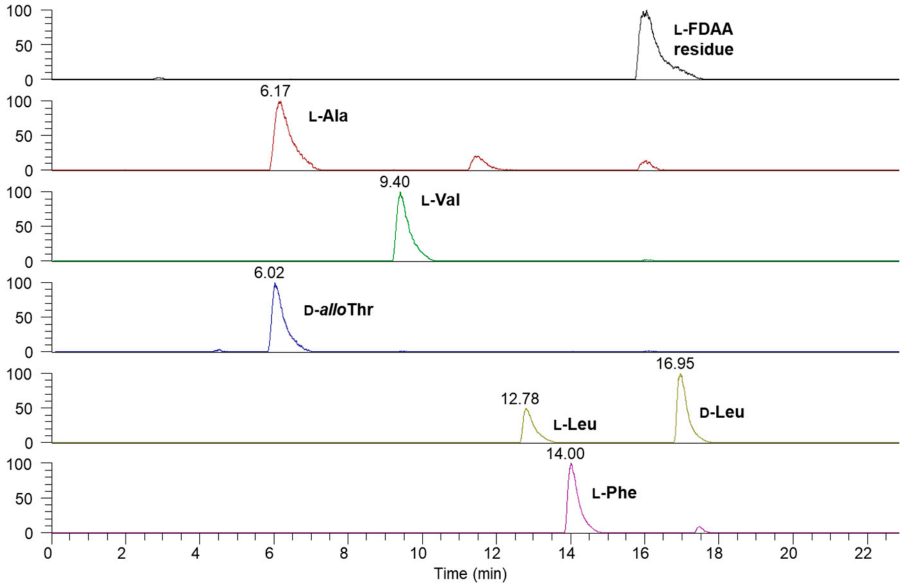

Figure S59. Stereochemistry of the amino acids of nobilamide Q (**16**). The stereochemistry of the amino acids of nobilamide Q (**16**) was analysed by LC-MS after acid hydrolysis of nobilamide Q (**16**) and derivatization with Marfey's reagent **[37,38]**.

The structure of nobilamide Q (**16**) is given with the most likely stereochemistry deduced in analogy to A-3302-B (**1**) assuming that the stereochemistry of the amino acids is maintained also after exchange of the amino acids.

HR-ESI-MS of nobilamide R (17)

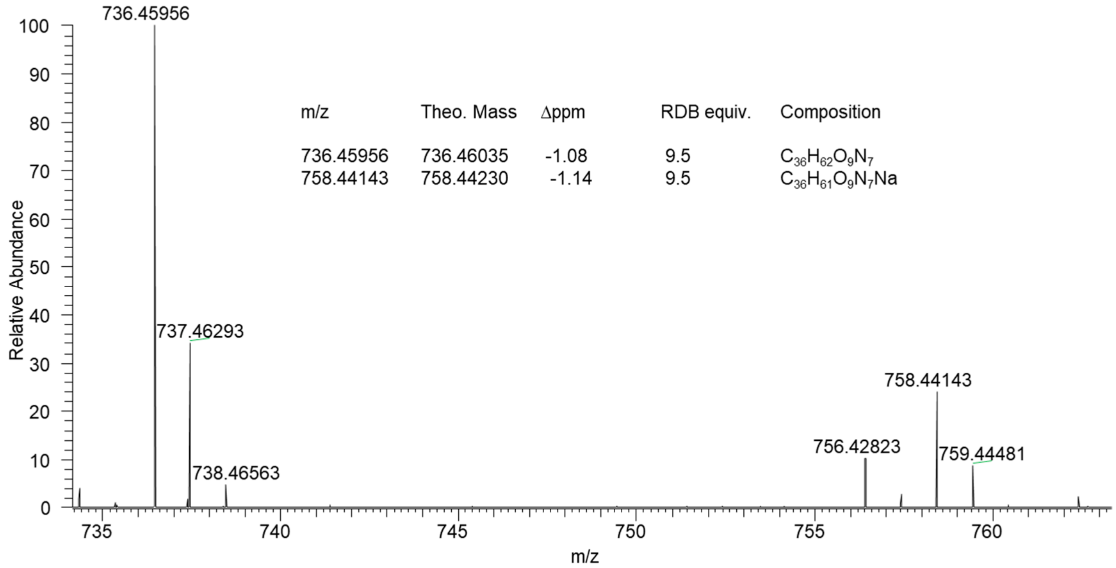

Figure S60. HR-ESI-MS of nobilamide R (**17**), retention time: 17.9 min (HPLC method: LC-HR-ESI-MS).

ESI-MS/MS of nobilamide R (17)

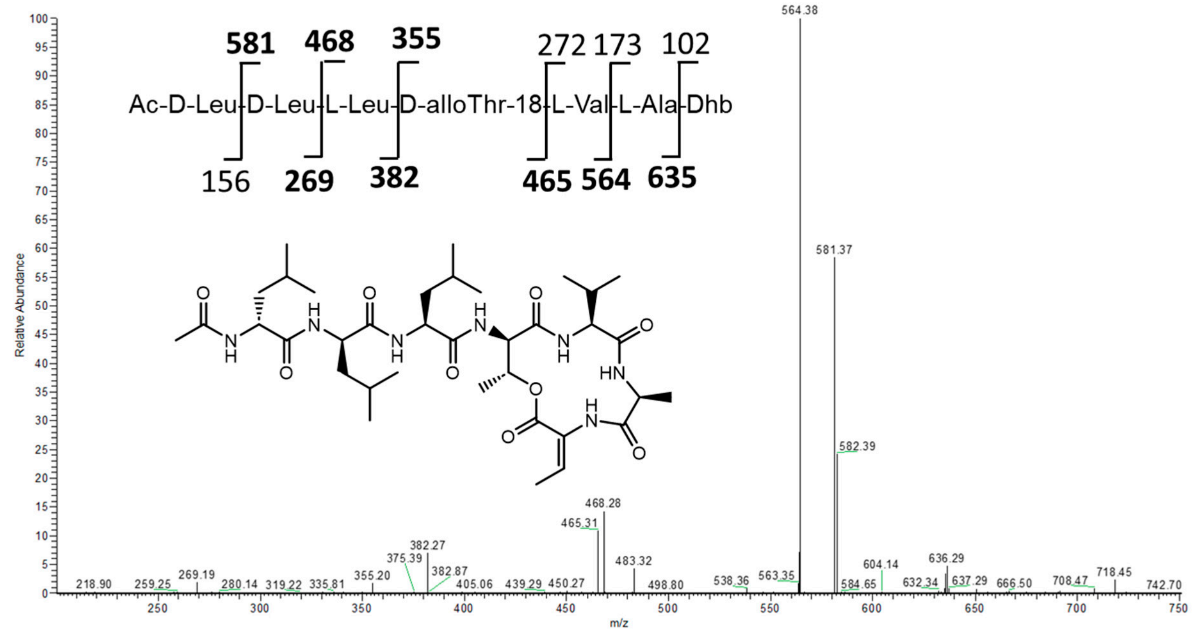

Figure S61. ESI-MS/MS of nobilamide R (**17**). MS/MS of the quasimolecular ion $[M+H]^+$ *m/z* 736.4 of nobilamide R (**17**). The structure of **17** and the y and b fragments after initial ring opening of the ester (D-alloThr-18) in the mass spectrometer are presented. The observed fragments are highlighted in bold. Dhb: *Z*-α,β-dehydrobutyrine.

Stereochemistry of the amino acids of nobilamide R (17)

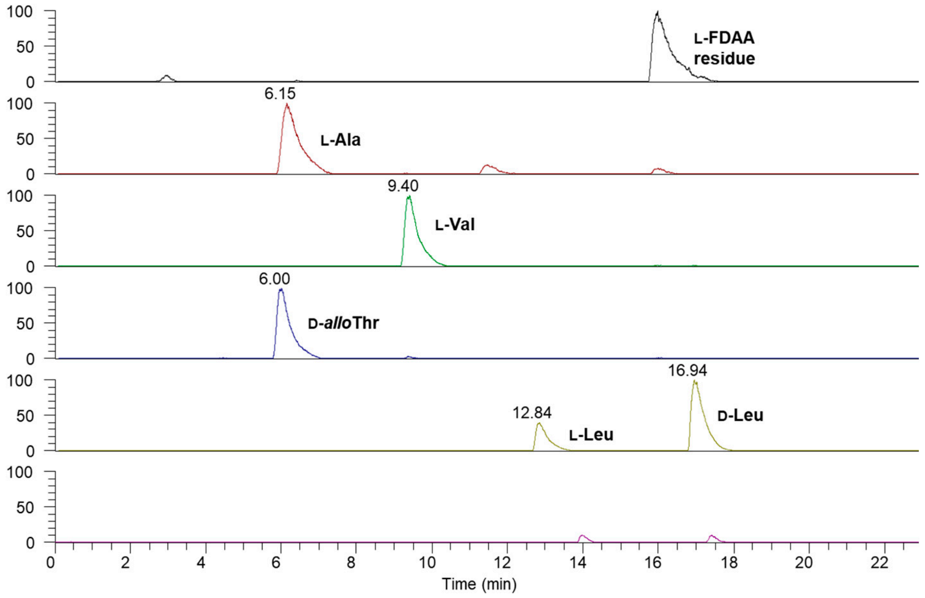

Figure S62. Stereochemistry of the amino acids of nobilamide R (**17**). The stereochemistry of the amino acids of nobilamide R (**17**) was analysed by LC-MS after acid hydrolysis of nobilamide R (**17**) and derivatization with Marfey's reagent **[37,38]**.

Nobilamide A (4)

HR-ESI-MS of nobilamide A (4)

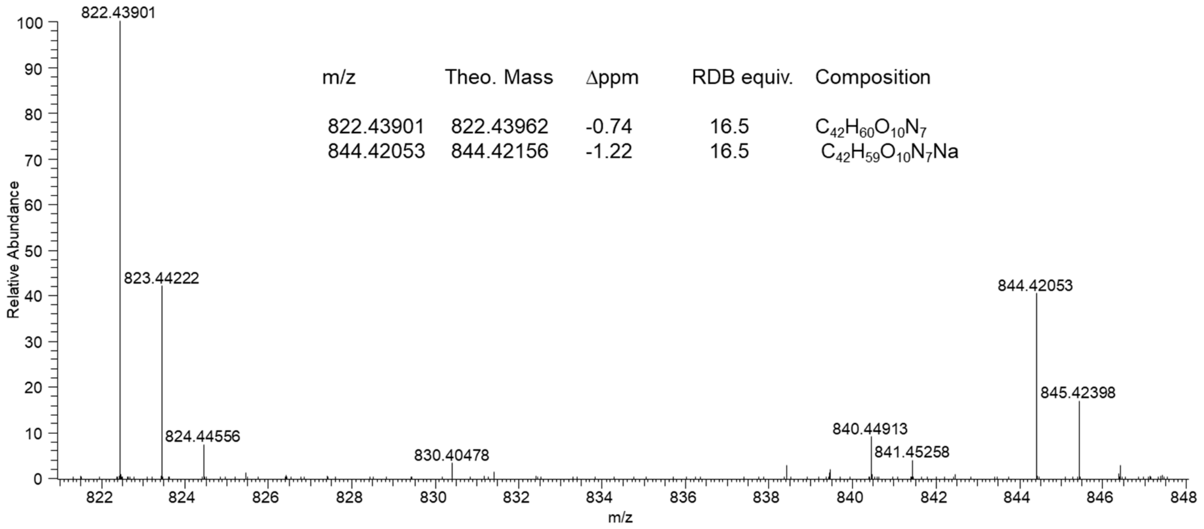

Figure S63. HR-ESI-MS of nobilamide A (**4**), retention time: 14.7 min (HPLC method: LC-HR-ESI-MS).

ESI-MS/MS of nobilamide A (4)

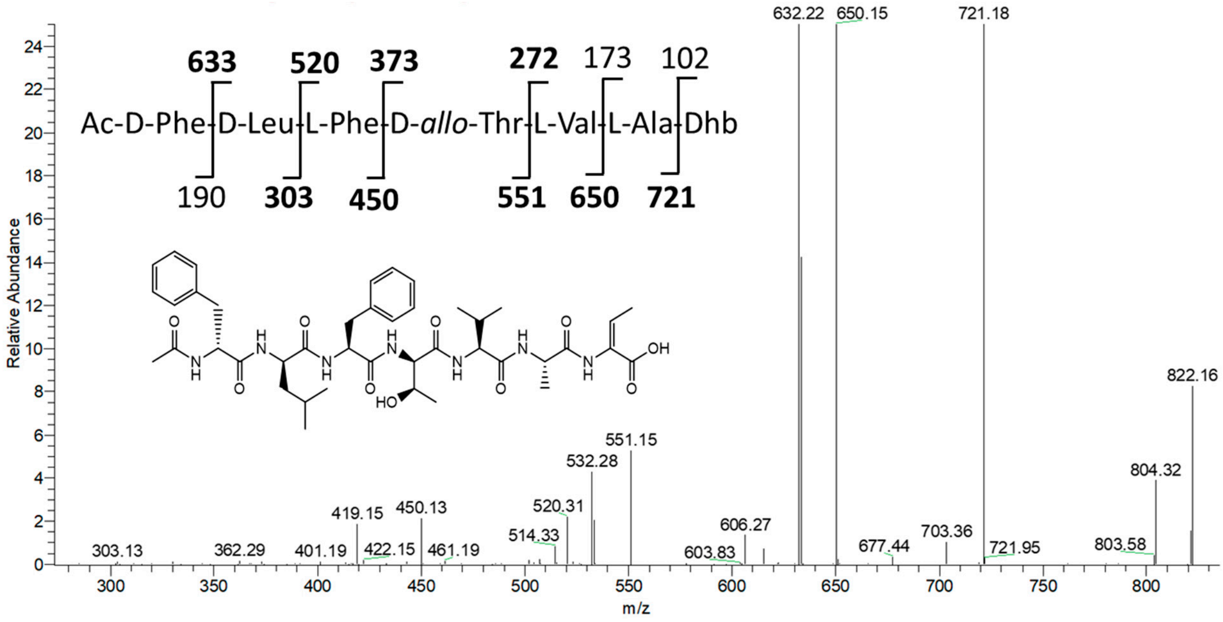

Figure S64. ESI-MS/MS of nobilamide A (**4**). MS/MS of the quasimolecular ion $[M+H]^+$ *m/z* 822.2 of nobilamide A (**4**). The structure of **4** and the y and b ion fragments of **4** are presented. The observed fragments are highlighted in bold. Dhb: *Z*-α,β-dehydrobutyrine.

Stereochemistry of the amino acids of nobilamide A (4)

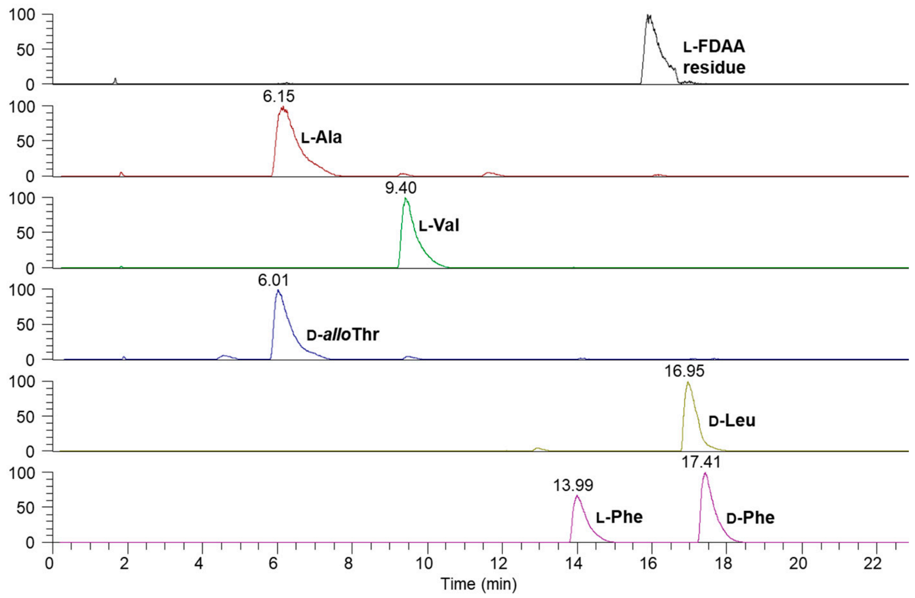

Figure S65. Stereochemistry of the amino acids of nobilamide A (**4**). The stereochemistry of the amino acids of nobilamide A (**4**) was analysed by LC-MS after acid hydrolysis of nobilamide A (**4**) and derivatization with Marfey's reagent **[37,38]**.

^{1}H NMR spectrum (600 MHz, CD_3OD) of nobilamide A (4)

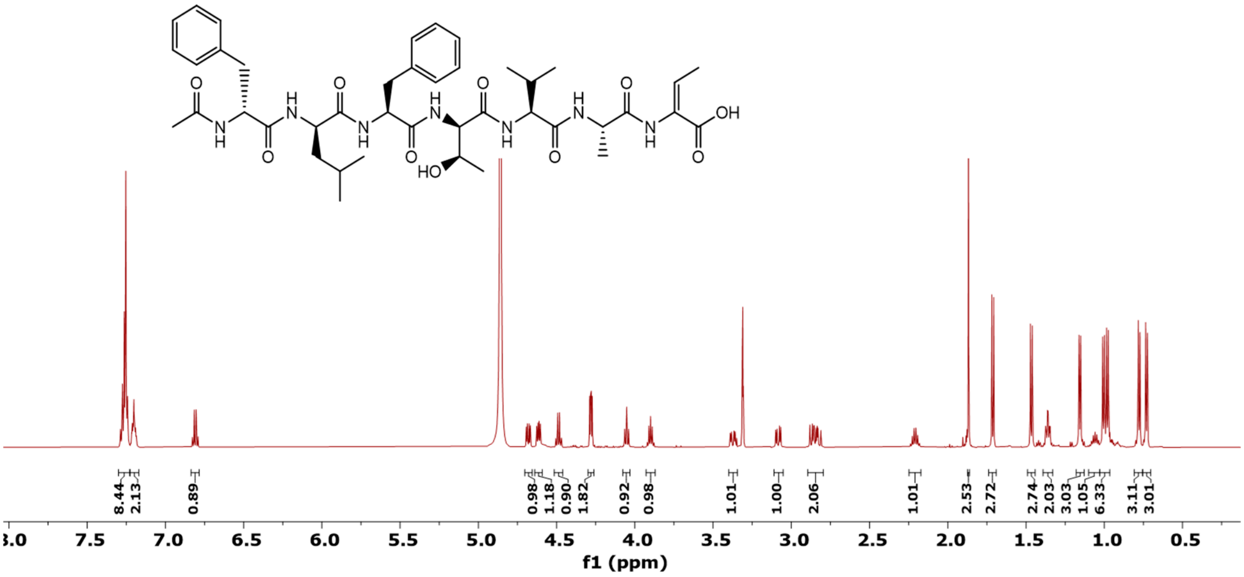

Figure S66. ^{1}H NMR spectrum (600 MHz, CD_3OD) of nobilamide A (**4**).

^{13}C NMR spectrum (151 MHz, CD_3OD) of nobilamide A (4)

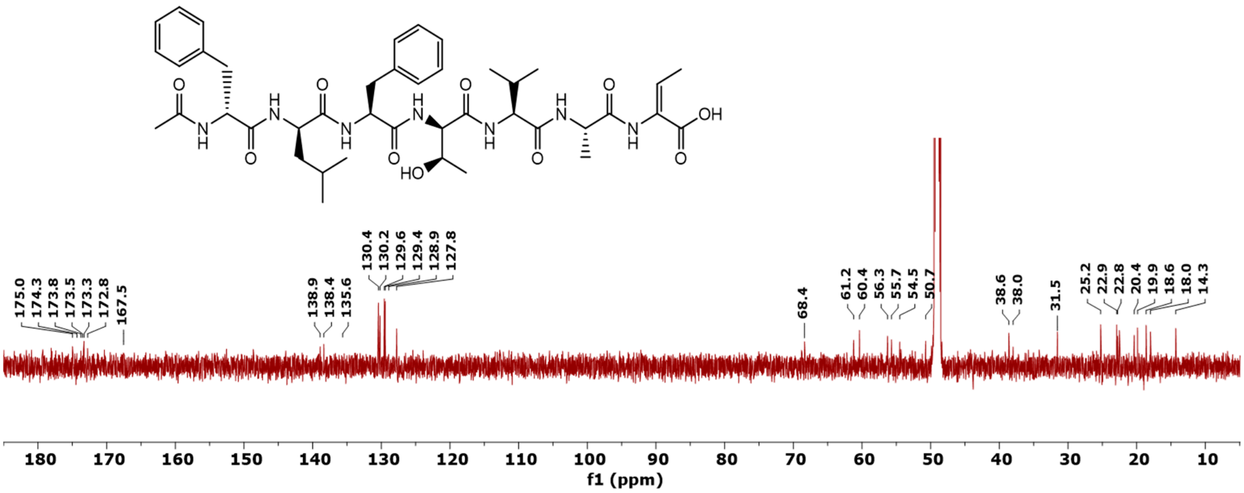

Figure S67. ^{13}C NMR spectrum (151 MHz, CD_3OD) of nobilamide A (**4**).

^1H-^1H COSY NMR spectrum (600 MHz, CD_3OD) of nobilamide A (4)

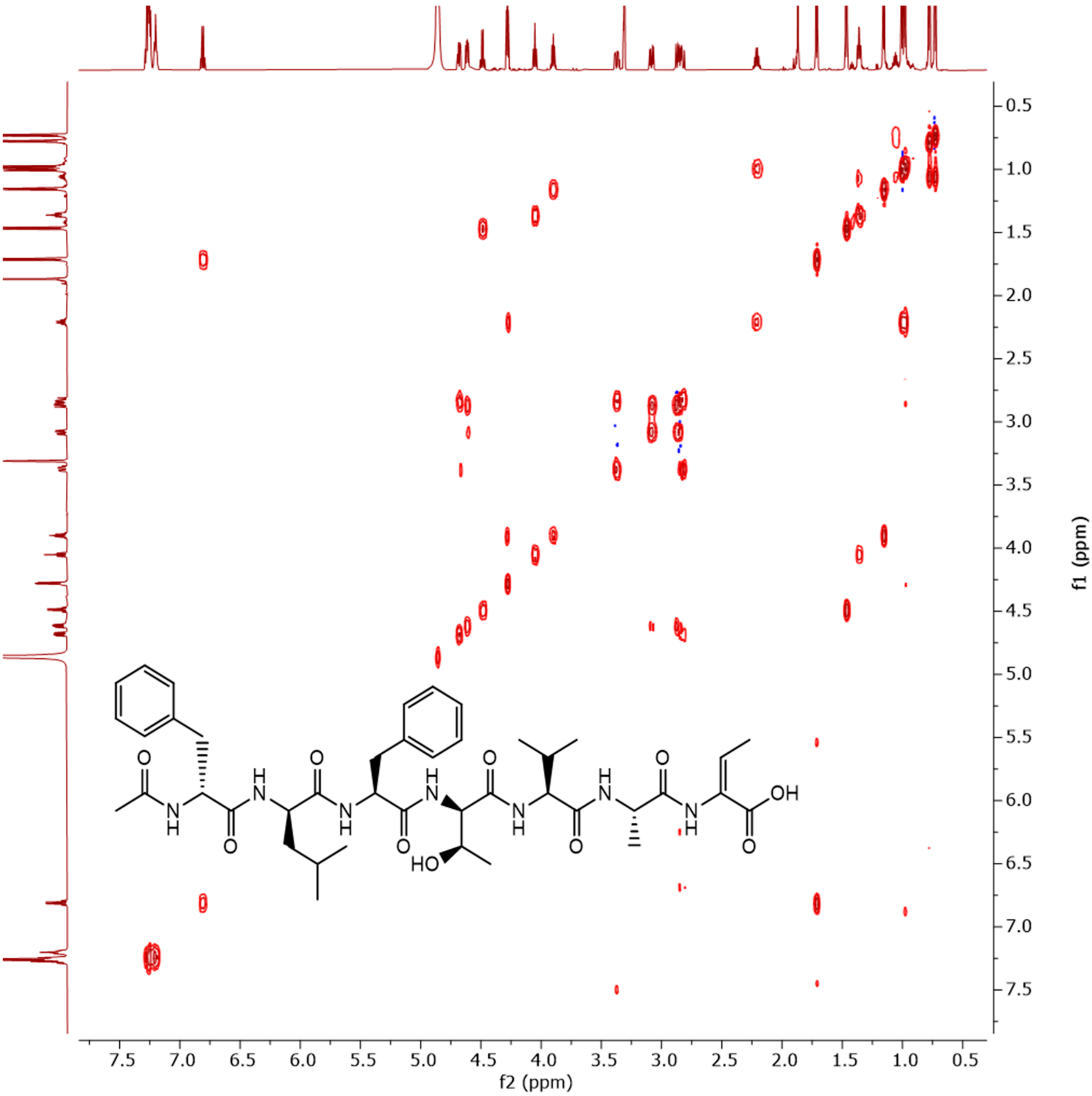

Figure S68. ^1H-^1H COSY NMR spectrum (600 MHz, CD_3OD) of nobilamide A (**4**).

^{1}H-^{13}C HSQC NMR spectrum of nobilamide A (4)

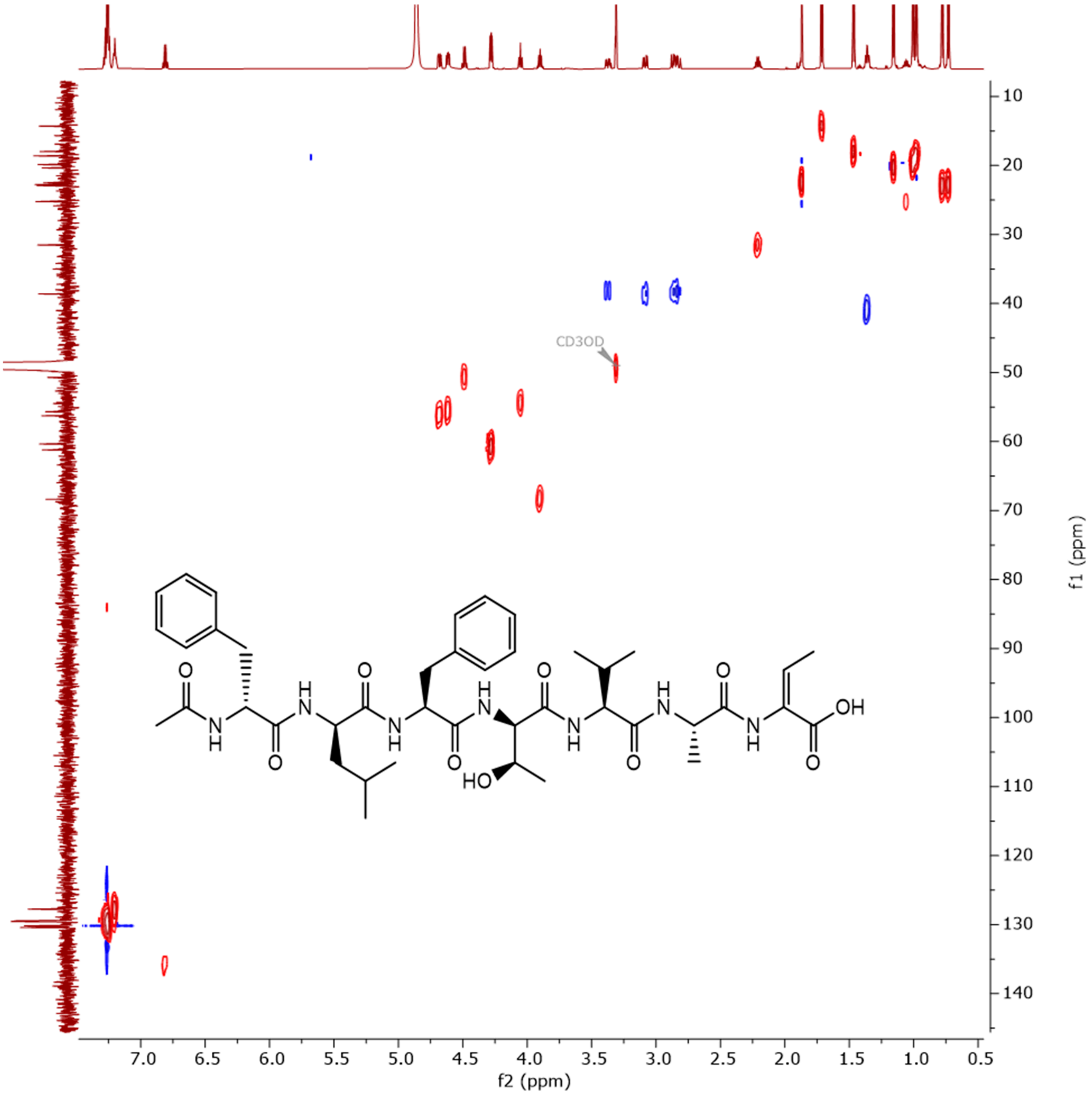

Figure S69. ^{1}H-^{13}C HSQC NMR spectrum (600 MHz, CD_3OD) of nobilamide A (**4**).

^{1}H-^{13}C HMBC NMR spectrum of nobilamide A (4)

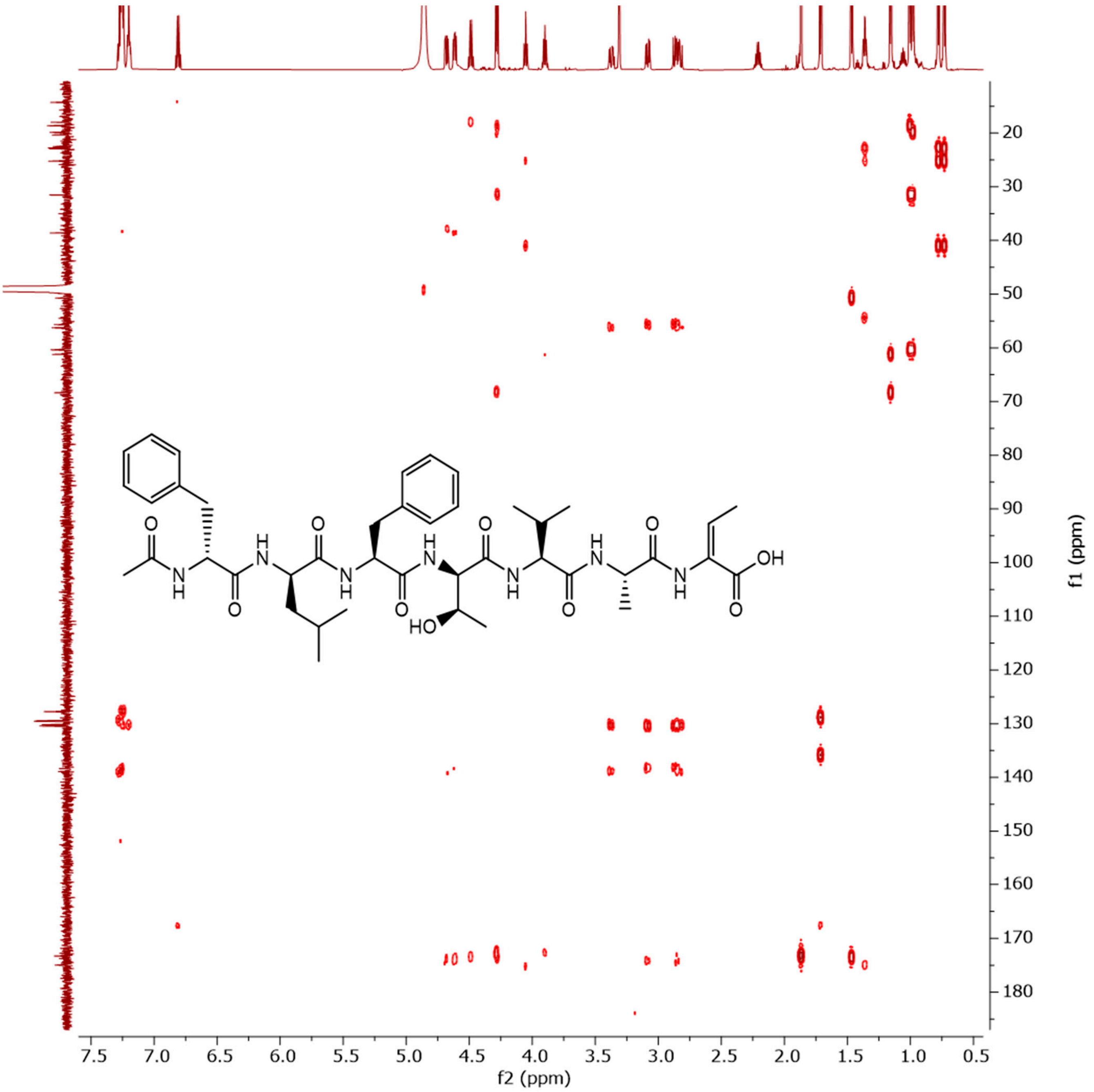

Figure S70. ^{1}H-^{13}C HMBC NMR spectrum (600 MHz, CD_3OD) of nobilamide A (**4**).

Key ^{1}H-^{1}H COSY and ^{1}H-^{13}C HMBC NMR spectrum of nobilamide A (4)

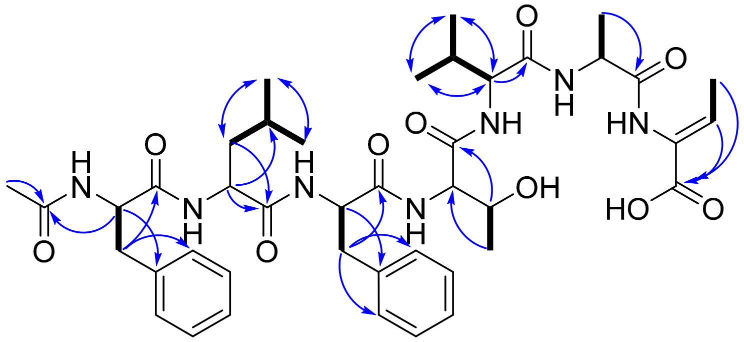

Figure S71. Key ^{1}H-^{1}H COSY and ^{1}H-^{13}C HMBC NMR spectrum (600 MHz, CD_3OD) of nobilamide A (**4**).

NMR data of nobilamide A (4)

4 mg, white amorphous powder.

^{1}H-NMR (600 MHz, CD_3OD): δ (ppm): 0.73 (d, *J* = 6.6 Hz, 3H), 0.78 (d, *J* = 6.5 Hz, 3H), 0.98 (d, *J* = 6.8 Hz, 3H), 1.00 (d, *J* = 6.9 Hz, 3H), 1.06 (hept, *J* = 6.5 Hz, 1H), 1.16 (d, *J* = 6.4 Hz, 3H), 1.36 (td, *J* = 7.4, 2.5 Hz, 2H), 1.47 (d, *J* = 7.2 Hz, 3H), 1.71 (d, *J* = 7.2 Hz, 3H), 1.87 (s, 3H), 2.21 (h, *J* = 6.8 Hz, 1H), 2.83 (dd, *J* = 14.3, 11.1 Hz, 1H), 2.86 (dd, *J* = 14.1, 9.3 Hz, 1H), 3.08 (dd, *J* = 14.1, 4.9 Hz, 1H), 3.37 (dd, *J* = 14.4, 4.8 Hz, 1H), 3.90 (p, *J* = 6.4 Hz, 1H), 4.05 (t, *J* = 7.6 Hz, 1H), 4.28 (d, *J* = 6.6 Hz, 1H), 4.28 (d, *J* = 6.1 Hz, 1H), 4.49 (q, *J* = 7.2 Hz, 1H), 4.62 (dd, *J* = 9.3, 4.9 Hz, 1H), 4.68 (dd, *J* = 11.2, 4.5 Hz, 1H), 6.81 (q, *J* = 7.1 Hz, 1H), 7.17 – 7.23 (m, 2H), 7.23 – 7.29 (m, 8H).

^{13}C-NMR (151 MHz, CD_3OD): δ (ppm): 14.30, 17.99, 18.62, 19.87, 20.35, 22.77, 22.92, 25.24, 31.54, 37.99, 38.61, 50.72, 54.51, 55.69, 56.27, 60.36, 61.21, 68.38, 127.76, 128.91, 129.44, 129.56, 130.18, 130.42, 135.61, 138.36, 138.91, 167.53, 172.77, 173.29, 173.32, 173.52, 173.76, 174.32, 174.97.

Nobilamide S (18)

HR-ESI-MS of nobilamide S (18)

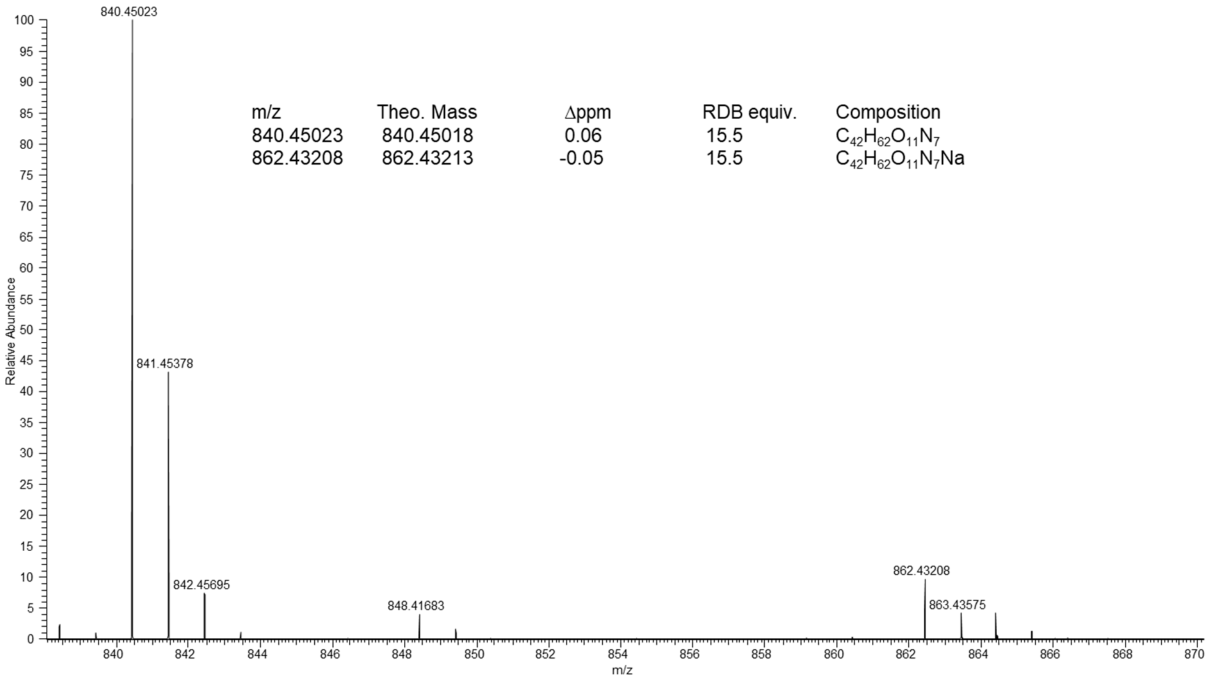

Figure S72. HR-ESI-MS of nobilamide S (**18**), retention time: 14.5 min (HPLC method: LC-HR-ESI-MS).

ESI-MS/MS of nobilamide S (18)

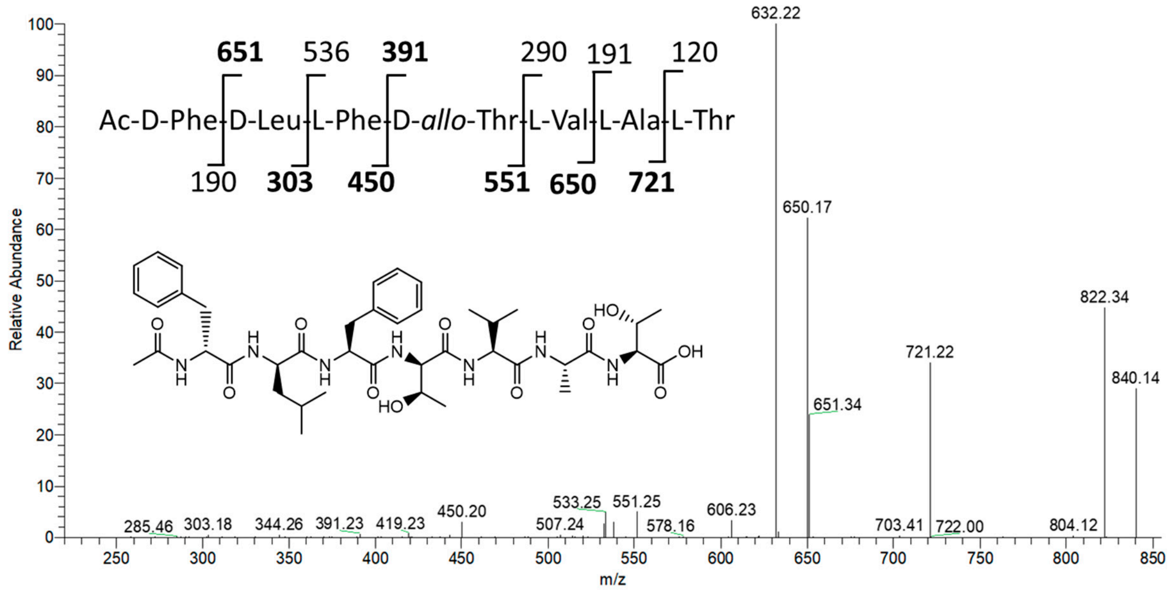

Figure S73. ESI-MS/MS of nobilamide S (**18**). MS/MS of the quasimolecular ion $[M+H]^+$ *m/z* 840.4 of nobilamide S (**18**). The structure of **18** and the y and b ion fragments of **18** are presented. The observed fragments are highlighted in bold.

Stereochemistry of the amino acids of nobilamide S (18)

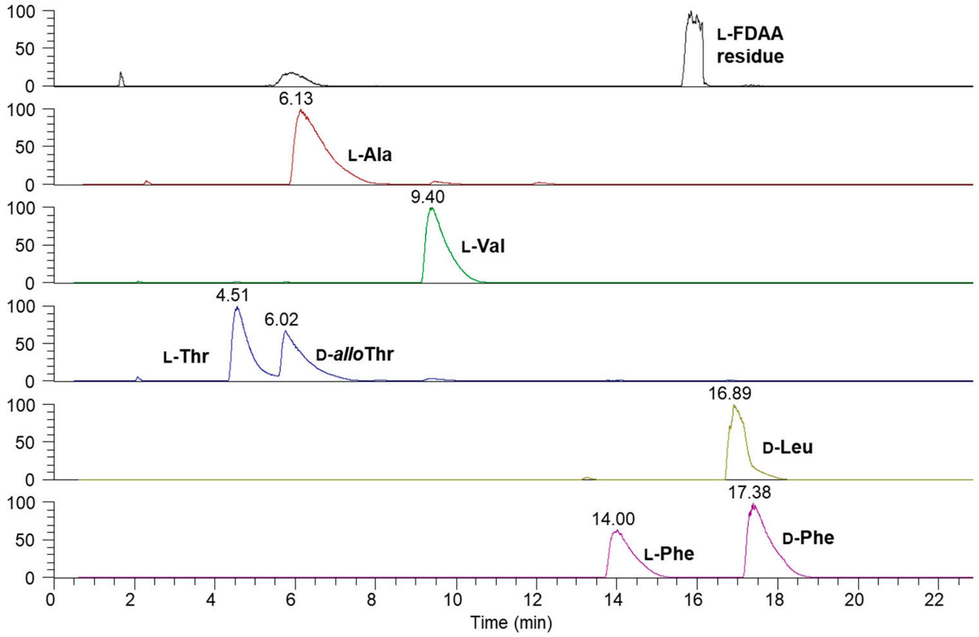

Figure S74. Stereochemistry of the amino acids of nobilamide S (**18**). The stereochemistry of the amino acids of nobilamide S (**18**) was analysed by LC-MS after acid hydrolysis of nobilamide S (**18**) and derivatization with Marfey's reagent **[37,38]**.

^{1}H NMR spectrum of nobilamide S (18)

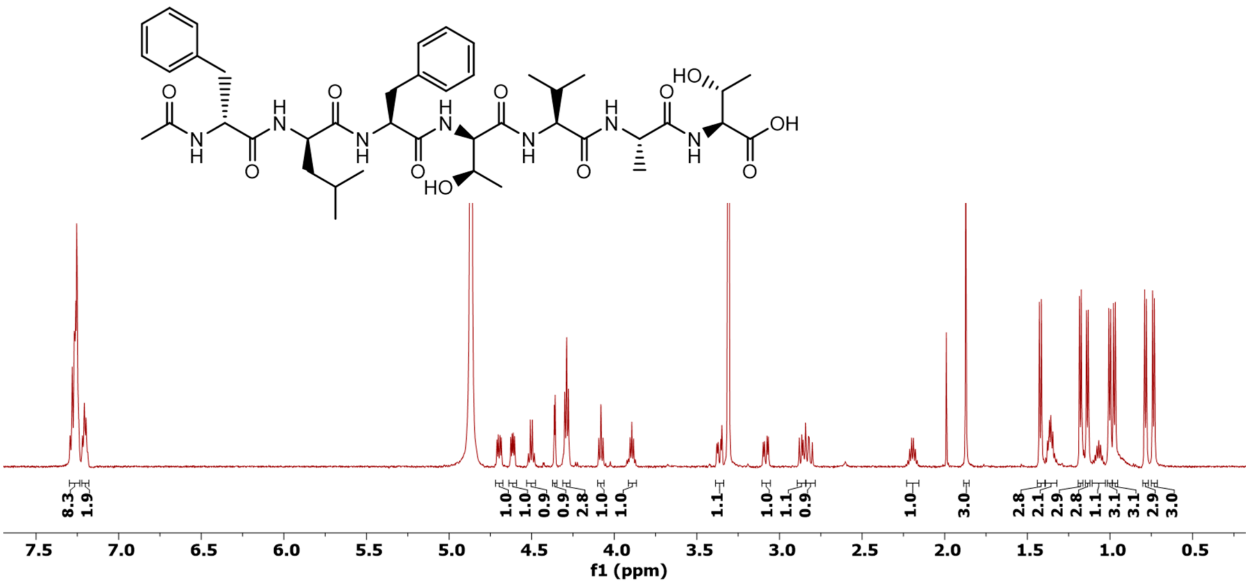

Figure S75. ^{1}H NMR spectrum (600 MHz, CD_3OD) of nobilamide S (**18**).

^{13}C NMR spectrum of nobilamide S (18)

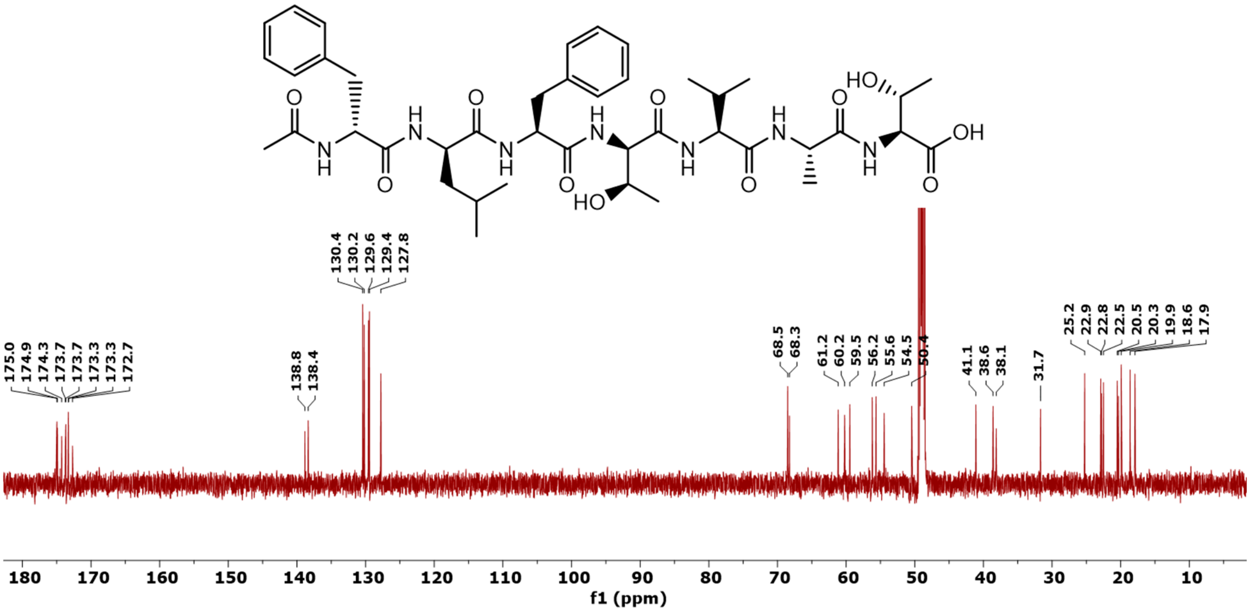

Figure S76. ^{13}C NMR spectrum (151 MHz, CD_3OD) of nobilamide S (**18**).

^{1}H-^{1}H COSY NMR spectrum of nobilamide S (18)

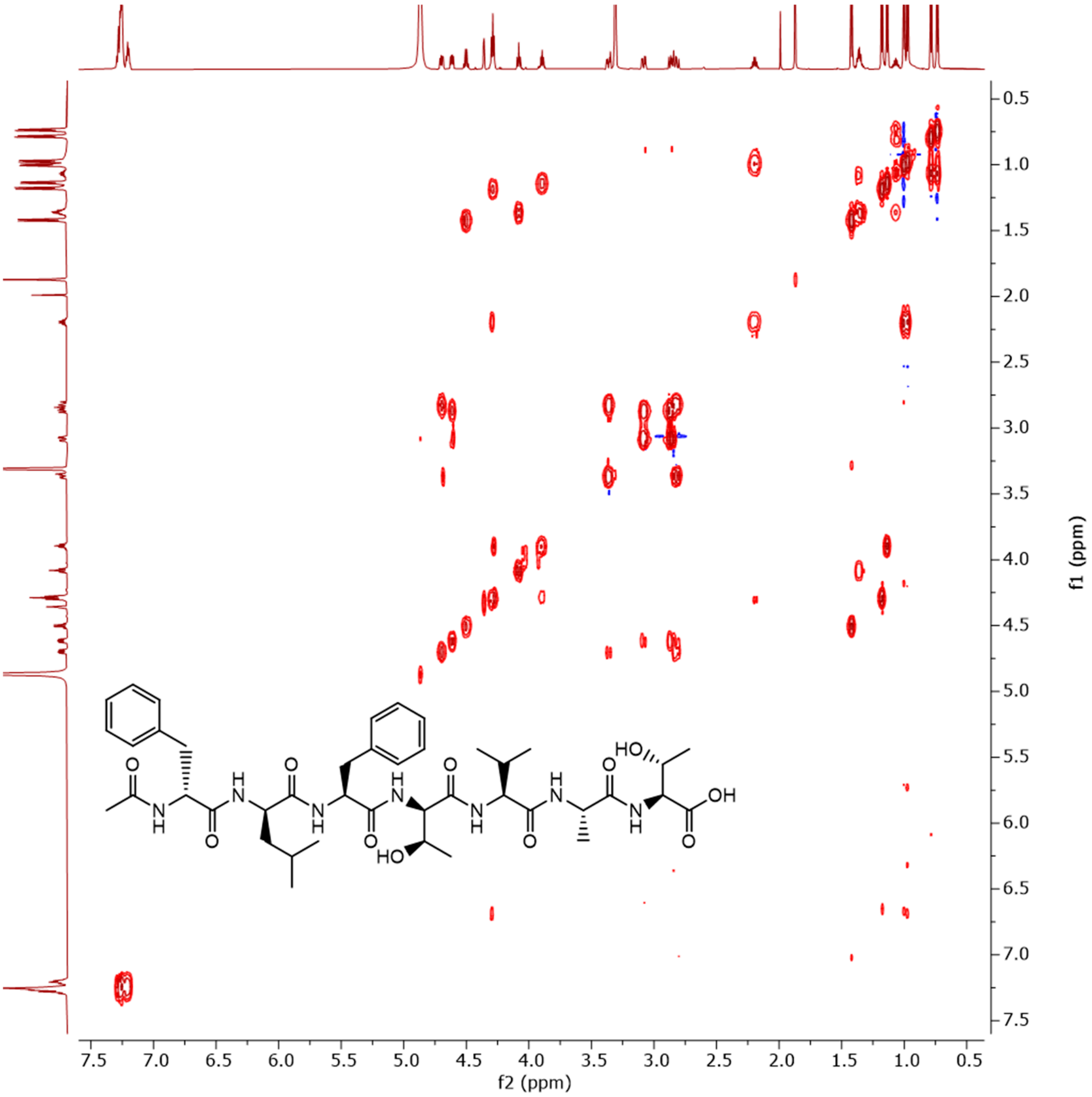

Figure S77. ^{1}H-^{1}H COSY NMR spectrum (600 MHz, CD_3OD) of nobilamide S (**18**).

^1H-^{13}C HSQC NMR spectrum (600 MHz, CD_3OD) of nobilamide S (18)

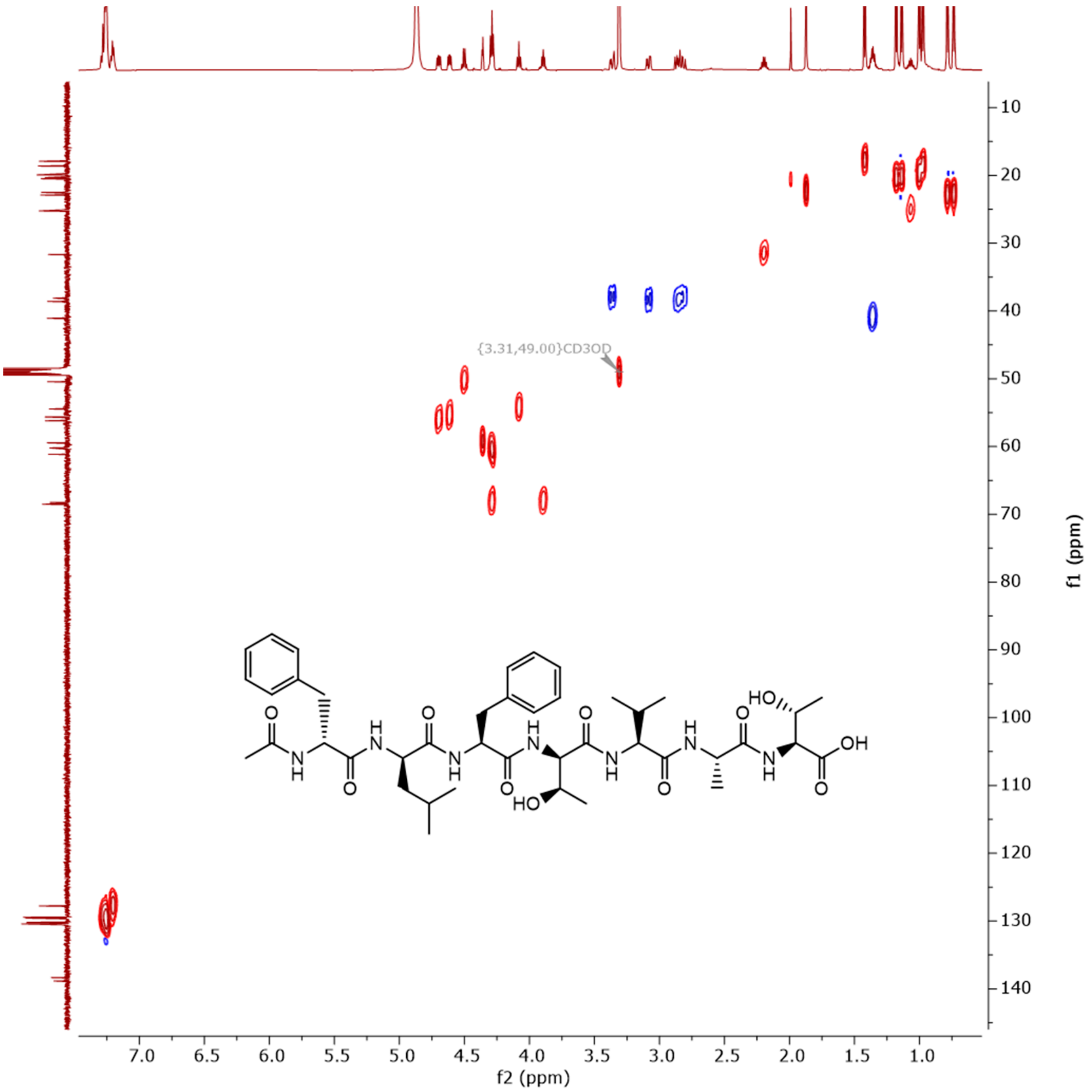

Figure S78. ^1H-^{13}C HSQC NMR spectrum (600 MHz, CD_3OD) of nobilamide S (**18**).

^{1}H-^{13}C HMBC NMR spectrum (600 MHz, CD_3OD) of nobilamide S (18)

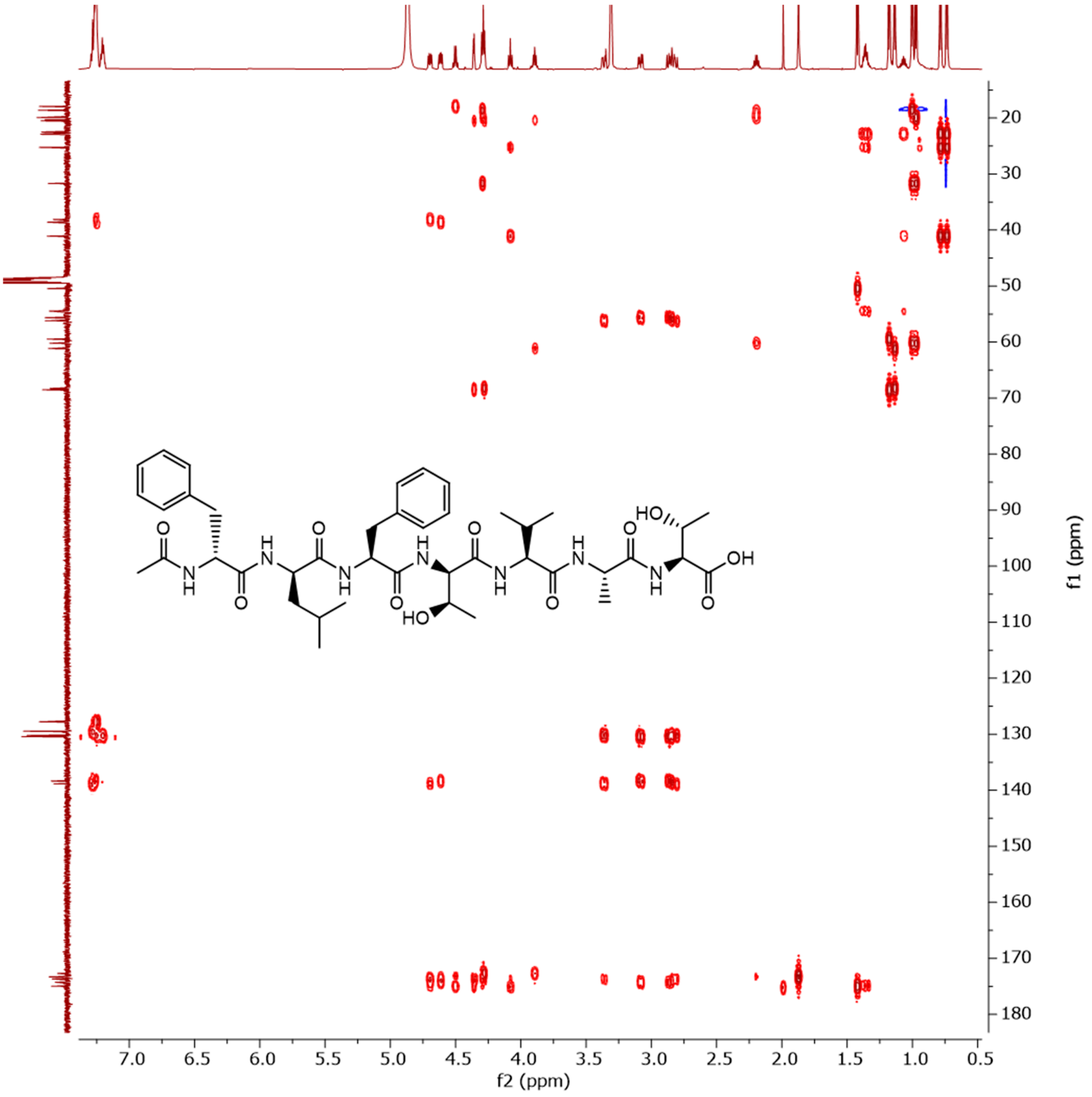

Figure S79. ^{1}H-^{13}C HMBC NMR spectrum (600 MHz, CD_3OD) of nobilamide S (**18**).

Key ^1H-^1H COSY and ^1H-^{13}C HMBC NMR spectrum of nobilamide S (18)

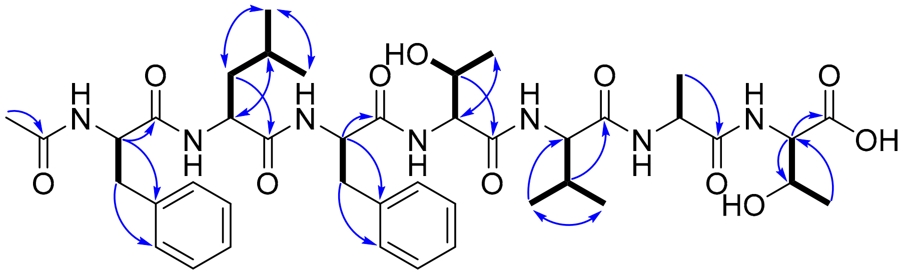

Figure S80. Key ^1H-^1H COSY and ^1H-^{13}C HMBC NMR spectrum (600 MHz, CD_3OD) of nobilamide S (**18**).

NMR data of nobilamide S (18)

4.8 mg, pale white amorphous powder.

^1H-NMR (600 MHz, CD_3OD): δ (ppm): 0.74 (d, *J* = 6.5 Hz, 3H), 0.79 (d, *J* = 6.5 Hz, 3H), 0.97 (d, *J* = 6.8 Hz, 3H), 1.00 (d, *J* = 6.8 Hz, 3H), 1.07 (dp, *J* = 13.4, 6.8 Hz, 1H), 1.14 (d, *J* = 6.4 Hz, 3H), 1.18 (d, *J* = 6.4 Hz, 3H), 1.36 (dq, *J* = 11.1, 6.4 Hz, 2H), 1.42 (d, *J* = 7.1 Hz, 3H), 1.87 (s, 3H), 2.20 (h, *J* = 6.8 Hz, 1H), 2.82 (dd, *J* = 14.1, 11.1 Hz, 1H), 2.86 (dd, *J* = 13.9, 9.1 Hz, 1H), 3.08 (dd, *J* = 14.1, 4.8 Hz, 1H), 3.36 (dd, *J* = 14.0, 4.5 Hz, 1H), 3.89 (p, *J* = 6.2 Hz, 1H), 4.08 (t, *J* = 7.7 Hz, 1H), 4.27 – 4.31 (m, 3H), 4.36 (d, *J* = 3.3 Hz, 1H), 4.50 (q, *J* = 7.2 Hz, 1H), 4.62 (dd, *J* = 9.3, 4.8 Hz, 1H), 4.70 (dd, *J* = 11.0, 4.7 Hz, 1H), 7.18 – 7.22 (m, 2H), 7.24 – 7.30 (m, 8H).

^{13}C-NMR (151 MHz, CD_3OD): δ (ppm): 17.92, 18.62, 19.92, 20.32, 20.50, 22.50, 22.80, 22.90, 25.24, 31.68, 38.13, 38.59, 41.09, 50.44, 54.45, 55.64, 56.19, 59.46, 60.21, 61.15, 68.26, 68.50, 127.78, 129.44, 129.57, 130.20, 130.42, 138.36, 138.84, 172.70, 173.28, 173.30, 173.68, 173.74, 174.28, 174.89, 174.98.

Nobilamide T (19)

HR-ESI-MS of nobilamide T (19)

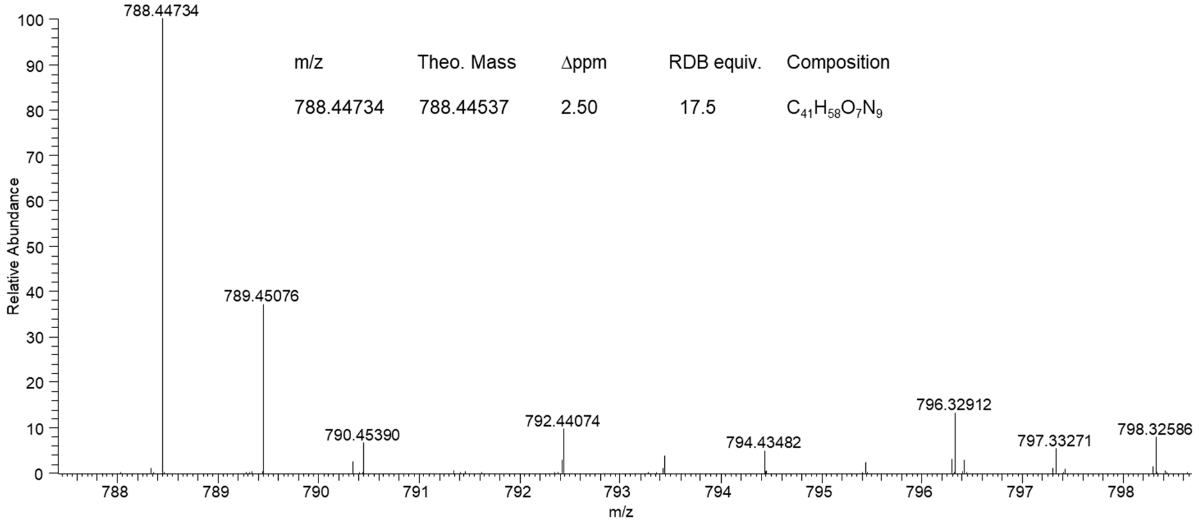

Figure S81. HR-ESI-MS of nobilamide T (**19**), retention time: 14.3 min (HPLC method: LC-HR-ESI-MS).

ESI-MS/MS of nobilamide T (19)

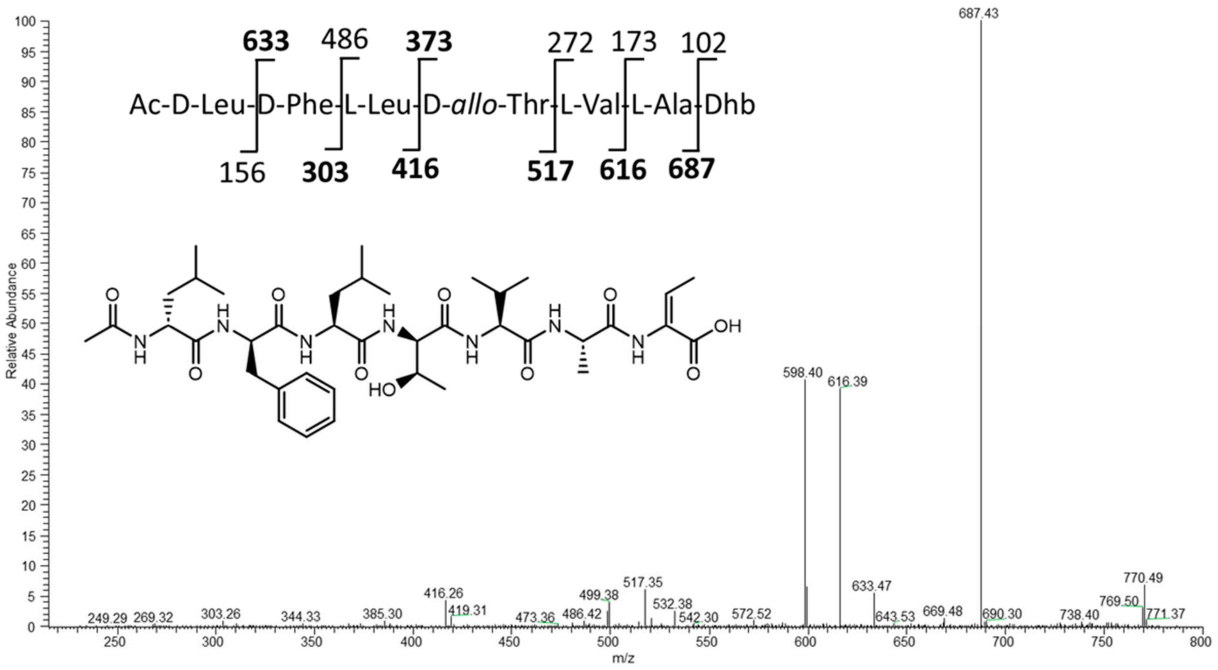

Figure S82. ESI-MS/MS of nobilamide T (**19**). MS/MS of the quasimolecular ion $[M+H]^+$ m/z 788.4 of nobilamide T (**19**). The structures of **19**, and the y and b fragments of **19** are presented. The observed fragments are highlighted in bold. Dhb: *Z*-α,β-dehydrobutyrine.

Stereochemistry of the amino acids of the mixture of nobilamide T (19)

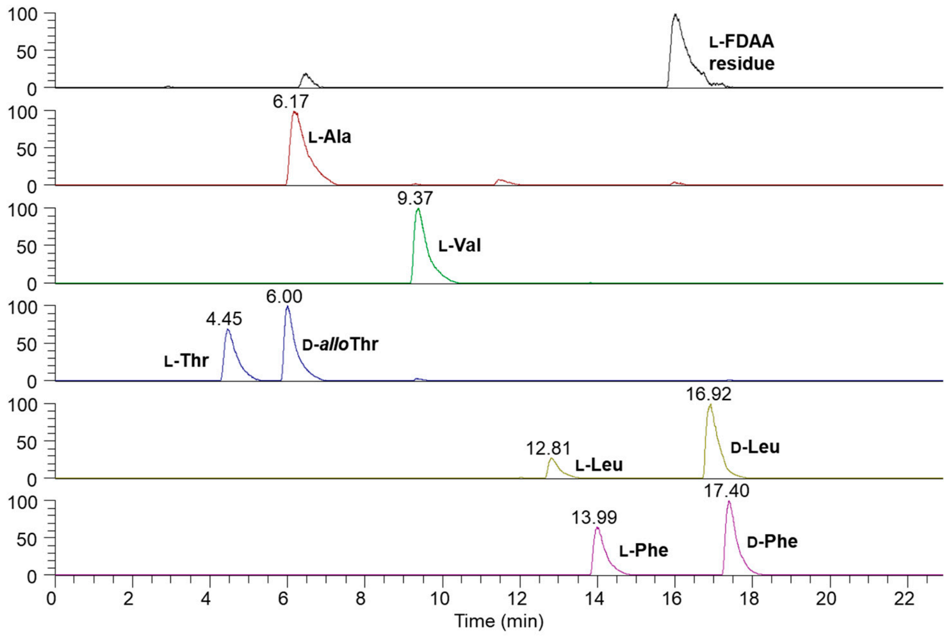

Figure S83. Stereochemistry of the amino acids of nobilamide T (**19**). The stereochemistry of the amino acids of nobilamide T (**19**) was analysed by LC-MS after acid hydrolysis of nobilamide T (**19**) and derivatization with Marfey's reagent **[37,38]**.

The most likely stereochemistry of nobilamide T (**19**) is presented based on the Marfey analysis and the established stereochemistry of nobilamides at the respective positions in the peptide sequence. There is a minor nobilamide variant accompanying nobilamide T (**19**) and most likely a contamination from another (nobilamide) peptide because of the occurrence of L-threonine.

Nobilamide U (20) and nobilamide V (21)

HR-ESI-MS of nobilamide U (20) and nobilamide V (21)

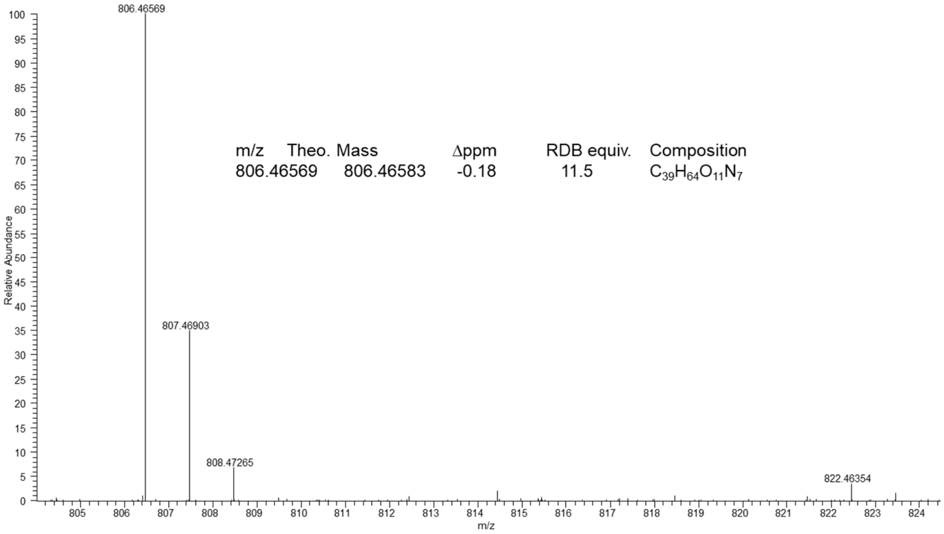

Figure S84. HR-ESI-MS of nobilamide U (**20**) and nobilamide V (**21**), 13.8 min (HPLC method: LC-HR-ESI-MS).

ESI-MS/MS of nobilamide U (20) and nobilamide V (21)

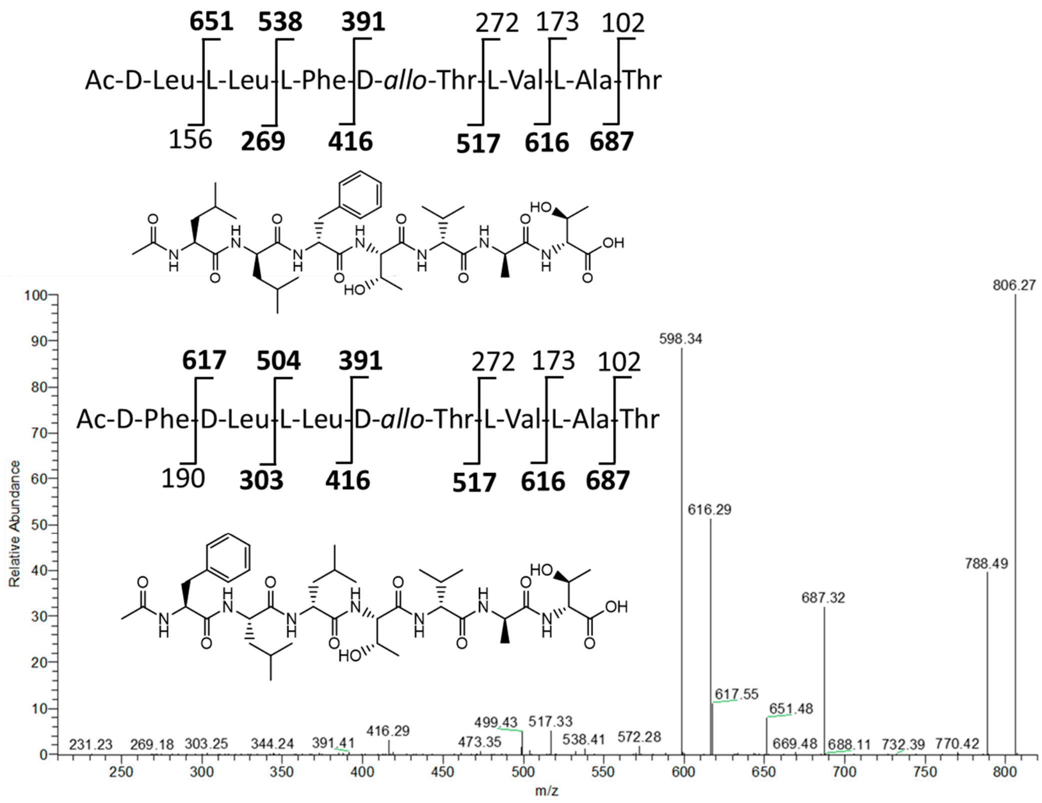

Figure S85. ESI-MS/MS of nobilamide U (**20**) and nobilamide V (**21**). MS/MS of the quasimolecular ion $[M+H]^+$ *m/z* 806.3 of nobilamide U (**20**) and nobilamide V (**21**). The structures of **20** and **21**, and the y and b ion fragments of **20** and **21** are presented. The observed fragments are highlighted in bold.

Stereochemistry of the amino acids the mixture of nobilamide U (20) and nobilamide V (21)

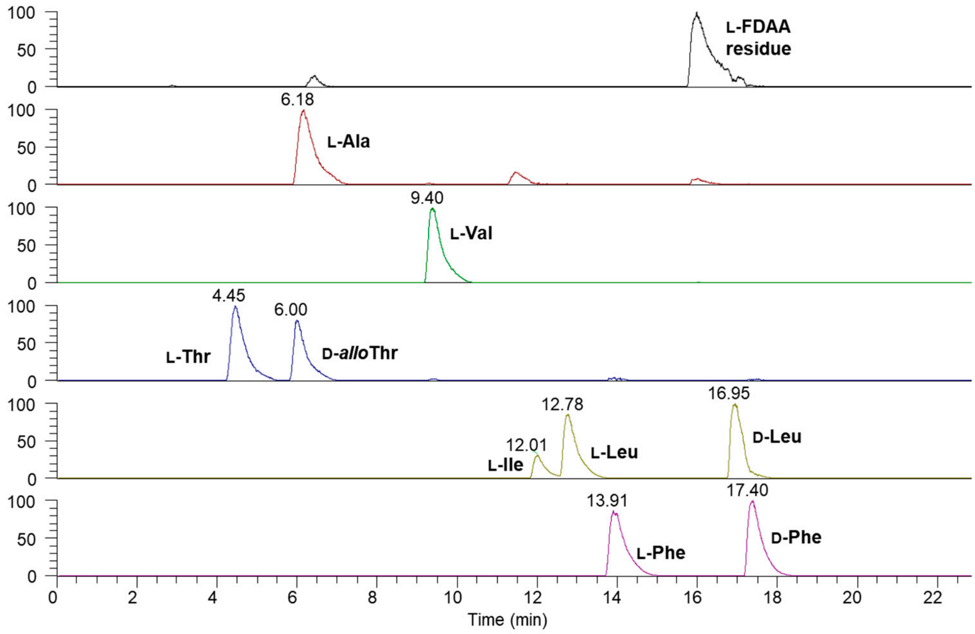

Figure S86. Stereochemistry of the amino acids the mixture of nobilamide U (**20**) and nobilamide V (**21**). The stereochemistry of the amino acids of nobilamide U (**20**) and nobilamide V (**21**) was analysed by LC-MS after acid hydrolysis of nobilamide V (**20**) and nobilamide W (**21**) and derivatization with Marfey's reagent **[37,38]**.

The most likely stereochemistry of the peptides, nobilamide U (**20**) and nobilamide V (**21**), is presented based on the Marfey analysis and the established stereochemistry of nobilamides at the respective positions in the peptide sequence.

Nobilamide W (22)

HR-ESI-MS of nobilamide W (22)

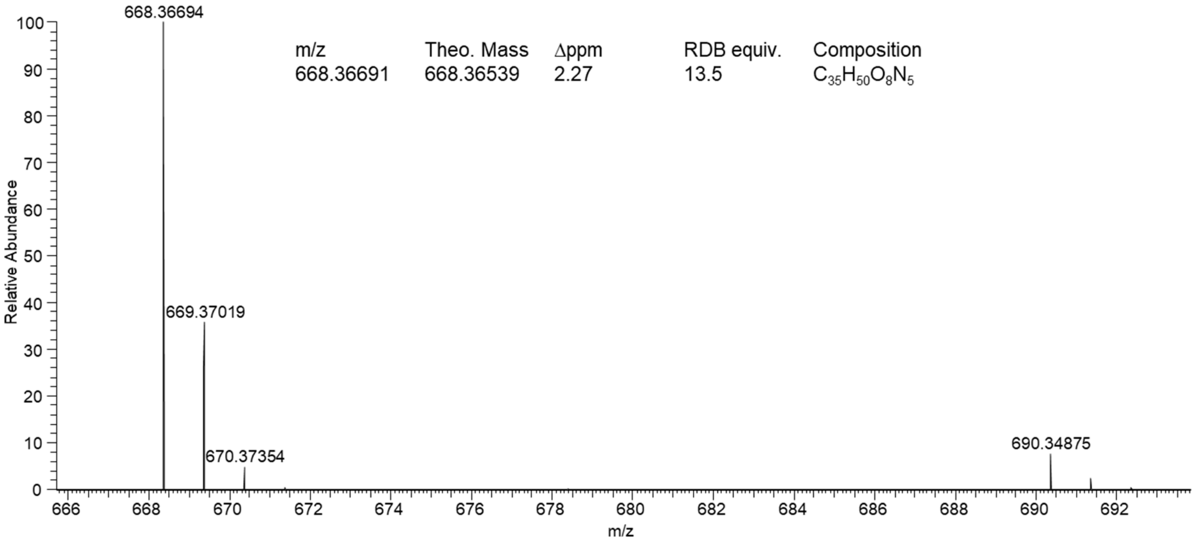

Figure S87. HR-ESI-MS of nobilamide W (**22**), retention time: 16.0 min (HPLC method: LC-HR-ESI-MS).

ESI-MS/MS of nobilamide W (22)

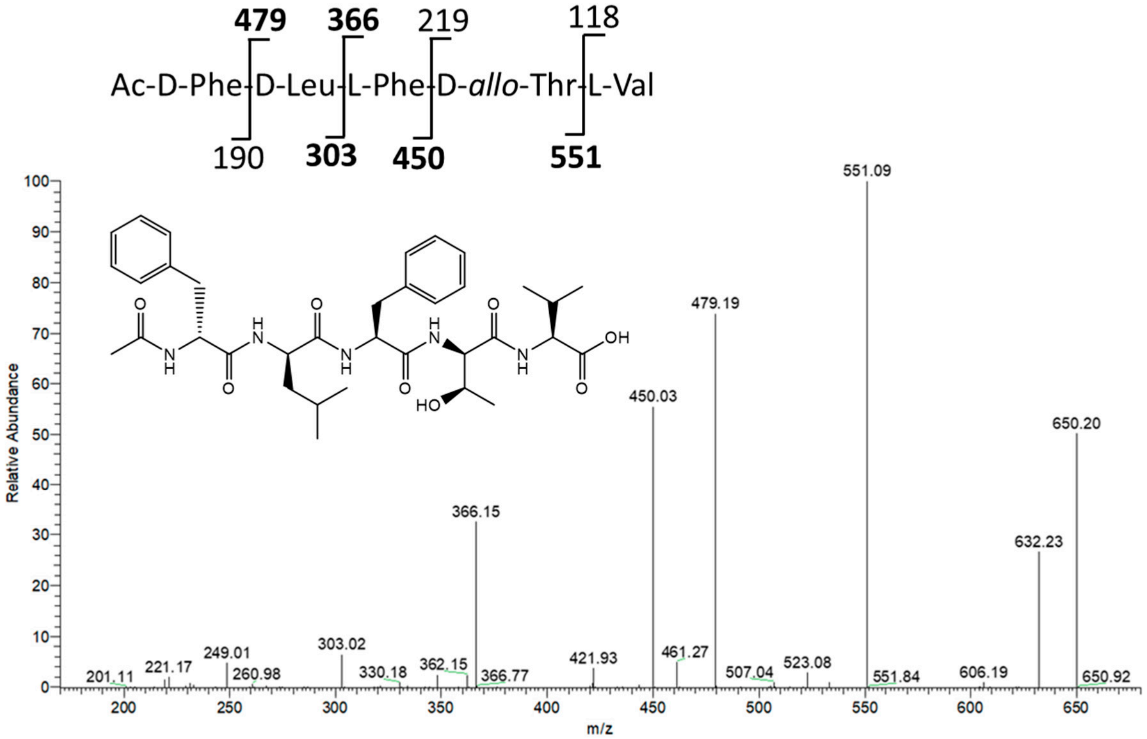

Figure S88. ESI-MS/MS of nobilamide W (**22**). MS/MS of the quasimolecular ion [M+H]$^+$ *m/z* 668.4 of nobilamide W (**22**). The structure **22**, and the y and b ion fragments of **22** are presented. The observed fragments are highlighted in bold.

Stereochemistry of the amino acids of nobilamide W (22)

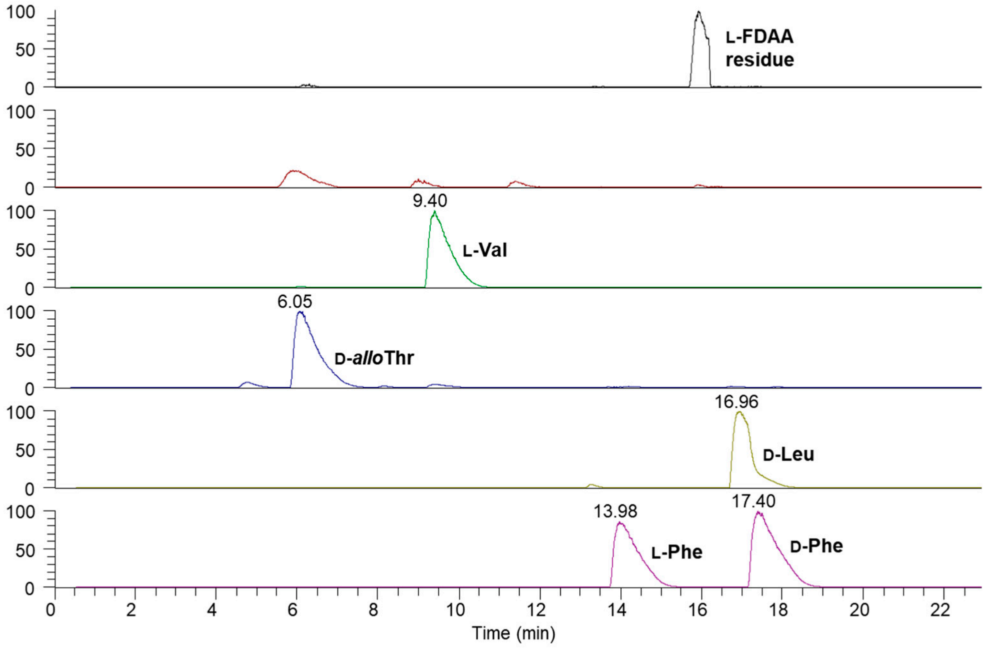

Figure S89. Stereochemistry of the amino acids of nobilamide W (**22**). The stereochemistry of the amino acids of nobilamide W (**22**) was analysed by LC-MS after acid hydrolysis of nobilamide W (**22**) and derivatization with Marfey's reagent **[37,38]**.

The most likely stereochemistry of the peptides, nobilamide W (**22**), is presented based on the Marfey analysis and the established stereochemistry of nobilamides at the respective positions in the peptide sequence.

^{1}H NMR spectrum of nobilamide W (22)

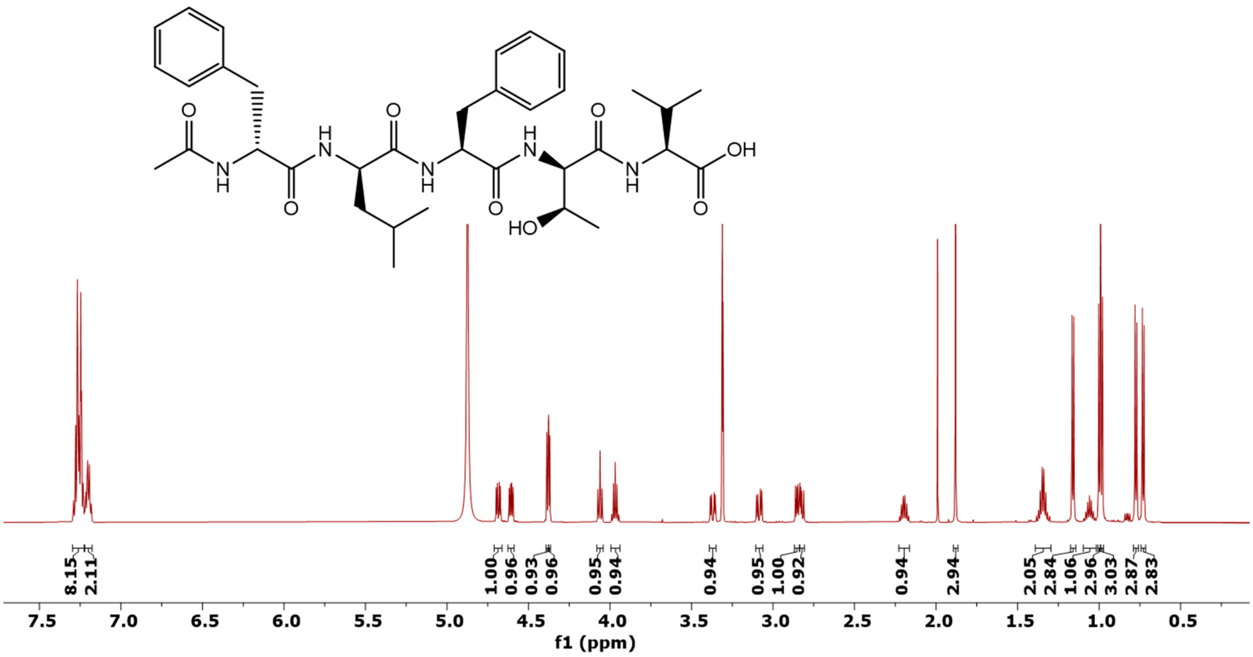

Figure S90. ^{1}H NMR spectrum (600 MHz, CD_3OD) of nobilamide W (**22**).

^{13}C NMR spectrum of nobilamide W (22)

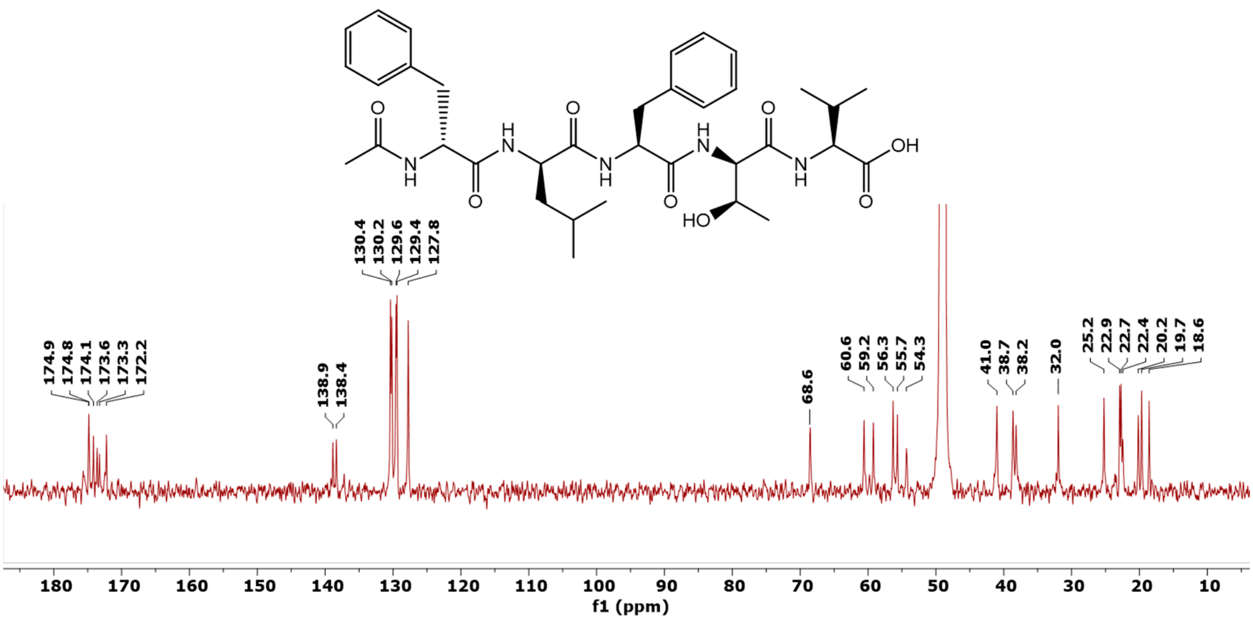

Figure S91. ^{13}C NMR spectrum (151 MHz, CD_3OD) of nobilamide W (**22**).

^{1}H-^{1}H COSY NMR spectrum of nobilamide W (22)

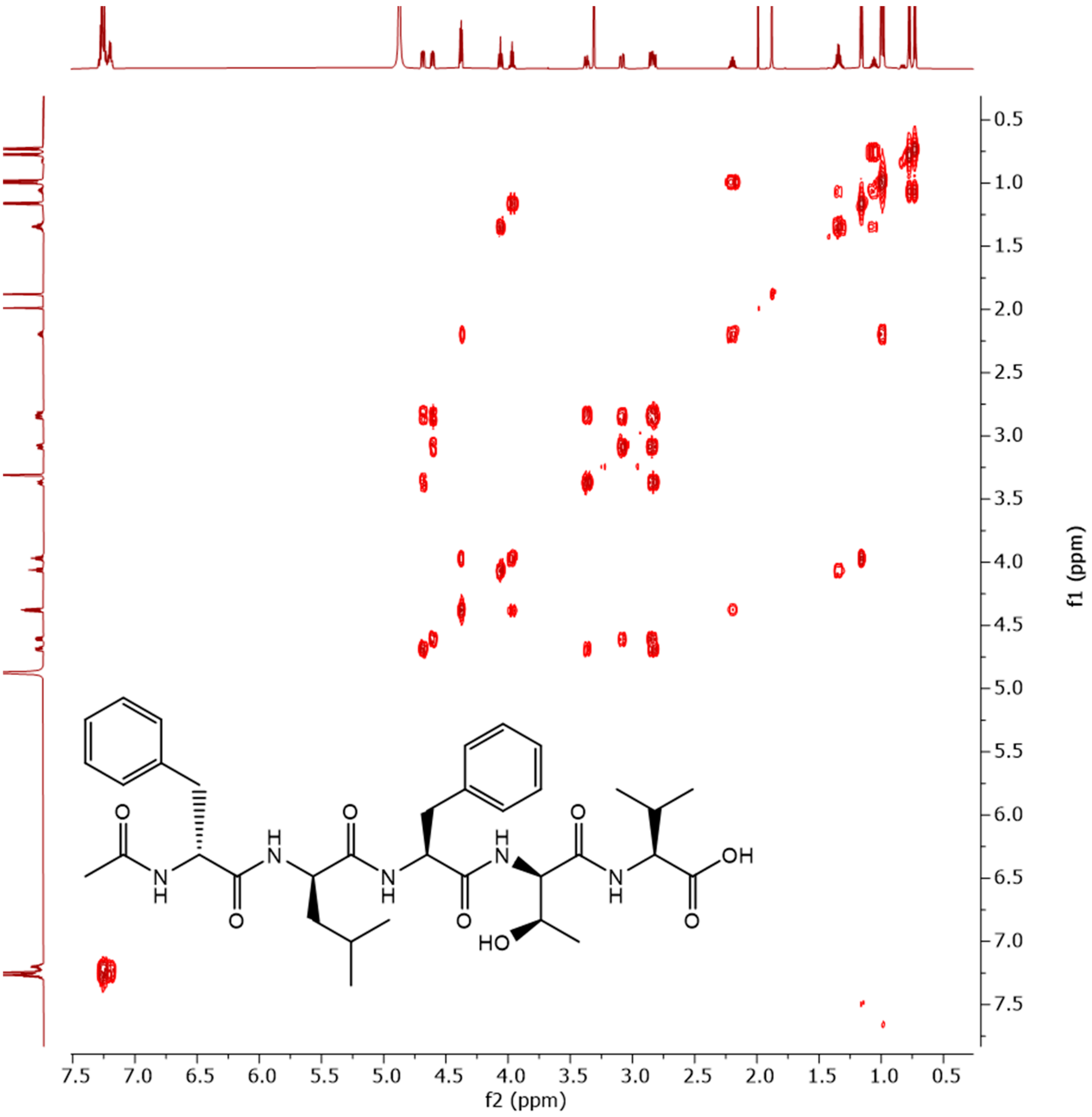

Figure S92. ^{1}H-^{1}H COSY NMR spectrum (600 MHz, CD_3OD) of nobilamide W (**22**).

^{1}H-^{13}C HSQC NMR spectrum of nobilamide W (22)

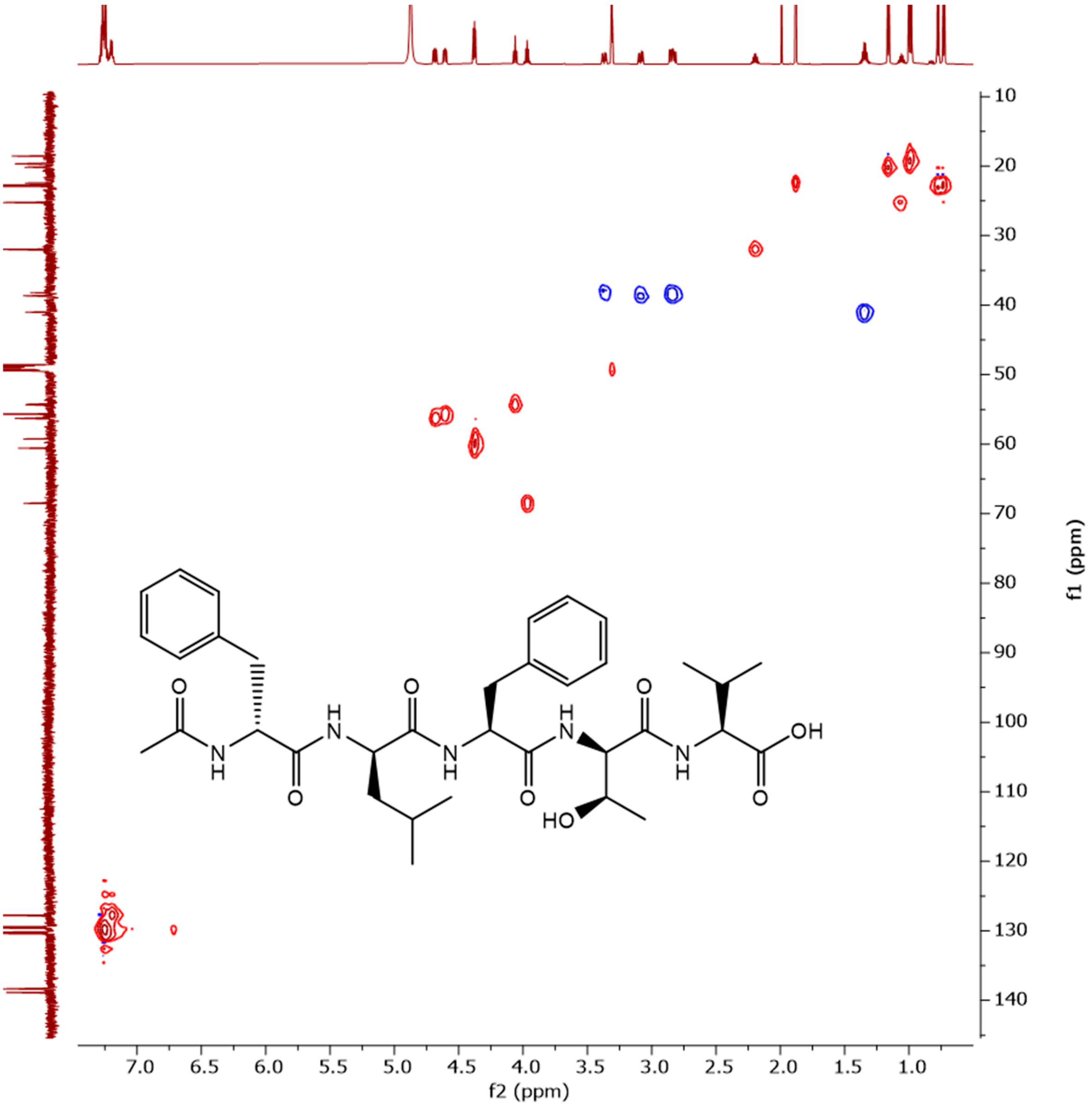

Figure S93. ^{1}H-^{13}C HSQC NMR spectrum (600 MHz, CD_3OD) of nobilamide W (**22**).

^{1}H-^{13}C HMBC NMR spectrum of nobilamide W (22)

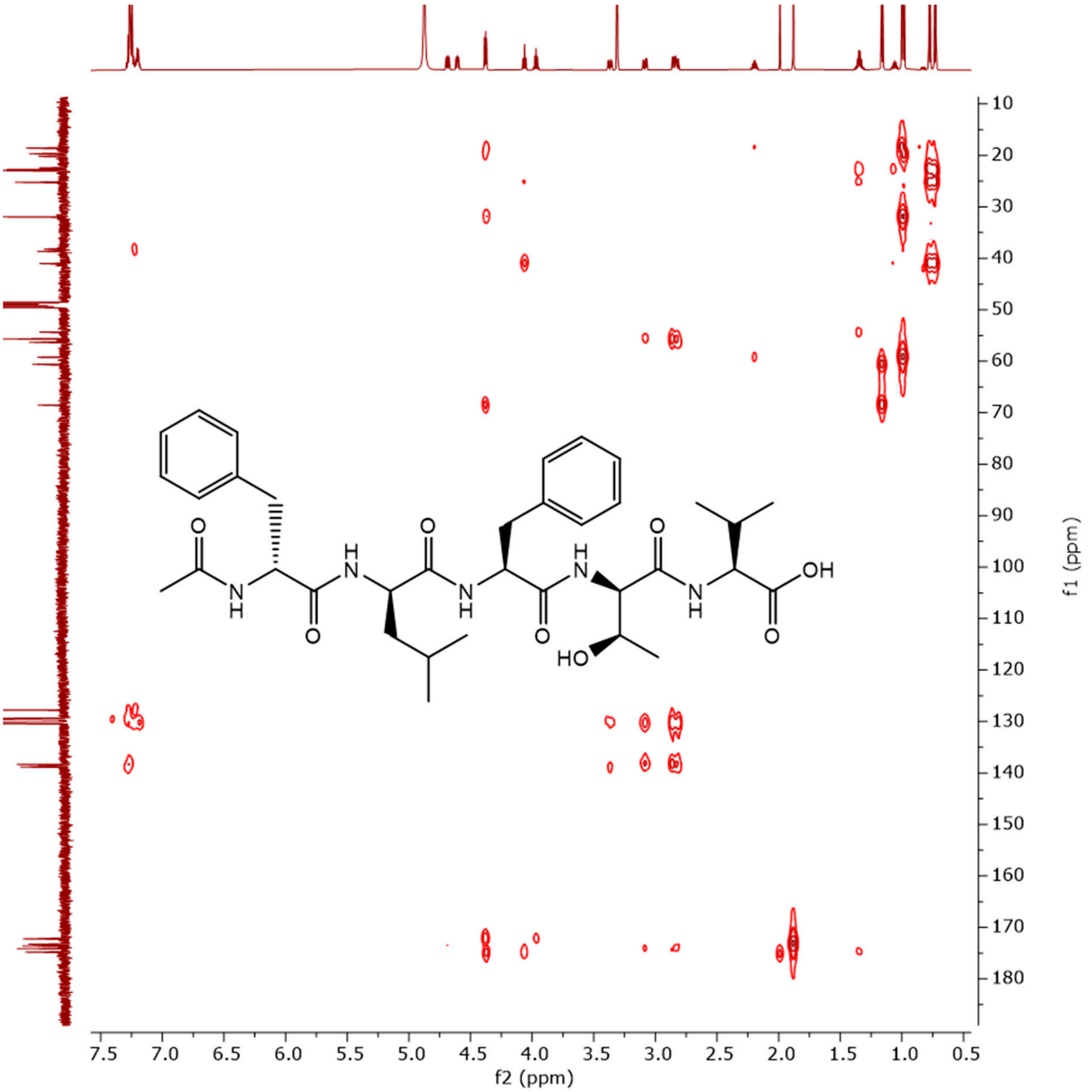

Figure S94. ^{1}H-^{13}C HMBC NMR spectrum (600 MHz, CD_3OD) of nobilamide W (**22**).

Key ^{1}H-^{1}H COSY and ^{1}H-^{13}C HMBC NMR spectrum (600 MHz, CD_3OD) of nobilamide W (22)

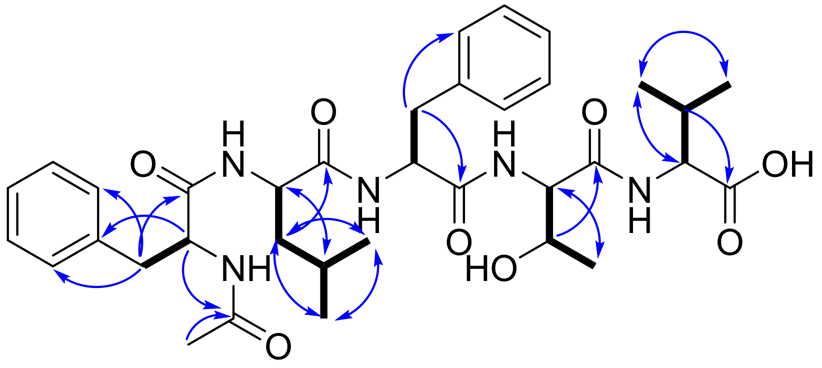

Figure S95. Key ^{1}H-^{1}H COSY and ^{1}H-^{13}C HMBC NMR spectrum (600 MHz, CD_3OD) of nobilamide W (**22**).

NMR data of nobilamide W (22)
3.8 mg, pale white filmy compound.

^{1}H-NMR (600 MHz, CD_3OD): δ (ppm): 0.73 (d, *J* = 6.6 Hz, 3H), 0.77 (d, *J* = 6.5 Hz, 3H), 0.98 (d, *J* = 6.4 Hz, 3H), 1.00 (d, *J* = 6.9 Hz, 3H), 1.06 (dp, *J* = 13.4, 6.9 Hz, 1H), 1.16 (d, *J* = 6.4 Hz, 3H), 1.29 – 1.39 (m, 2H), 1.88 (s, 3H), 2.20 (pd, *J* = 6.9, 5.6 Hz, 1H), 2.81 – 2.84 (m, 1H), 2.85 (dd, *J* = 9.1, 5.0 Hz, 1H), 3.09 (dd, *J* = 14.1, 5.0 Hz, 1H), 3.37 (dd, *J* = 14.1, 4.4 Hz, 1H), 3.97 (p, *J* = 6.4 Hz, 1H), 4.06 (t, *J* = 7.6 Hz, 1H), 4.37 (d, *J* = 4.2 Hz, 1H), 4.38 (d, *J* = 5.2 Hz, 1H), 4.61 (dd, *J* = 9.3, 5.0 Hz, 1H), 4.69 (dd, *J* = 11.2, 4.4 Hz, 1H), 7.18 – 7.22 (m, 2H), 7.23 – 7.30 (m, 8H).

^{13}C-NMR (151 MHz, CD_3OD): δ (ppm): 18.57, 19.67, 20.19, 22.47, 22.71, 22.92, 25.24, 31.97, 38.20, 38.65, 41.01, 54.34, 55.67, 56.30, 59.25, 60.60, 68.59, 127.76, 127.77, 129.42, 129.57, 130.18, 130.38, 138.35, 138.86, 172.23, 173.26, 173.60, 174.14, 174.81, 174.86.

Agar diffusion assays of nobilamide peptides against microbial pathogens

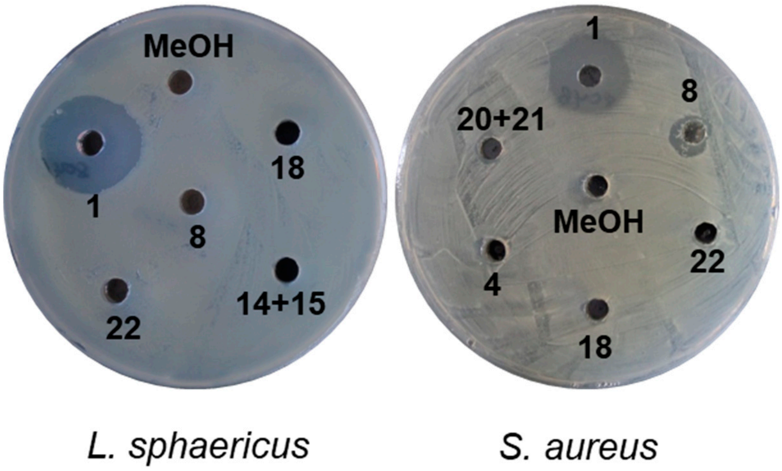

Figure S96. Agar diffusion assays of nobilamide peptides against microbial pathogens. The antibiotic activity against selected microbial pathogens of selected A-3302B (**1**), nobilamide A (**4**), nobilamide I (**8**), nobilamide O (**14**) and nobilamide P (**15**), nobilamide S (**18**), nobilamide U (**20**) and nobilamide V (**21**), as well as nobilamide W (**22**) peptides was determined. Only A-3302-B (**1**) inhibited the growth of *L. sphaericus* and *S. aureus* (inhibition zone: 18.5 mm ± 0.7 mm) at 7 µg/hole. Legend: M: methanol. Numbers of nobilamides are given beside the respective hole in the agar diffusion assay pictures.

Agar diffusion assays of nobilamide peptides against *L. sphaericus*

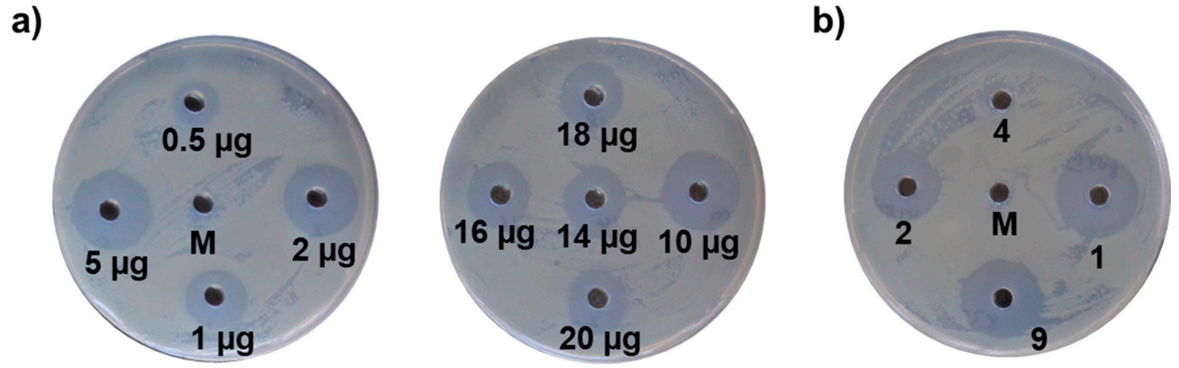

Figure S97. Pictures of agar diffusion assays of nobilamide peptides against *L. sphaericus* in different amounts/hole. A) Testing A-3302-B (**1**) against *L. sphaericus* from 0.5 µg/hole to 20 µg/hole. Maximum inhibitory activity was observed at 5 µg/hole. B) Inhibition of *L. sphaericus* (5 µg/hole) by A-3302-B (**1**, inhibition zone: 21.7 mm ± 0.6 mm), A-3302-A (**2**, inhibition zone: 18.3 mm ± 1.5 mm) and nobilamide J (**9**, inhibition zone: 21.3 mm ± 0.6) which differed only in their acyl chain length. Nobilamide A (**4**) did not exhibit antimicrobial activity against *L. sphaericus.* Legend: M: methanol. Amounts tested (a) or numbers of nobilamides (b) are given beside the respective hole in the agar diffusion assay pictures.

Diameter of inhibition zones caused by nobilamides against *L. sphaericus*

Table S1. Diameter of inhibition zones caused by A-3302-B (1) against *L. sphaericus* in agar diffusion assays

n = 3

**Amount/hole µg/hole**	**Diameter inhibition zone and standard deviation in mm**
0	0± 0.0
0.05	0±0.0
0.5	11.7±0.6
2	20.3±0.6
5	21.3±0.6
14	17.7±0.6
16	17.3±1.2
18	16.3±0.6
20	16±0.0
50	0±0.0
100	0±0.0

Effect of nobilamides on biofilm formation of selected microorganisms

Table S2. Means and standard deviation values for the biofilm assays

n = 3, Absorbance at 590 nm, Nob. : nobilamide

**Sample**	**Mean**	**Std. dev.**
B. amyloliquefaciens		
Medium	0.0595	0.02821938
Control	0.267	0.0895377
Nob. W (**22**)	0.16666667	0.01209683
A-3302-B (**1**)	0.13833333	0.02579406
Nob. A (**4**)	0.1645	0.08697413
Nob. I (**8**)	0.16375	0.04026061
Nob. S (**18**)	0.11266667	0.00665833
Bacillus sp. G2112		
Medium	0.05335	0.004458
Control	0.122	0.065786
Nob. W (**22**)	0.160725	0.131107
A-3302-B (**1**)	0.286825	0.157077
Nob. A (**4**)	0.08055	0.016186
Nob. I (**8**)	0.17965	0.052969
Nob. S (**18**)	0.114925	0.050093
L. sphaericus		
Medium	0.032	0
Control	0.07325	0.0005
Nob. W (**22**)	0.06525	0.004272
A-3302-B (**1**)	0.0705	0.00526
Nob. A (**4**)	0.06925	0.004193
Nob. I (**8**)	0.06175	0.003594
Nob. S (**18**)	0.065	0.003742
M. aurum		
Medium	0.09866667	0.03023795
Control	1.02533333	0.10693144
Nob. W (**22**)	3.08633333	0.18416388
A-3302-B (**1**)	3.11	0.36025408
Nob. A (**4**)	3.40966667	0.15535872
Nob. I (**8**)	1.68266667	0.65660668

Nob. S (**18**)	2.479	1.13503436

Pseudomonas sp. G124

Medium	0.13175	0.04867152
Control	0.96625	0.12067139
Nob. W (**22**)	1.8055	0.09553184
A-3302-B (**1**)	0.8385	0.1935295
Nob. A (**4**)	1.8335	0.12709445
Nob. I (**8**)	2.2405	0.17799719
Nob. S (**18**)	2.22325	0.08991616

P. syringae pv. *glycinea*

Medium	0.05366667	0.0011547
Control	0.62733333	0.0911336
Nob. W (**22**)	2.955	0.59939052
A-3302-B (**1**)	3.82133333	0.24891029
Nob. A (**4**)	3.395	0.52675706
Nob. I (**8**)	2.41133333	1.29652867
Nob. S (**18**)	0.575	0.11194642

References

36. Wang, M.; Carver, J.J.; Phelan, V. V.; Sanchez, L.M.; Garg, N.; Peng, Y.; Nguyen, D.D.; Watrous, J.; Kapono, C.A.; Luzzatto-Knaan, T.; et al. Sharing and Community Curation of Mass Spectrometry Data with Global Natural Products Social Molecular Networking. *Nat. Biotechnol.* **2016**, *34*, 828–837.

37. Marfey, P. Determination of D-Amino Acids. II. Use of a Bifunctional Reagent, 1,5-Difluoro-2,4-Dinitrobenzene. *Carlsb. Res. Commun.* **1984**, *49*, 591–596.

38. Ayon, N.J.; Sharma, A.D.; Gutheil, W.G. LC-MS/MS-Based Separation and Quantification of Marfey's Reagent Derivatized Proteinogenic Amino Acid DL-Stereoisomers. *J Am Soc Mass Spectrom.* **2019**, *30*, 448–458.
